# Supplementary material for: Evidence for the major role of PH4αEFB in the prolyl 4-hydroxylation of Drosophila collagen IV
Source: Matrix Biol. Author manuscript; Available in PMC 2025 Dec 28. (PMC12744885; doi:10.1016/j.matbio.2025.09.002)
Supplement: Fig S4 [file NIHMS2129658-supplement-Fig_S4.pdf]

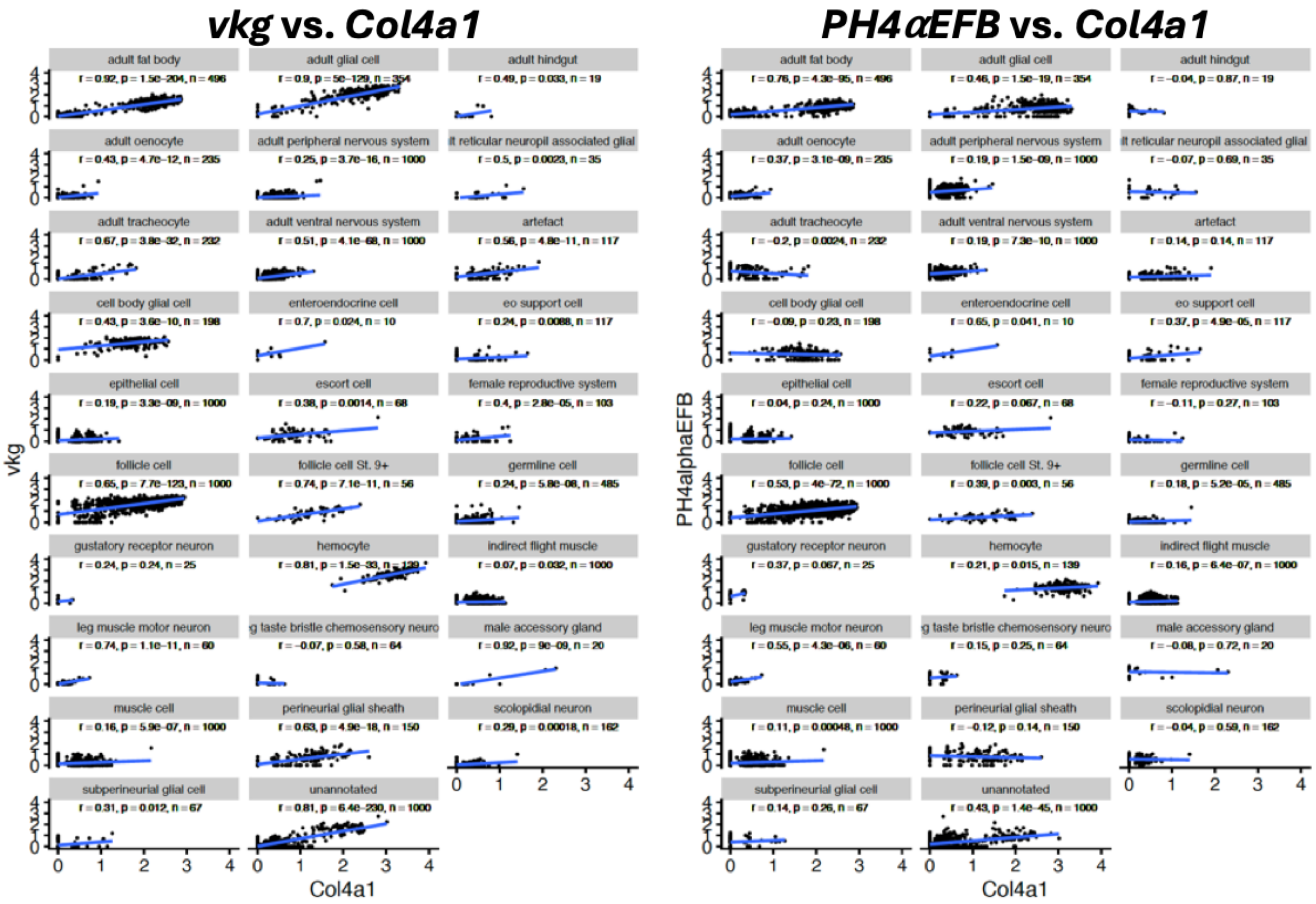

Fig. S4. Expression of *Col4a1*, *vkg,* or *PH4αEFB* separately analysed for each different cell type in the whole-body dataset.

Regression lines of the plots, correlation coefficients between the two genes plotted (*r*), the *p* values to obtain the results from the null hypothesis that the slope of the regression lines are zero, and the numbers of metacells analysed (n) are shown.
