# Supplementary material for: Evidence for the major role of PH4αEFB in the prolyl 4-hydroxylation of Drosophila collagen IV
Source: Matrix Biol. Author manuscript; Available in PMC 2025 Dec 28. (PMC12744885; doi:10.1016/j.matbio.2025.09.002)
Supplement: Fig S5 [file NIHMS2129658-supplement-Fig_S5.pdf]

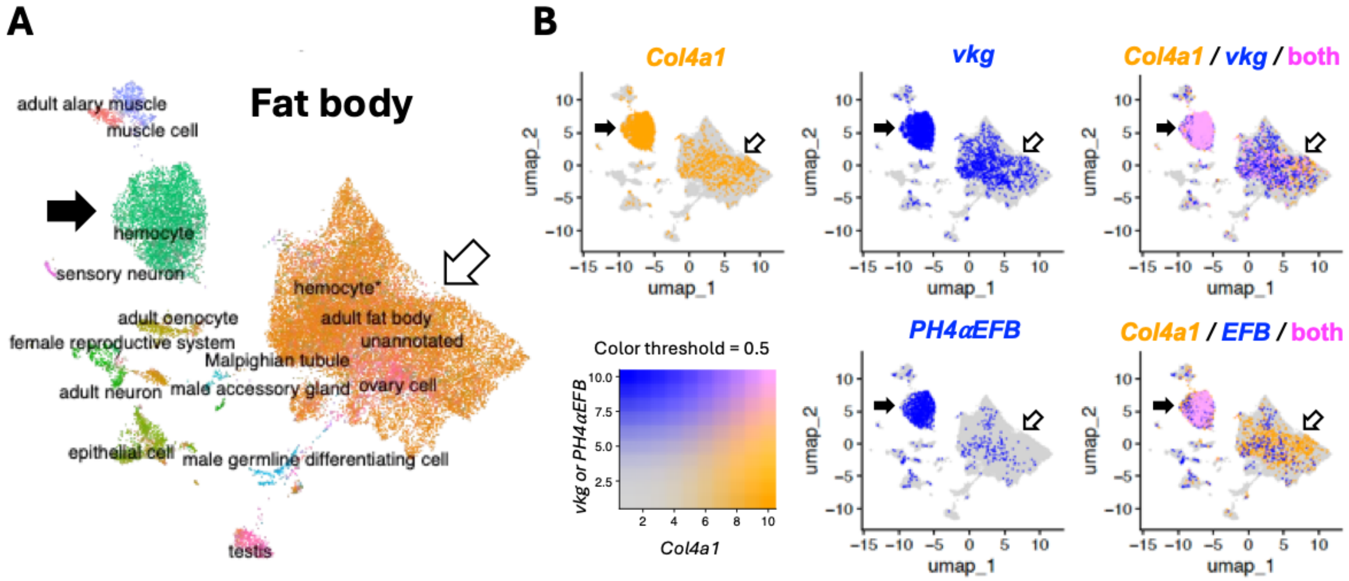

Fig. S5. UMAP for the fat body single cell data.
(A) UMAP showing the entire cells with annotations, with different cell types coded by different colour.
(B) Expression of *Col4a1*, *vkg,* and *PH4αEFB (EFB)* colour coded as in the bottom left panel. Top left and middle panels show single gene expression; right panels show overlap. Closed arrows, hemocytes in which the three genes are co-expressed; open arrows, adult fat body cells in which the co-expression of the collagen IV genes and *PH4αEFB* is not as clear as that in the metacell data in Fig. 3B.
