# Supplementary material for: Evidence for the major role of PH4αEFB in the prolyl 4-hydroxylation of Drosophila collagen IV
Source: Matrix Biol. Author manuscript; Available in PMC 2025 Dec 28. (PMC12744885; doi:10.1016/j.matbio.2025.09.002)
Supplement: Fig S7 [file NIHMS2129658-supplement-Fig_S7.pdf]

Supplementary Source Data.

Identification of gelatine binding proteins by mass spectrometry.

Below follows the full MASCOT search result file that includes all proteins found in the gel band from sliver staining SDS-PAGE gel showing top-left.

Mascot Search Results

PFHR9

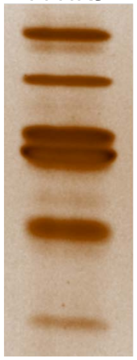

1

```
User                    :
Email                   :
Search title            :
MS data file            : ZIEN-29Nov2017_1m.mgf
Database                : NCBInr 20120419 (17893860 sequences; 6141683785 residues)
Taxonomy                : Mus musculus (house mouse) (138831 sequences)
Timestamp               : 30 Nov 2017 at 20:13:50 GMT
Enzyme                  : Trypsin
Variable modifications  : Carbamidomethyl (C),Oxidation (M),Oxidation (P)
Mass values             : Monoisotopic
Protein Mass            : Unrestricted
Peptide Mass Tolerance  : ± 1.25 Da
Fragment Mass Tolerance : ± 1.001 Da
Max Missed Cleavages    : 1
Instrument type         : Default
Number of queries       : 16088
Protein hits            : gi|23271416  Leprecan 1 [Mus musculus]
                          gi|5852295   lysyl hydroxylase isoform 2 [Mus musculus]
                          gi|387397    epidermal keratin subunit I, partial [Mus musculus]
                          gi|6755106   procollagen-lysine,2-oxoglutarate 5-dioxygenase 1 precursor [Mus musculus]
                          gi|26345382  unnamed protein product [Mus musculus]
                          gi|4159806   type II keratin subunit protein [Mus musculus]
                          gi|1389682   plakoglobin, partial [Mus musculus]
                          gi|387398    epidermal keratin type I, partial [Mus musculus]
                          gi|148708988 mCG20427 [Mus musculus]
                          gi|12859782  unnamed protein product [Mus musculus]
                          gi|16303309  type II keratin 5 [Mus musculus]
                          gi|9790161   plakophilin-1 [Mus musculus]
                          gi|47523977  keratin, type II cytoskeletal 72 [Mus musculus]
                          gi|47059013  keratin, type II cytoskeletal 73 [Mus musculus]
                          gi|836898    prolyl 4-hydroxylase alpha(I)-subunit, partial [Mus musculus]
                          gi|220474    lamin A [Mus musculus]
                          gi|52785     unnamed protein product [Mus musculus]
                          gi|6677775   60S ribosomal protein L22 [Mus musculus]
                          gi|293686    epidermal keratin subunit II [Mus musculus]
                          gi|14917005  RecName: Full=Stress-70 protein, mitochondrial; AltName: Full=75 kDa glucose-regulated protein; Short=GRP-75; AltName: Full=Heat shock 70 kDa protein 9; AltName: Full=
                          gi|387207    heat shock protein [Mus musculus]
                          gi|50797     unnamed protein product [Mus musculus]
                          gi|6755110   procollagen-lysine,2-oxoglutarate 5-dioxygenase 3 precursor [Mus musculus]
                          gi|817996    class I (Qa) Q2k antigen [Mus musculus]
                          gi|19923857  thymidine phosphorylase [Mus musculus]
                          gi|51452     unnamed protein product [Mus musculus]
                          gi|398168    keratin 2 epidermis [Mus musculus]
                          gi|15277319  leucine zipper transcription factor-like protein 1 [Mus musculus]
                          gi|200765    ribonucleotide reductase subunit M1 [Mus musculus]
                          gi|191765    alpha-fetoprotein, partial [Mus musculus]
                          gi|18480346  olfactory receptor MOR175-2 [Mus musculus]
                          gi|226005    protein 40kD
                          gi|193645    glucose-regulated protein 78, partial [Mus musculus]
                          gi|6996913   annexin A2 [Mus musculus]
                          gi|116283284 Por protein [Mus musculus]
                          gi|12805465  Dnajc10 protein, partial [Mus musculus]
                          gi|148678782 mCG1027198, isoform CRA_a [Mus musculus]
                          gi|16716569  protease, serine, 1 precursor [Mus musculus]
                          gi|19924302  ankyrin repeat domain-containing protein 6 [Mus musculus]
                          gi|148680049 mCG147414 [Mus musculus]
                          gi|26337475  unnamed protein product [Mus musculus]
                          gi|199938    tumor-specific myb protein [Mus musculus]
                          gi|22094097  ganglioside-induced differentiation-associated protein 2 [Mus musculus]
                          gi|198683    unnamed protein product [Mus musculus]
                          gi|26327761  unnamed protein product [Mus musculus]
                          gi|51092301  type II keratin Kb14 [Mus musculus]
                          gi|74208129  unnamed protein product [Mus musculus]
                          gi|148685252 mCG142044 [Mus musculus]
                          gi|148671989 mCG1027581 [Mus musculus]
                          gi|26349347  unnamed protein product [Mus musculus]
                          gi|6755893   trypsin 4 precursor [Mus musculus]
                          gi|6754976   peroxiredoxin-1 [Mus musculus]
```

Select Summary Report

Format As | Select Summary (protein hits) | Help

Significance threshold p< 0.05 Max. number of hits AUTO

Standard scoring ◯ MudPIT scoring ◉ Ions score or expect cut-off 43 Show sub-sets 0

Show pop-ups ◉ Suppress pop-ups ◯ Require bold red ☐

Re-Search ◉ All queries ◯ Unassigned ◯ Below homology threshold ◯ Below identity threshold

1. gi|23271416 **Mass:** 83540 **Score:** 243 **Matches:** 11(10) **Sequences:** 8(8) **emPAI:** 0.47
Leprecan 1 [Mus musculus]

Query	Observed	Mr(expt)	Mr(calc)	Delta	Miss	Score	Expect	Rank	Unique	Peptide
11282	1089.6395	1088.6322	1088.5601	0.0721	0	44	0.056	1	U	K.EIETLVEEK.T
11786	567.4809	1132.9471	1132.5360	0.4112	0	50	0.0094	1	U	R.TAIEESQAER.K
13314	644.9944	1287.9741	1287.6241	0.3500	0	60	0.00092	1	U	K.TVTAEVQPQCGR.A
14077	686.7367	1371.4588	1371.6605	-0.2017	0	61	0.0017	1	U	R.GDWPGVVLNMER.A 14085
14212	692.1389	1382.2633	1381.7677	0.4955	1	49	0.02	1	U	K.LGQEGKVPLQSAR.M
15268	743.7113	1485.4080	1485.7212	-0.3132	0	89	2.6e-006	1	U	R.AVGFSSGTENPHGVK.A
15660	773.9469	1545.8792	1545.7133	0.1659	0	61	0.0018	1	U	R.QNLDYYQTMSGVK.E
15748	781.8881	1561.7616	1561.7083	0.0533	0	(55)	0.0066	1	U	R.QNLDYYQTMSGVK.E
14531	705.4221	2113.2444	2113.9826	-0.7382	0	68	0.00038	1	U	K.AVAAAHTFFVGNPEHMEMR.Q
14649	710.9669	2129.8789	2129.9775	-0.0986	0	(45)	0.068	1	U	K.AVAAAHTFFVGNPEHMEMR.Q

Proteins matching the same set of peptides:

gi|26326437 **Mass:** 83476 **Score:** 243 **Matches:** 11(10) **Sequences:** 8(8)
unnamed protein product [Mus musculus]
gi|74205527 **Mass:** 84439 **Score:** 243 **Matches:** 11(10) **Sequences:** 8(8)
unnamed protein product [Mus musculus]
gi|109150433 **Mass:** 83598 **Score:** 243 **Matches:** 11(10) **Sequences:** 8(8)
prolyl 3-hydroxylase 1 isoform 2 precursor [Mus musculus]
gi|109150437 **Mass:** 84426 **Score:** 243 **Matches:** 11(10) **Sequences:** 8(8)
prolyl 3-hydroxylase 1 isoform 1 precursor [Mus musculus]
gi|148698509 **Mass:** 84378 **Score:** 243 **Matches:** 11(10) **Sequences:** 8(8)
leprecan 1, isoform CRA_a [Mus musculus]
gi|148698510 **Mass:** 83550 **Score:** 243 **Matches:** 11(10) **Sequences:** 8(8)
leprecan 1, isoform CRA_b [Mus musculus]
gi|12846125 **Mass:** 84033 **Score:** 243 **Matches:** 11(10) **Sequences:** 8(8)
unnamed protein product [Mus musculus]

---

2. gi|5852295 **Mass:** 84473 **Score:** 198 **Matches:** 9(9) **Sequences:** 7(7) **emPAI:** 0.31
lysyl hydroxylase isoform 2 [Mus musculus]

Query	Observed	Mr(expt)	Mr(calc)	Delta	Miss	Score	Expect	Rank	Unique	Peptide
10580	1017.5937	1016.5864	1016.5906	-0.0042	0	46	0.035	1	U	R.EFIAPVTLK.V
14359	697.9323	1393.8501	1393.6474	0.2027	0	77	2.2e-005	1	U	R.SEDYVDIVQGNR.V 14365
14882	723.4815	1444.9483	1443.7762	1.1721	0	52	0.012	1	U	K.IVFAADGLLWPDK.R
15863	800.6402	1599.2658	1599.8773	-0.6115	1	81	1.5e-005	1	U	K.IVFAADGLLWPDKR.L 15868
15907	809.6948	1617.3751	1616.8774	0.4977	0	55	0.0063	1	U	K.IFQALNGATDEVVLK.F
15920	813.1936	1624.3726	1623.8885	0.4841	0	46	0.039	1	U	K.QIGLENVWLHFIR.E
15034	731.9530	2192.8372	2192.1477	0.6895	1	58	0.0041	1	U	K.IFQALNGATDEVVLKFENGK.S

Proteins matching the same set of peptides:

gi|18204027 **Mass:** 84389 **Score:** 198 **Matches:** 9(9) **Sequences:** 7(7)
Procollagen lysine, 2-oxoglutarate 5-dioxygenase 2 [Mus musculus]
gi|148688975 **Mass:** 84401 **Score:** 198 **Matches:** 9(9) **Sequences:** 7(7)
procollagen lysine, 2-oxoglutarate 5-dioxygenase 2, isoform CRA_a [Mus musculus]
gi|148688976 **Mass:** 86812 **Score:** 198 **Matches:** 9(9) **Sequences:** 7(7)

procollagen lysine, 2-oxoglutarate 5-dioxygenase 2, isoform CRA_b [Mus musculus]
gi|218931165 **Mass:** 84435 **Score:** 198 **Matches:** 9(9) **Sequences:** 7(7)
procollagen-lysine,2-oxoglutarate 5-dioxygenase 2 isoform 2 precursor [Mus musculus]
gi|218931167 **Mass:** 86846 **Score:** 198 **Matches:** 9(9) **Sequences:** 7(7)
procollagen-lysine,2-oxoglutarate 5-dioxygenase 2 isoform 1 precursor [Mus musculus]

---

3. gi|387397 **Mass:** 57807 **Score:** 143 **Matches:** 3(3) **Sequences:** 2(2) **emPAI:** 0.12
epidermal keratin subunit I, partial [Mus musculus]

Query	Observed	Mr(expt)	Mr(calc)	Delta	Miss	Score	Expect	Rank	Unique	Peptide
14204	691.6060	1381.1974	1380.6408	0.5566	0	74	3e-005	1	U	R.ALEESNYELEGK.I 14193
14318	695.9275	1389.8404	1389.6736	0.1669	0	78	4.2e-005	1	U	K.QSLEASLAETEGR.Y

Proteins matching the same set of peptides:
gi|12852157 **Mass:** 58587 **Score:** 143 **Matches:** 3(3) **Sequences:** 2(2)
unnamed protein product [Mus musculus]
gi|26349141 **Mass:** 52653 **Score:** 143 **Matches:** 3(3) **Sequences:** 2(2)
unnamed protein product [Mus musculus]
gi|26349459 **Mass:** 49471 **Score:** 143 **Matches:** 3(3) **Sequences:** 2(2)
unnamed protein product [Mus musculus]
gi|112983636 **Mass:** 57007 **Score:** 143 **Matches:** 3(3) **Sequences:** 2(2)
keratin, type I cytoskeletal 10 [Mus musculus]
gi|116242600 **Mass:** 57735 **Score:** 143 **Matches:** 3(3) **Sequences:** 2(2)
RecName: Full=Keratin, type I cytoskeletal 10; AltName: Full=56 kDa cytokeratin; AltName: Full=Cytokeratin-10; Short=CK-10; AltName: Full=Keratin, type I cytoskeletal 59 kDa; AltName: Full=Keratin-1

---

4. gi|6755106 **Mass:** 83542 **Score:** 105 **Matches:** 2(1) **Sequences:** 2(1) **emPAI:** 0.08
procollagen-lysine,2-oxoglutarate 5-dioxygenase 1 precursor [Mus musculus]

Query	Observed	Mr(expt)	Mr(calc)	Delta	Miss	Score	Expect	Rank	Unique	Peptide
14836	721.0568	1440.0991	1439.7370	0.3621	0	45	0.068	1	U	K.FLVEYIAPMTEK.L
15866	801.0905	1600.1665	1599.8297	0.3368	0	101	1.6e-007	1	U	R.FLGSGGFIGYAPSLSK.L

Proteins matching the same set of peptides:
gi|74207958 **Mass:** 83514 **Score:** 105 **Matches:** 2(1) **Sequences:** 2(1)
unnamed protein product [Mus musculus]

---

5. gi|26345382 **Mass:** 46296 **Score:** 89 **Matches:** 2(1) **Sequences:** 2(1) **emPAI:** 0.15
unnamed protein product [Mus musculus]

Query	Observed	Mr(expt)	Mr(calc)	Delta	Miss	Score	Expect	Rank	Unique	Peptide
15662	774.1068	1546.1991	1545.7246	0.4745	0	87	4.4e-006	1	U	R.MISFSSGGENPHGVK.A
15901	807.7070	2420.0993	2420.1437	-0.0444	0	45	0.066	1	U	K.LAAEGLGFSYAEPNYWISYGGR.Q

Proteins matching the same set of peptides:
gi|148665269 **Mass:** 61664 **Score:** 89 **Matches:** 2(1) **Sequences:** 2(1)
leprecan-like 1 [Mus musculus]
gi|27734076 **Mass:** 80104 **Score:** 89 **Matches:** 2(1) **Sequences:** 2(1)
prolyl 3-hydroxylase 2 precursor [Mus musculus]

---

6. gi|4159806 **Mass:** 65183 **Score:** 87 **Matches:** 5(5) **Sequences:** 3(3) **emPAI:** 0.16
type II keratin subunit protein [Mus musculus]

Query	Observed	Mr(expt)	Mr(calc)	Delta	Miss	Score	Expect	Rank	Unique	Peptide
11211	542.0804	1082.1462	1081.5920	0.5541	1	49	0.011	1		K.FASFIDKVR.F
14216	692.2543	1382.4940	1383.7034	-1.2094	1	47	0.042	1		K.SLNDKFASFIDK.V 14237 14244
15155	738.6367	1475.2588	1474.8144	0.4444	1	61	0.0015	2	U	R.FLEQQNKVLQTK.W

---

7. gi|1389682 **Mass:** 68068 **Score:** 87 **Matches:** 2(2) **Sequences:** 2(2) **emPAI:** 0.10
plakoglobin, partial [Mus musculus]

Query	Observed	Mr(expt)	Mr(calc)	Delta	Miss	Score	Expect	Rank	Unique	Peptide
14556	706.2423	1410.4699	1410.8017	-0.3317	0	71	0.00016	1	U	R.ALMGSPQLVAAVVR.T
14926	725.5305	1449.0465	1448.8140	0.2325	0	61	0.0013	1	U	K.LLNQPNQWPLVK.A

Proteins matching the same set of peptides:
gi|28395018 **Mass:** 81749 **Score:** 87 **Matches:** 2(2) **Sequences:** 2(2)
junction plakoglobin [Mus musculus]

---

8. gi|387398 **Mass:** 41454 **Score:** 87 **Matches:** 2(2) **Sequences:** 2(2) **emPAI:** 0.17
epidermal keratin type I, partial [Mus musculus]

Query	Observed	Mr(expt)	Mr(calc)	Delta	Miss	Score	Expect	Rank	Unique	Peptide
10673	514.9652	1027.9159	1028.5866	-0.6707	0	54	0.0037	1	U	R.VLDELTLAR.A
13972	681.9645	1361.9144	1360.6834	1.2310	0	69	0.00028	1	U	R.EVATNSELVQSGK.S

Proteins matching the same set of peptides:
gi|7106335 **Mass:** 48132 **Score:** 87 **Matches:** 2(2) **Sequences:** 2(2)
keratin, type I cytoskeletal 17 [Mus musculus]
gi|13097093 **Mass:** 30629 **Score:** 87 **Matches:** 2(2) **Sequences:** 2(2)
Krt14 protein [Mus musculus]
gi|21489935 **Mass:** 52834 **Score:** 87 **Matches:** 2(2) **Sequences:** 2(2)
keratin, type I cytoskeletal 14 [Mus musculus]
gi|148670626 **Mass:** 54668 **Score:** 87 **Matches:** 2(2) **Sequences:** 2(2)
mCG144006 [Mus musculus]

---

9. gi|148708988 **Mass:** 320241 **Score:** 85 **Matches:** 2(2) **Sequences:** 2(2) **emPAI:** 0.02
mCG20427 [Mus musculus]

Query	Observed	Mr(expt)	Mr(calc)	Delta	Miss	Score	Expect	Rank	Unique	Peptide
11741	565.5758	1129.1370	1128.6026	0.5344	0	59	0.001	1	U	K.IEVLEEELR.L
14281	694.6082	1387.2019	1386.7103	0.4916	0	68	0.00033	1	U	R.LNDSILQATEQR.R

Proteins matching the same set of peptides:
gi|190194418 **Mass:** 332706 **Score:** 85 **Matches:** 2(2) **Sequences:** 2(2)
desmoplakin [Mus musculus]

---

10. gi|12859782 **Mass:** 65586 **Score:** 84 **Matches:** 5(5) **Sequences:** 3(3) **emPAI:** 0.16
unnamed protein product [Mus musculus]

Query	Observed	Mr(expt)	Mr(calc)	Delta	Miss	Score	Expect	Rank	Unique	Peptide
11211	542.0804	1082.1462	1081.5920	0.5541	1	49	0.011	1		K.FASFIDKVR.F
14216	692.2543	1382.4940	1383.7034	-1.2094	1	47	0.042	1		K.SLNDKFASFIDK.V 14237 14244
15155	738.6367	1475.2588	1474.7780	0.4808	0	58	0.0034	3		R.FLEQQNQVLQTK.W

Proteins matching the same set of peptides:
gi|126116585 **Mass:** 65565 **Score:** 84 **Matches:** 5(5) **Sequences:** 3(3)
keratin, type II cytoskeletal 1 [Mus musculus]

---

11. gi|16303309 **Mass:** 61743 **Score:** 84 **Matches:** 3(3) **Sequences:** 2(2) **emPAI:** 0.17
type II keratin 5 [Mus musculus]

Query	Observed	Mr(expt)	Mr(calc)	Delta	Miss	Score	Expect	Rank	Unique	Peptide
11211	542.0804	1082.1462	1081.5920	0.5541	1	49	0.011	1		K.FASFIDKVR.F
14704	713.6212	1425.2279	1424.6680	0.5599	0	69	0.00024	1	U	R.VDALMDEINFMK.M
14999	729.5875	1457.1605	1456.6578	0.5027	0	(49)	0.024	1	U	R.VDALMDEINFMK.M

Proteins matching the same set of peptides:
gi|20911031 **Mass:** 61729 **Score:** 84 **Matches:** 3(3) **Sequences:** 2(2)
keratin, type II cytoskeletal 5 [Mus musculus]

---

12. gi|9790161 **Mass:** 80844 **Score:** 80 **Matches:** 1(1) **Sequences:** 1(1) **emPAI:** 0.04
plakophilin-1 [Mus musculus]

Query	Observed	Mr(expt)	Mr(calc)	Delta	Miss	Score	Expect	Rank	Unique	Peptide

15543	762.9679	1523.9212	1523.7804	0.1408	0	80	1.4e-005	1	U	R.SPNQNVQQAAAGALR.N

Proteins matching the same set of peptides:
gi|148707614 **Mass:** 78853 **Score:** 80 **Matches:** 1(1) **Sequences:** 1(1)
plakophilin 1 [Mus musculus]

---

13. gi|47523977 **Mass:** 56715 **Score:** 71 **Matches:** 2(2) **Sequences:** 2(2) **emPAI:** 0.12
keratin, type II cytoskeletal 72 [Mus musculus]

Query	Observed	Mr(expt)	Mr(calc)	Delta	Miss	Score	Expect	Rank	Unique	Peptide
11211	542.0804	1082.1462	1081.5920	0.5541	1	49	0.011	1		K.FASFIDKVR.F
15155	738.6367	1475.2588	1475.7620	-0.5032	0	66	0.0005	1	U	R.FLEQQNQVLETK.W

---

14. gi|47059013 **Mass:** 58875 **Score:** 65 **Matches:** 3(2) **Sequences:** 3(2) **emPAI:** 0.18
keratin, type II cytoskeletal 73 [Mus musculus]

Query	Observed	Mr(expt)	Mr(calc)	Delta	Miss	Score	Expect	Rank	Unique	Peptide
11211	542.0804	1082.1462	1081.5920	0.5541	1	49	0.011	1		K.FASFIDKVR.F
13933	679.6329	1357.2512	1356.7249	0.5263	0	43	0.1	1	U	R.NLDLDSIIAEVR.A
15155	738.6367	1475.2588	1474.7780	0.4808	0	58	0.0034	3		R.FLEQQNQVLQTK.W

---

15. gi|836898 **Mass:** 59960 **Score:** 64 **Matches:** 1(1) **Sequences:** 1(1) **emPAI:** 0.05
prolyl 4-hydroxylase alpha(I)-subunit, partial [Mus musculus]

Query	Observed	Mr(expt)	Mr(calc)	Delta	Miss	Score	Expect	Rank	Unique	Peptide
15762	783.5424	1565.0702	1564.7522	0.3180	0	64	0.00069	1	U	K.SAWLSGYEDPVVSR.I

Proteins matching the same set of peptides:
gi|26336999 **Mass:** 60848 **Score:** 64 **Matches:** 1(1) **Sequences:** 1(1)
unnamed protein product [Mus musculus]
gi|33859596 **Mass:** 60872 **Score:** 64 **Matches:** 1(1) **Sequences:** 1(1)
prolyl 4-hydroxylase subunit alpha-1 precursor [Mus musculus]
gi|74148153 **Mass:** 51722 **Score:** 64 **Matches:** 1(1) **Sequences:** 1(1)
unnamed protein product [Mus musculus]
gi|74224984 **Mass:** 60886 **Score:** 64 **Matches:** 1(1) **Sequences:** 1(1)
unnamed protein product [Mus musculus]
gi|74225936 **Mass:** 63769 **Score:** 64 **Matches:** 1(1) **Sequences:** 1(1)
unnamed protein product [Mus musculus]

---

16. gi|220474 **Mass:** 47506 **Score:** 62 **Matches:** 1(1) **Sequences:** 1(1) **emPAI:** 0.07
lamin A [Mus musculus]

Query	Observed	Mr(expt)	Mr(calc)	Delta	Miss	Score	Expect	Rank	Unique	Peptide
10676	515.0694	1028.1242	1027.5662	0.5581	0	62	0.00055	1	U	R.LADALQELR.A

Proteins matching the same set of peptides:
gi|52865 **Mass:** 65367 **Score:** 62 **Matches:** 1(1) **Sequences:** 1(1)
unnamed protein product [Mus musculus]
gi|9506843 **Mass:** 52620 **Score:** 62 **Matches:** 1(1) **Sequences:** 1(1)
prelamin-A/C isoform C2 [Mus musculus]
gi|12835914 **Mass:** 74255 **Score:** 62 **Matches:** 1(1) **Sequences:** 1(1)
unnamed protein product [Mus musculus]
gi|74148166 **Mass:** 74150 **Score:** 62 **Matches:** 1(1) **Sequences:** 1(1)
unnamed protein product [Mus musculus]
gi|74204231 **Mass:** 65451 **Score:** 62 **Matches:** 1(1) **Sequences:** 1(1)
unnamed protein product [Mus musculus]
gi|74219492 **Mass:** 65397 **Score:** 62 **Matches:** 1(1) **Sequences:** 1(1)
unnamed protein product [Mus musculus]
gi|74219697 **Mass:** 65335 **Score:** 62 **Matches:** 1(1) **Sequences:** 1(1)
unnamed protein product [Mus musculus]

gi|74220666 **Mass:** 72427 **Score:** 62 **Matches:** 1(1) **Sequences:** 1(1)
unnamed protein product [Mus musculus]
gi|74220835 **Mass:** 65398 **Score:** 62 **Matches:** 1(1) **Sequences:** 1(1)
unnamed protein product [Mus musculus]
gi|161760667 **Mass:** 65407 **Score:** 62 **Matches:** 1(1) **Sequences:** 1(1)
prelamin-A/C isoform C [Mus musculus]
gi|162287370 **Mass:** 74193 **Score:** 62 **Matches:** 1(1) **Sequences:** 1(1)
prelamin-A/C isoform A [Mus musculus]

---

17. gi|52785 **Mass:** 56409 **Score:** 60 **Matches:** 2(2) **Sequences:** 2(2) **emPAI:** 0.12
unnamed protein product [Mus musculus]

Query	Observed	Mr(expt)	Mr(calc)	Delta	Miss	Score	Expect	Rank	Unique	Peptide
11211	542.0804	1082.1462	1081.5920	0.5541	1	49	0.011	1		K.FASFIDKVR.F
15155	738.6367	1475.2588	1475.7984	-0.5396	1	55	0.0058	4	U	R.FLEQQNKVLETK.W

Proteins matching the same set of peptides:
gi|22164776 **Mass:** 57517 **Score:** 60 **Matches:** 2(2) **Sequences:** 2(2)
keratin, type II cytoskeletal 79 [Mus musculus]
gi|26324736 **Mass:** 58230 **Score:** 60 **Matches:** 2(2) **Sequences:** 2(2)
unnamed protein product [Mus musculus]
gi|29789317 **Mass:** 59704 **Score:** 60 **Matches:** 2(2) **Sequences:** 2(2)
keratin, type II cytoskeletal 75 [Mus musculus]
gi|39850082 **Mass:** 57817 **Score:** 60 **Matches:** 2(2) **Sequences:** 2(2)
Krt4 protein [Mus musculus]
gi|85701680 **Mass:** 62806 **Score:** 60 **Matches:** 2(2) **Sequences:** 2(2)
keratin, type II cytoskeletal 2 oral [Mus musculus]
gi|109735018 **Mass:** 58204 **Score:** 60 **Matches:** 2(2) **Sequences:** 2(2)
RIKEN cDNA 4732456N10 gene [Mus musculus]
gi|133778953 **Mass:** 56248 **Score:** 60 **Matches:** 2(2) **Sequences:** 2(2)
keratin, type II cytoskeletal 4 [Mus musculus]
gi|148672069 **Mass:** 59231 **Score:** 60 **Matches:** 2(2) **Sequences:** 2(2)
cDNA sequence BC031593 [Mus musculus]
gi|148672071 **Mass:** 57541 **Score:** 60 **Matches:** 2(2) **Sequences:** 2(2)
keratin 4, isoform CRA_b [Mus musculus]
gi|148672072 **Mass:** 63519 **Score:** 60 **Matches:** 2(2) **Sequences:** 2(2)
mCG144546 [Mus musculus]
gi|148672089 **Mass:** 62241 **Score:** 60 **Matches:** 2(2) **Sequences:** 2(2)
keratin 75 [Mus musculus]
gi|148672090 **Mass:** 58890 **Score:** 60 **Matches:** 2(2) **Sequences:** 2(2)
RIKEN cDNA 4732456N10 [Mus musculus]
gi|269914154 **Mass:** 58188 **Score:** 60 **Matches:** 2(2) **Sequences:** 2(2)
uncharacterized protein LOC239673 [Mus musculus]

---

18. gi|6677775 **Mass:** 14750 **Score:** 59 **Matches:** 1(1) **Sequences:** 1(1) **emPAI:** 0.23
60S ribosomal protein L22 [Mus musculus]

Query	Observed	Mr(expt)	Mr(calc)	Delta	Miss	Score	Expect	Rank	Unique	Peptide
12933	**622.0134**	**1242.0122**	**1241.6728**	**0.3394**	**0**	**59**	**0.001**	1	U	**K.AGNLGGGVVTIER.S**

---

19. gi|293686 **Mass:** 59414 **Score:** 58 **Matches:** 2(2) **Sequences:** 2(2) **emPAI:** 0.11
epidermal keratin subunit II [Mus musculus]

Query	Observed	Mr(expt)	Mr(calc)	Delta	Miss	Score	Expect	Rank	Unique	Peptide
11211	542.0804	1082.1462	1081.5920	0.5541	1	49	0.011	1		K.FASFIDKVR.F
13133	**634.0348**	**1266.0550**	**1265.6139**	**0.4411**	**0**	**50**	**0.021**	1	U	**R.TDAENEFVTLK.K**

---

20. gi|14917005 **Mass:** 73483 **Score:** 58 **Matches:** 2(2) **Sequences:** 2(2) **emPAI:** 0.09
RecName: Full=Stress-70 protein, mitochondrial; AltName: Full=75 kDa glucose-regulated protein; Short=GRP-75; AltName: Full=Heat shock 70 kDa protein 9; AltName: Full=Mortalin; AltName: Full=Peptide

Query	Observed	Mr(expt)	Mr(calc)	Delta	Miss	Score	Expect	Rank	Unique	Peptide
12939	**622.4032**	**1242.7918**	**1241.6728**	**1.1191**	**0**	**49**	**0.03**	1	U	**K.DAGQISGLNVLR.V**

14049	685.8440	2054.5101	2054.9545	-0.4444	0	55	0.0067	1	U	K.STNGDTFLGGEDFDQALLR.H

Proteins matching the same set of peptides:
gi|74204605 **Mass:** 73446 **Score:** 58 **Matches:** 2(2) **Sequences:** 2(2)
unnamed protein product [Mus musculus]
gi|74205924 **Mass:** 63924 **Score:** 58 **Matches:** 2(2) **Sequences:** 2(2)
unnamed protein product [Mus musculus]
gi|74225724 **Mass:** 73431 **Score:** 58 **Matches:** 2(2) **Sequences:** 2(2)
unnamed protein product [Mus musculus]
gi|162461907 **Mass:** 73416 **Score:** 58 **Matches:** 2(2) **Sequences:** 2(2)
stress-70 protein, mitochondrial [Mus musculus]

---

21. gi|387207 **Mass:** 69698 **Score:** 57 **Matches:** 1(1) **Sequences:** 1(1) **emPAI:** 0.05
heat shock protein [Mus musculus]

Query	Observed	Mr(expt)	Mr(calc)	Delta	Miss	Score	Expect	Rank	Unique	Peptide
13034	627.9152	1253.8158	1252.6088	1.2070	0	57	0.0041	1	U	R.FEELNADLFR.G

Proteins matching the same set of peptides:
gi|31560686 **Mass:** 69599 **Score:** 57 **Matches:** 1(1) **Sequences:** 1(1)
heat shock-related 70 kDa protein 2 [Mus musculus]
gi|74142040 **Mass:** 70841 **Score:** 57 **Matches:** 1(1) **Sequences:** 1(1)
unnamed protein product [Mus musculus]
gi|74179642 **Mass:** 43517 **Score:** 57 **Matches:** 1(1) **Sequences:** 1(1)
unnamed protein product [Mus musculus]
gi|74214304 **Mass:** 56662 **Score:** 57 **Matches:** 1(1) **Sequences:** 1(1)
unnamed protein product [Mus musculus]
gi|77415383 **Mass:** 62117 **Score:** 57 **Matches:** 1(1) **Sequences:** 1(1)
Hspa8 protein, partial [Mus musculus]
gi|215260057 **Mass:** 69681 **Score:** 57 **Matches:** 1(1) **Sequences:** 1(1)
heat shock protein 70-2 [Mus musculus]
gi|309319 **Mass:** 70793 **Score:** 57 **Matches:** 1(1) **Sequences:** 1(1)
heat shock protein 70 cognate [Mus musculus]
gi|13242237 **Mass:** 70827 **Score:** 57 **Matches:** 1(1) **Sequences:** 1(1)
heat shock cognate 71 kDa protein [Rattus norvegicus]
gi|42542422 **Mass:** 70828 **Score:** 57 **Matches:** 1(1) **Sequences:** 1(1)
Heat shock protein 8 [Mus musculus]
gi|63101351 **Mass:** 68736 **Score:** 57 **Matches:** 1(1) **Sequences:** 1(1)
Hspa8 protein [Mus musculus]
gi|74142813 **Mass:** 50433 **Score:** 57 **Matches:** 1(1) **Sequences:** 1(1)
unnamed protein product [Mus musculus]
gi|74143862 **Mass:** 70813 **Score:** 57 **Matches:** 1(1) **Sequences:** 1(1)
unnamed protein product [Mus musculus]
gi|74181586 **Mass:** 70855 **Score:** 57 **Matches:** 1(1) **Sequences:** 1(1)
unnamed protein product [Mus musculus]
gi|74181633 **Mass:** 70841 **Score:** 57 **Matches:** 1(1) **Sequences:** 1(1)
unnamed protein product [Mus musculus]
gi|74184057 **Mass:** 70857 **Score:** 57 **Matches:** 1(1) **Sequences:** 1(1)
unnamed protein product [Mus musculus]
gi|74186087 **Mass:** 70739 **Score:** 57 **Matches:** 1(1) **Sequences:** 1(1)
unnamed protein product [Mus musculus]
gi|74190799 **Mass:** 70813 **Score:** 57 **Matches:** 1(1) **Sequences:** 1(1)
unnamed protein product [Mus musculus]
gi|74191381 **Mass:** 70859 **Score:** 57 **Matches:** 1(1) **Sequences:** 1(1)
unnamed protein product [Mus musculus]
gi|74198858 **Mass:** 70852 **Score:** 57 **Matches:** 1(1) **Sequences:** 1(1)
unnamed protein product [Mus musculus]
gi|74198978 **Mass:** 70903 **Score:** 57 **Matches:** 1(1) **Sequences:** 1(1)
unnamed protein product [Mus musculus]

gi|74208631 **Mass:** 70827 **Score:** 57 **Matches:** 1(1) **Sequences:** 1(1)
unnamed protein product [Mus musculus]
gi|74211333 **Mass:** 68074 **Score:** 57 **Matches:** 1(1) **Sequences:** 1(1)
unnamed protein product [Mus musculus]
gi|74211667 **Mass:** 60764 **Score:** 57 **Matches:** 1(1) **Sequences:** 1(1)
unnamed protein product [Mus musculus]
gi|74214176 **Mass:** 70828 **Score:** 57 **Matches:** 1(1) **Sequences:** 1(1)
unnamed protein product [Mus musculus]
gi|74220416 **Mass:** 70755 **Score:** 57 **Matches:** 1(1) **Sequences:** 1(1)
unnamed protein product [Mus musculus]
gi|74220592 **Mass:** 70648 **Score:** 57 **Matches:** 1(1) **Sequences:** 1(1)
unnamed protein product [Mus musculus]
gi|74225511 **Mass:** 70783 **Score:** 57 **Matches:** 1(1) **Sequences:** 1(1)
unnamed protein product [Mus musculus]
gi|148693577 **Mass:** 35174 **Score:** 57 **Matches:** 1(1) **Sequences:** 1(1)
mCG5074, isoform CRA_a [Mus musculus]
gi|215261181 **Mass:** 42317 **Score:** 57 **Matches:** 1(1) **Sequences:** 1(1)
Chain A, Chaperone Complex

---

22. gi|50797 **Mass:** 50018 **Score:** 55 **Matches:** 2(1) **Sequences:** 1(1) **emPAI:** 0.07
unnamed protein product [Mus musculus]

Query	Observed	Mr(expt)	Mr(calc)	Delta	Miss	Score	Expect	Rank	Unique	Peptide
10649	513.1775	1024.3404	1024.6030	-0.2625	0	52	0.013	1	U	K.IGGIGTVPVGR.V 10648

Proteins matching the same set of peptides:
gi|556301 **Mass:** 50132 **Score:** 55 **Matches:** 2(1) **Sequences:** 1(1)
elongation factor Tu [Mus musculus]
gi|6681273 **Mass:** 50422 **Score:** 55 **Matches:** 2(1) **Sequences:** 1(1)
elongation factor 1-alpha 2 [Mus musculus]
gi|13278382 **Mass:** 50140 **Score:** 55 **Matches:** 2(1) **Sequences:** 1(1)
Eukaryotic translation elongation factor 1 alpha 1 [Mus musculus]
gi|26328693 **Mass:** 50024 **Score:** 55 **Matches:** 2(1) **Sequences:** 1(1)
unnamed protein product [Mus musculus]
gi|26345590 **Mass:** 50086 **Score:** 55 **Matches:** 2(1) **Sequences:** 1(1)
unnamed protein product [Mus musculus]
gi|28460696 **Mass:** 50082 **Score:** 55 **Matches:** 2(1) **Sequences:** 1(1)
elongation factor 1-alpha 1 [Rattus norvegicus]
gi|74195737 **Mass:** 50081 **Score:** 55 **Matches:** 2(1) **Sequences:** 1(1)
unnamed protein product [Mus musculus]
gi|74204203 **Mass:** 50072 **Score:** 55 **Matches:** 2(1) **Sequences:** 1(1)
unnamed protein product [Mus musculus]
gi|74227478 **Mass:** 50034 **Score:** 55 **Matches:** 2(1) **Sequences:** 1(1)
unnamed protein product [Mus musculus]
gi|148694454 **Mass:** 38144 **Score:** 55 **Matches:** 2(1) **Sequences:** 1(1)
mCG15232, isoform CRA_b [Mus musculus]
gi|148694456 **Mass:** 32313 **Score:** 55 **Matches:** 2(1) **Sequences:** 1(1)
mCG15232, isoform CRA_d [Mus musculus]

---

23. gi|6755110 **Mass:** 84869 **Score:** 54 **Matches:** 1(1) **Sequences:** 1(1) **emPAI:** 0.04
procollagen-lysine,2-oxoglutarate 5-dioxygenase 3 precursor [Mus musculus]

Query	Observed	Mr(expt)	Mr(calc)	Delta	Miss	Score	Expect	Rank	Unique	Peptide
14416	700.0134	1398.0123	1397.7150	0.2973	0	54	0.0092	1	U	K.LVGPEEALSAGEAR.D

Proteins matching the same set of peptides:
gi|12850403 **Mass:** 84909 **Score:** 54 **Matches:** 1(1) **Sequences:** 1(1)
unnamed protein product [Mus musculus]

---

24. gi|817996 **Mass:** 40730 **Score:** 53 **Matches:** 1(1) **Sequences:** 1(1) **emPAI:** 0.08
class I (Qa) Q2k antigen [Mus musculus]

Query	Observed	Mr(expt)	Mr(calc)	Delta	Miss	Score	Expect	Rank	Unique	Peptide
11186	1081.2339	1080.2266	1079.4883	0.7383	0	53	0.0038	1	U	R.FDSDAEKPR.Y

Proteins matching the same set of peptides:
gi|148674754 Mass: 40746 Score: 53 Matches: 1(1) Sequences: 1(1)
mCG4996 [Mus musculus]

---

25. gi|19923857 Mass: 49305 Score: 52 Matches: 1(1) Sequences: 1(1) emPAI: 0.07
thymidine phosphorylase [Mus musculus]

Query	Observed	Mr(expt)	Mr(calc)	Delta	Miss	Score	Expect	Rank	Unique	Peptide
15416	754.5466	1507.0786	1507.7419	-0.6634	1	52	0.0098	1	U	K.FGGAAVFPDQEKAR.E

---

26. gi|51452 Mass: 58833 Score: 52 Matches: 1(1) Sequences: 1(1) emPAI: 0.06
unnamed protein product [Mus musculus]

Query	Observed	Mr(expt)	Mr(calc)	Delta	Miss	Score	Expect	Rank	Unique	Peptide
15865	801.0338	1600.0531	1599.9018	0.1513	1	52	0.015	1	U	R.RGVMLAVDAVIAELK.K

Proteins matching the same set of peptides:
gi|148677951 Mass: 17541 Score: 52 Matches: 1(1) Sequences: 1(1)
mCG1033306 [Mus musculus]
gi|148700389 Mass: 49482 Score: 52 Matches: 1(1) Sequences: 1(1)
mCG116284 [Mus musculus]
gi|51455 Mass: 60903 Score: 52 Matches: 1(1) Sequences: 1(1)
heat shock protein 65 [Mus musculus]
gi|26353954 Mass: 60918 Score: 52 Matches: 1(1) Sequences: 1(1)
unnamed protein product [Mus musculus]
gi|76779273 Mass: 59388 Score: 52 Matches: 1(1) Sequences: 1(1)
Hspd1 protein, partial [Mus musculus]
gi|183396771 Mass: 60917 Score: 52 Matches: 1(1) Sequences: 1(1)
60 kDa heat shock protein, mitochondrial [Mus musculus]

---

27. gi|398168 Mass: 70934 Score: 52 Matches: 2(1) Sequences: 2(1) emPAI: 0.09
keratin 2 epidermis [Mus musculus]

Query	Observed	Mr(expt)	Mr(calc)	Delta	Miss	Score	Expect	Rank	Unique	Peptide
11211	542.0804	1082.1462	1081.5920	0.5541	1	49	0.011	1		K.FASFIDKVR.F
13037	628.1775	1254.3404	1253.6001	0.7404	0	44	0.077	1	U	R.GFSSGSAVVSGGSR.R

Proteins matching the same set of peptides:
gi|111308159 Mass: 70880 Score: 52 Matches: 2(1) Sequences: 2(1)
Keratin 2 [Mus musculus]
gi|124487419 Mass: 70880 Score: 52 Matches: 2(1) Sequences: 2(1)
keratin, type II cytoskeletal 2 epidermal [Mus musculus]
gi|148672075 Mass: 71783 Score: 52 Matches: 2(1) Sequences: 2(1)
mCG17605, isoform CRA_a [Mus musculus]
gi|148672076 Mass: 72140 Score: 52 Matches: 2(1) Sequences: 2(1)
mCG17605, isoform CRA_b [Mus musculus]

---

28. gi|15277319 Mass: 34752 Score: 51 Matches: 1(1) Sequences: 1(1) emPAI: 0.10
leucine zipper transcription factor-like protein 1 [Mus musculus]

Query	Observed	Mr(expt)	Mr(calc)	Delta	Miss	Score	Expect	Rank	Unique	Peptide
12441	595.5423	1189.0701	1189.5939	-0.5238	1	51	0.0072	1	U	K.TLNDKTENQK.S

Proteins matching the same set of peptides:
gi|26325604 Mass: 31688 Score: 51 Matches: 1(1) Sequences: 1(1)
unnamed protein product [Mus musculus]
gi|26326047 Mass: 34639 Score: 51 Matches: 1(1) Sequences: 1(1)
unnamed protein product [Mus musculus]

gi|26334199 **Mass:** 34694 **Score:** 51 **Matches:** 1(1) **Sequences:** 1(1)
unnamed protein product [Mus musculus]
gi|74181149 **Mass:** 33628 **Score:** 51 **Matches:** 1(1) **Sequences:** 1(1)
unnamed protein product [Mus musculus]

---

29. gi|200765 **Mass:** 90163 **Score:** 51 **Matches:** 1(1) **Sequences:** 1(1) **emPAI:** 0.04
ribonucleotide reductase subunit M1 [Mus musculus]

Query	Observed	Mr(expt)	Mr(calc)	Delta	Miss	Score	Expect	Rank	Unique	Peptide
15601	768.6637	1535.3128	1534.7667	0.5461	0	51	0.016	1	U	R.YPFESPEAQLLNK.Q

Proteins matching the same set of peptides:
gi|31982026 **Mass:** 90153 **Score:** 51 **Matches:** 1(1) **Sequences:** 1(1)
ribonucleoside-diphosphate reductase large subunit [Mus musculus]
gi|42490975 **Mass:** 90093 **Score:** 51 **Matches:** 1(1) **Sequences:** 1(1)
Ribonucleotide reductase M1 [Mus musculus]
gi|116283230 **Mass:** 86782 **Score:** 51 **Matches:** 1(1) **Sequences:** 1(1)
Rrm1 protein [Mus musculus]

---

30. gi|191765 **Mass:** 47195 **Score:** 51 **Matches:** 1(1) **Sequences:** 1(1) **emPAI:** 0.07
alpha-fetoprotein, partial [Mus musculus]

Query	Observed	Mr(expt)	Mr(calc)	Delta	Miss	Score	Expect	Rank	Unique	Peptide
15188	740.1958	1478.3770	1478.7881	-0.4111	0	51	0.015	1	U	K.LGEYGFQNAILVR.Y

Proteins matching the same set of peptides:
gi|74137565 **Mass:** 68688 **Score:** 51 **Matches:** 1(1) **Sequences:** 1(1)
unnamed protein product [Mus musculus]
gi|26340966 **Mass:** 68678 **Score:** 51 **Matches:** 1(1) **Sequences:** 1(1)
unnamed protein product [Mus musculus]
gi|26341396 **Mass:** 64961 **Score:** 51 **Matches:** 1(1) **Sequences:** 1(1)
unnamed protein product [Mus musculus]
gi|163310765 **Mass:** 68648 **Score:** 51 **Matches:** 1(1) **Sequences:** 1(1)
serum albumin precursor [Mus musculus]

---

31. gi|18480346 **Score:** 50 **Matches:** 1(1) **Sequences:** 1(1) **emPAI:** 0.09
olfactory receptor MOR175-2 [Mus musculus]

Query	Observed	Mr(expt)	Mr(calc)	Delta	Miss	Score	Expect	Rank	Unique	Peptide
6979	366.0798	730.1451	731.3562	-1.2111	0	50	0.013	1	U	K.INSADGR.R

Proteins matching the same set of peptides:
gi|121583707 **Score:** 50 **Matches:** 1(1) **Sequences:** 1(1)

---

32. gi|226005 **Mass:** 32732 **Score:** 50 **Matches:** 1(1) **Sequences:** 1(1) **emPAI:** 0.10
protein 40kD

Query	Observed	Mr(expt)	Mr(calc)	Delta	Miss	Score	Expect	Rank	Unique	Peptide
12564	602.6827	1203.3509	1202.6408	0.7102	0	50	0.025	1	U	K.FAAATGATPIAGR.F

Proteins matching the same set of peptides:
gi|293694 **Mass:** 32698 **Score:** 50 **Matches:** 1(1) **Sequences:** 1(1)
laminin receptor [Mus musculus]
gi|12846904 **Mass:** 32840 **Score:** 50 **Matches:** 1(1) **Sequences:** 1(1)
unnamed protein product [Mus musculus]
gi|26344606 **Mass:** 10928 **Score:** 50 **Matches:** 1(1) **Sequences:** 1(1)
unnamed protein product [Mus musculus]
gi|26350123 **Mass:** 32893 **Score:** 50 **Matches:** 1(1) **Sequences:** 1(1)
unnamed protein product [Mus musculus]
gi|62024907 **Mass:** 32821 **Score:** 50 **Matches:** 1(1) **Sequences:** 1(1)
Ribosomal protein SA [Mus musculus]

gi|82905443 **Mass:** 32936 **Score:** 50 **Matches:** 1(1) **Sequences:** 1(1)
PREDICTED: 40S ribosomal protein SA-like isoform 1 [Mus musculus]
gi|148237408 **Mass:** 32817 **Score:** 50 **Matches:** 1(1) **Sequences:** 1(1)
uncharacterized protein LOC100037080 [Xenopus laevis]
gi|148680000 **Mass:** 32817 **Score:** 50 **Matches:** 1(1) **Sequences:** 1(1)
mCG2650 [Mus musculus]
gi|148691799 **Mass:** 32640 **Score:** 50 **Matches:** 1(1) **Sequences:** 1(1)
mCG68071 [Mus musculus]
gi|171948782 **Mass:** 32829 **Score:** 50 **Matches:** 1(1) **Sequences:** 1(1)
laminin receptor [Mus musculus]

---

33. gi|193645 **Mass:** 18932 **Score:** 50 **Matches:** 1(1) **Sequences:** 1(1) **emPAI:** 0.18
glucose-regulated protein 78, partial [Mus musculus]

Query	Observed	Mr(expt)	Mr(calc)	Delta	Miss	Score	Expect	Rank	Unique	Peptide
14399	699.2704	1396.5262	1396.7813	-0.2551	0	50	0.025	1	U	K.ELEEIVQPIISK.L

Proteins matching the same set of peptides:
gi|387113 **Mass:** 16164 **Score:** 50 **Matches:** 1(1) **Sequences:** 1(1)
immunoglobulin heavy chain binding protein, partial [Mus musculus]
gi|1304157 **Mass:** 72412 **Score:** 50 **Matches:** 1(1) **Sequences:** 1(1)
78 kDa glucose-regulated protein [Mus musculus]
gi|2598562 **Mass:** 72433 **Score:** 50 **Matches:** 1(1) **Sequences:** 1(1)
BiP [Mus musculus]
gi|12835845 **Mass:** 72378 **Score:** 50 **Matches:** 1(1) **Sequences:** 1(1)
unnamed protein product [Mus musculus]
gi|74188814 **Mass:** 72305 **Score:** 50 **Matches:** 1(1) **Sequences:** 1(1)
unnamed protein product [Mus musculus]
gi|74198293 **Mass:** 72419 **Score:** 50 **Matches:** 1(1) **Sequences:** 1(1)
unnamed protein product [Mus musculus]
gi|74198974 **Mass:** 72361 **Score:** 50 **Matches:** 1(1) **Sequences:** 1(1)
unnamed protein product [Mus musculus]
gi|74207492 **Mass:** 72301 **Score:** 50 **Matches:** 1(1) **Sequences:** 1(1)
unnamed protein product [Mus musculus]
gi|74225394 **Mass:** 72337 **Score:** 50 **Matches:** 1(1) **Sequences:** 1(1)
unnamed protein product [Mus musculus]
gi|148676670 **Mass:** 56257 **Score:** 50 **Matches:** 1(1) **Sequences:** 1(1)
heat shock 70kD protein 5 (glucose-regulated protein), isoform CRA_b [Mus musculus]
gi|254540166 **Mass:** 72377 **Score:** 50 **Matches:** 1(1) **Sequences:** 1(1)
78 kDa glucose-regulated protein precursor [Mus musculus]

---

34. gi|6996913 **Mass:** 38652 **Score:** 50 **Matches:** 1(1) **Sequences:** 1(1) **emPAI:** 0.09
annexin A2 [Mus musculus]

Query	Observed	Mr(expt)	Mr(calc)	Delta	Miss	Score	Expect	Rank	Unique	Peptide
12728	611.6130	1221.2114	1221.5877	-0.3763	0	50	0.023	1	U	K.TPAQYDASELK.A

Proteins matching the same set of peptides:
gi|12849385 **Mass:** 38585 **Score:** 50 **Matches:** 1(1) **Sequences:** 1(1)
unnamed protein product [Mus musculus]
gi|74151782 **Mass:** 38636 **Score:** 50 **Matches:** 1(1) **Sequences:** 1(1)
unnamed protein product [Mus musculus]

---

35. gi|116283284 **Mass:** 72885 **Score:** 49 **Matches:** 1(1) **Sequences:** 1(1) **emPAI:** 0.05
Por protein [Mus musculus]

Query	Observed	Mr(expt)	Mr(calc)	Delta	Miss	Score	Expect	Rank	Unique	Peptide
13383	649.1569	1296.2993	1296.6363	-0.3370	0	49	0.023	1	U	R.WAAAPAGWAAPGR.R

---

36. gi|12805465 **Mass:** 54890 **Score:** 48 **Matches:** 1(1) **Sequences:** 1(1) **emPAI:** 0.06
Dnajc10 protein, partial [Mus musculus]

Query	Observed	Mr(expt)	Mr(calc)	Delta	Miss	Score	Expect	Rank	Unique	Peptide
13357	647.5936	1293.1727	1292.6976	0.4751	0	48	0.033	1	U	K.SSVLFLNSLDAK.E

Proteins matching the same set of peptides:
gi|12835910 **Mass:** 90547 **Score:** 48 **Matches:** 1(1) **Sequences:** 1(1)
unnamed protein product [Mus musculus]
gi|23270977 **Mass:** 90441 **Score:** 48 **Matches:** 1(1) **Sequences:** 1(1)
DnaJ (Hsp40) homolog, subfamily C, member 10 [Mus musculus]
gi|25140581 **Mass:** 90768 **Score:** 48 **Matches:** 1(1) **Sequences:** 1(1)
ER-resident protein ERdj5 [Mus musculus]
gi|33340135 **Mass:** 90521 **Score:** 48 **Matches:** 1(1) **Sequences:** 1(1)
endoplasmic reticulum DnaJ-PDI fusion protein 1 precursor [Mus musculus]
gi|119508443 **Mass:** 90525 **Score:** 48 **Matches:** 1(1) **Sequences:** 1(1)
dnaJ homolog subfamily C member 10 precursor [Mus musculus]
gi|148695313 **Mass:** 80660 **Score:** 48 **Matches:** 1(1) **Sequences:** 1(1)
mCG12166 [Mus musculus]
gi|329665912 **Mass:** 88825 **Score:** 48 **Matches:** 1(1) **Sequences:** 1(1)
Chain A, Crystal Structure Of Full-Length Erdj5

---

37. gi|148678782 **Mass:** 9063 **Score:** 48 **Matches:** 1(1) **Sequences:** 1(1) **emPAI:** 0.38
mCG1027198, isoform CRA_a [Mus musculus]

Query	Observed	Mr(expt)	Mr(calc)	Delta	Miss	Score	Expect	Rank	Unique	Peptide
11795	568.0061	1133.9976	1134.5451	-0.5475	1	48	0.015	1	U	K.ERMESSGAIR.I

---

38. gi|16716569 **Mass:** 26118 **Score:** 48 **Matches:** 1(1) **Sequences:** 1(1) **emPAI:** 0.13
protease, serine, 1 precursor [Mus musculus]

Query	Observed	Mr(expt)	Mr(calc)	Delta	Miss	Score	Expect	Rank	Unique	Peptide
15127	737.7551	2210.2436	2210.0967	0.1468	0	48	0.04	1	U	R.LGEHNINVLEGNEQFIDAAK.I

---

39. gi|19924302 **Mass:** 77934 **Score:** 47 **Matches:** 1(1) **Sequences:** 1(1) **emPAI:** 0.04
ankyrin repeat domain-containing protein 6 [Mus musculus]

Query	Observed	Mr(expt)	Mr(calc)	Delta	Miss	Score	Expect	Rank	Unique	Peptide
12288	588.4644	1174.9142	1174.5951	0.3192	1	47	0.02	1	U	K.VMQAPIKGCR.C

Proteins matching the same set of peptides:
gi|50510725 **Mass:** 78081 **Score:** 47 **Matches:** 1(1) **Sequences:** 1(1)
mKIAA0957 protein [Mus musculus]

---

40. gi|148680049 **Mass:** 5496 **Score:** 47 **Matches:** 1(1) **Sequences:** 1(1) **emPAI:** 0.65
mCG147414 [Mus musculus]

Query	Observed	Mr(expt)	Mr(calc)	Delta	Miss	Score	Expect	Rank	Unique	Peptide
15915	811.5035	1620.9925	1619.8770	1.1155	0	47	0.046	1	U	M.YPLAVGATSGLVTLDK.Q

---

41. gi|26337475 **Mass:** 33168 **Score:** 47 **Matches:** 1(1) **Sequences:** 1(1) **emPAI:** 0.10
unnamed protein product [Mus musculus]

Query	Observed	Mr(expt)	Mr(calc)	Delta	Miss	Score	Expect	Rank	Unique	Peptide
7984	789.6132	788.6059	789.3617	-0.7558	0	47	0.033	1	U	R.SGSSPSPR.A

Proteins matching the same set of peptides:
gi|309095 **Mass:** 135080 **Score:** 47 **Matches:** 1(1) **Sequences:** 1(1)
AE3 protein [Mus musculus]
gi|51328345 **Mass:** 136564 **Score:** 47 **Matches:** 1(1) **Sequences:** 1(1)
Slc4a3 protein [Mus musculus]
gi|165377246 **Mass:** 135288 **Score:** 47 **Matches:** 1(1) **Sequences:** 1(1)
anion exchange protein 3 [Mus musculus]

---

42. gi|199938 **Mass:** 65935 **Score:** 46 **Matches:** 1(1) **Sequences:** 1(1) **emPAI:** 0.05
tumor-specific myb protein [Mus musculus]

Query	Observed	Mr(expt)	Mr(calc)	Delta	Miss	Score	Expect	Rank	Unique	Peptide
11505	555.3855	1108.7564	1108.4715	0.2849	0	46	0.025	1	U	-.MGAPLNCPMK.S

Proteins matching the same set of peptides:
gi|199929 **Mass:** 79128 **Score:** 46 **Matches:** 1(1) **Sequences:** 1(1)
myb protein [Mus musculus]

---

43. gi|22094097 **Mass:** 56233 **Score:** 46 **Matches:** 1(1) **Sequences:** 1(1) **emPAI:** 0.06
ganglioside-induced differentiation-associated protein 2 [Mus musculus]

Query	Observed	Mr(expt)	Mr(calc)	Delta	Miss	Score	Expect	Rank	Unique	Peptide
7268	747.2545	746.2473	747.4028	-1.1555	0	46	0.04	1	U	K.GFNLAAR.F

Proteins matching the same set of peptides:
gi|26350139 **Mass:** 60978 **Score:** 46 **Matches:** 1(1) **Sequences:** 1(1)
unnamed protein product [Mus musculus]
gi|148675693 **Mass:** 60979 **Score:** 46 **Matches:** 1(1) **Sequences:** 1(1)
ganglioside-induced differentiation-associated-protein 2, isoform CRA_a [Mus musculus]

---

44. gi|198683 **Mass:** 35858 **Score:** 45 **Matches:** 1(1) **Sequences:** 1(1) **emPAI:** 0.09
unnamed protein product [Mus musculus]

Query	Observed	Mr(expt)	Mr(calc)	Delta	Miss	Score	Expect	Rank	Unique	Peptide
9577	457.5262	913.0378	912.5756	0.4622	0	45	0.024	1	U	K.LVIITAGAR.M

Proteins matching the same set of peptides:
gi|6754524 **Mass:** 36475 **Score:** 45 **Matches:** 1(1) **Sequences:** 1(1)
L-lactate dehydrogenase A chain isoform 1 [Mus musculus]
gi|7305229 **Mass:** 35889 **Score:** 45 **Matches:** 1(1) **Sequences:** 1(1)
L-lactate dehydrogenase C chain [Mus musculus]
gi|13529599 **Mass:** 34481 **Score:** 45 **Matches:** 1(1) **Sequences:** 1(1)
Ldha protein, partial [Mus musculus]
gi|44890285 **Mass:** 11164 **Score:** 45 **Matches:** 1(1) **Sequences:** 1(1)
Ldha protein [Mus musculus]
gi|74204388 **Mass:** 36461 **Score:** 45 **Matches:** 1(1) **Sequences:** 1(1)
unnamed protein product [Mus musculus]
gi|74208131 **Mass:** 36242 **Score:** 45 **Matches:** 1(1) **Sequences:** 1(1)
unnamed protein product [Mus musculus]
gi|74212250 **Mass:** 36476 **Score:** 45 **Matches:** 1(1) **Sequences:** 1(1)
unnamed protein product [Mus musculus]
gi|74217959 **Mass:** 36476 **Score:** 45 **Matches:** 1(1) **Sequences:** 1(1)
unnamed protein product [Mus musculus]
gi|157835307 **Mass:** 35801 **Score:** 45 **Matches:** 1(1) **Sequences:** 1(1)
Chain A, Characterization Of The Antigenic Sites On The Refined 3- Angstroms Resolution Structure Of Mouse Testicular Lactate Dehydrogenase C4
gi|257743039 **Mass:** 39733 **Score:** 45 **Matches:** 1(1) **Sequences:** 1(1)
L-lactate dehydrogenase A chain isoform 2 [Mus musculus]

---

45. gi|26327761 **Mass:** 90833 **Score:** 45 **Matches:** 1(1) **Sequences:** 1(1) **emPAI:** 0.04
unnamed protein product [Mus musculus]

Query	Observed	Mr(expt)	Mr(calc)	Delta	Miss	Score	Expect	Rank	Unique	Peptide
11852	571.0167	1140.0188	1139.6550	0.3638	0	45	0.026	1	U	K.LALPADSVNIK.I

Proteins matching the same set of peptides:
gi|26332879 **Mass:** 40136 **Score:** 45 **Matches:** 1(1) **Sequences:** 1(1)
unnamed protein product [Mus musculus]
gi|38257756 **Mass:** 90916 **Score:** 45 **Matches:** 1(1) **Sequences:** 1(1)
RecName: Full=Cullin-5; Short=CUL-5

gi|239051067 **Mass:** 98979 **Score:** 45 **Matches:** 1(1) **Sequences:** 1(1)
cullin-5 isoform 1 [Mus musculus]
gi|239051082 **Mass:** 95790 **Score:** 45 **Matches:** 1(1) **Sequences:** 1(1)
cullin-5 isoform 2 [Mus musculus]

---

46. gi|51092301 **Mass:** 60191 **Score:** 44 **Matches:** 1(0) **Sequences:** 1(0) **emPAI:** 0.05
type II keratin Kb14 [Mus musculus]

Query	Observed	Mr(expt)	Mr(calc)	Delta	Miss	Score	Expect	Rank	Unique	Peptide
13359	647.7845	1940.3318	1940.9996	-0.6678	1	44	0.072	1	U	R.FASFIDKVQFLEQQNK.V

Proteins matching the same set of peptides:
gi|148672085 **Mass:** 38119 **Score:** 44 **Matches:** 1(0) **Sequences:** 1(0)
mCG144996 [Mus musculus]

---

47. gi|74208129 **Mass:** 95473 **Score:** 44 **Matches:** 1(1) **Sequences:** 1(1) **emPAI:** 0.03
unnamed protein product [Mus musculus]

Query	Observed	Mr(expt)	Mr(calc)	Delta	Miss	Score	Expect	Rank	Unique	Peptide
11982	577.0237	1152.0328	1152.4758	-0.4430	0	44	0.028	1	U	R.SPSYPPPGCGK.S

Proteins matching the same set of peptides:
gi|74211952 **Mass:** 35351 **Score:** 44 **Matches:** 1(1) **Sequences:** 1(1)
unnamed protein product [Mus musculus]
gi|116248568 **Mass:** 95499 **Score:** 44 **Matches:** 1(1) **Sequences:** 1(1)
RecName: Full=Poly [ADP-ribose] polymerase 8; Short=PARP-8
gi|124486598 **Mass:** 99423 **Score:** 44 **Matches:** 1(1) **Sequences:** 1(1)
poly [ADP-ribose] polymerase 8 [Mus musculus]
gi|148686417 **Mass:** 92421 **Score:** 44 **Matches:** 1(1) **Sequences:** 1(1)
mCG119138 [Mus musculus]

---

48. gi|148685252 **Mass:** 396271 **Score:** 44 **Matches:** 1(1) **Sequences:** 1(1) **emPAI:** 0.01
mCG142044 [Mus musculus]

Query	Observed	Mr(expt)	Mr(calc)	Delta	Miss	Score	Expect	Rank	Unique	Peptide
11130	537.3717	1072.7289	1071.5924	1.1365	1	44	0.051	1	U	R.AELKLVEDR.L

Proteins matching the same set of peptides:
gi|182637561 **Mass:** 467476 **Score:** 44 **Matches:** 1(1) **Sequences:** 1(1)
RecName: Full=Dynein heavy chain 3, axonemal; AltName: Full=Axonemal beta dynein heavy chain 3; AltName: Full=Ciliary dynein heavy chain 3

---

49. gi|148671989 **Score:** 44 **Matches:** 1(1) **Sequences:** 1(1) **emPAI:** 0.24
mCG1027581 [Mus musculus]

Query	Observed	Mr(expt)	Mr(calc)	Delta	Miss	Score	Expect	Rank	Unique	Peptide
7739	387.8668	773.7190	774.4236	-0.7045	0	44	0.041	1	U	K.ISSPSLR.I

---

50. gi|26349347 **Mass:** 135092 **Score:** 44 **Matches:** 1(1) **Sequences:** 1(1) **emPAI:** 0.02
unnamed protein product [Mus musculus]

Query	Observed	Mr(expt)	Mr(calc)	Delta	Miss	Score	Expect	Rank	Unique	Peptide
13447	652.5267	1303.0388	1302.6238	0.4150	0	44	0.039	1	U	-.MVEAAPAGSGPLR.R

Proteins matching the same set of peptides:
gi|148689974 **Mass:** 136815 **Score:** 44 **Matches:** 1(1) **Sequences:** 1(1)
RIKEN cDNA 2310057J16, isoform CRA_a [Mus musculus]
gi|255069774 **Mass:** 135191 **Score:** 44 **Matches:** 1(1) **Sequences:** 1(1)
calmodulin-regulated spectrin-associated protein 3 isoform 1 [Mus musculus]
gi|255069776 **Mass:** 135091 **Score:** 44 **Matches:** 1(1) **Sequences:** 1(1)
calmodulin-regulated spectrin-associated protein 3 isoform 2 [Mus musculus]

---

51. gi|6755893 **Mass:** 26257 **Score:** 43 **Matches:** 1(0) **Sequences:** 1(0) **emPAI:** 0.13

trypsin 4 precursor [Mus musculus]

Query	Observed	Mr(expt)	Mr(calc)	Delta	Miss	Score	Expect	Rank	Unique	Peptide
15139	738.1550	2211.4433	2211.0920	0.3513	0	43	0.079	1	U	R.LGEHNINVLEGNEQFVNSAK.I

Proteins matching the same set of peptides:

gi|51010909 **Mass:** 26260 **Score:** 43 **Matches:** 1(0) **Sequences:** 1(0)
trypsin 5 precursor [Mus musculus]

gi|74203392 **Mass:** 27146 **Score:** 43 **Matches:** 1(0) **Sequences:** 1(0)
unnamed protein product [Mus musculus]

---

52. gi|6754976 **Mass:** 22162 **Score:** 43 **Matches:** 1(1) **Sequences:** 1(1) **emPAI:** 0.15
peroxiredoxin-1 [Mus musculus]

Query	Observed	Mr(expt)	Mr(calc)	Delta	Miss	Score	Expect	Rank	Unique	Peptide
11476	554.4471	1106.8797	1106.5972	0.2826	0	43	0.039	1	U	R.TIAQDYGVLK.A

Proteins matching the same set of peptides:

gi|12846314 **Mass:** 22222 **Score:** 43 **Matches:** 1(1) **Sequences:** 1(1)
unnamed protein product [Mus musculus]

gi|74198890 **Mass:** 22130 **Score:** 43 **Matches:** 1(1) **Sequences:** 1(1)
unnamed protein product [Mus musculus]

gi|148665674 **Mass:** 22248 **Score:** 43 **Matches:** 1(1) **Sequences:** 1(1)
mCG127770 [Mus musculus]

Mascot: http://www.matrixscience.com/

Mascot Search Results

PFHR9

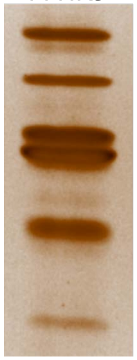

2

User :
Email :
Search title :
MS data file : ZIEN-29Nov2017_2m.mgf
Database : NCBInr 20120419 (17893860 sequences; 6141683785 residues)
Taxonomy : Mus musculus (house mouse) (138831 sequences)
Timestamp : 30 Nov 2017 at 20:24:37 GMT
Enzyme : Trypsin
Variable modifications : Carbamidomethyl (C),Oxidation (P),Oxidation (M)
Mass values : Monoisotopic
Protein Mass : Unrestricted
Peptide Mass Tolerance : ± 1.25 Da
Fragment Mass Tolerance: ± 1.001 Da
Max Missed Cleavages : 1
Instrument type : Default
Number of queries : 16468
Protein hits : gi|2598562 BiP [Mus musculus]
gi|14917005 RecName: Full=Stress-70 protein, mitochondrial; AltName: Full=75 kDa glucose-regulated protein; Short=GRP-75; AltName: Full=Heat shock 70 kDa protein 9; AltName: Full=
gi|74211667 unnamed protein product [Mus musculus]
gi|2498741 RecName: Full=Prolyl 4-hydroxylase subunit alpha-2; Short=4-PH alpha-2; AltName: Full=Procollagen-proline,2-oxoglutarate-4-dioxygenase subunit alpha-2; Flags: Precursc
gi|387397 epidermal keratin subunit I, partial [Mus musculus]
gi|46485130 TPA_exp: keratin Kb40 [Mus musculus]
gi|85701680 keratin, type II cytoskeletal 2 oral [Mus musculus]
gi|387399 epidermal keratin type I, partial [Mus musculus]
gi|16303309 type II keratin 5 [Mus musculus]
gi|26349293 unnamed protein product [Mus musculus]
gi|6753620 ATP-dependent RNA helicase DDX3X [Mus musculus]
gi|47523977 keratin, type II cytoskeletal 72 [Mus musculus]
gi|16716569 protease, serine, 1 precursor [Mus musculus]
gi|1389682 plakoglobin, partial [Mus musculus]
gi|19354359 Ranbp10 protein [Mus musculus]
gi|52789 unnamed protein product [Mus musculus]
gi|54777 unnamed protein product [Mus musculus]
gi|20070408 glycine dehydrogenase [decarboxylating], mitochondrial precursor [Mus musculus]
gi|3642689 immunoglobulin heavy chain-binding protein, partial [Mus musculus]
gi|26344548 unnamed protein product [Mus musculus]
gi|836898 prolyl 4-hydroxylase alpha(I)-subunit, partial [Mus musculus]
gi|4159806 type II keratin subunit protein [Mus musculus]
gi|37935731 ATP-binding cassette transporter sub-family A member 16 [Mus musculus]
gi|21595163 Glycosyltransferase 25 domain containing 1 [Mus musculus]
gi|12847276 unnamed protein product [Mus musculus]
gi|74191500 unnamed protein product [Mus musculus]
gi|81875970 RecName: Full=Alkyldihydroxyacetonephosphate synthase, peroxisomal; Short=Alkyl-DHAP synthase; AltName: Full=Alkylglycerone-phosphate synthase; Flags: Precursor
gi|7513694 IgG Fc binding protein - mouse (fragment)
gi|148664969 RIKEN cDNA 2900011O08, isoform CRA_a [Mus musculus]
gi|12855410 unnamed protein product [Mus musculus]

Select Summary Report

Format As | Select Summary (protein hits) | Help

Significance threshold p< 0.05 Max. number of hits AUTO

Standard scoring ○ MudPIT scoring ◉ Ions score or expect cut-off 43 Show sub-sets 0

Show pop-ups ◉ Suppress pop-ups ○ Require bold red ☐

Re-Search ◉ All queries ○ Unassigned ○ Below homology threshold ○ Below identity threshold

1. gi|2598562 Mass: 72433 Score: 183 Matches: 10(8) Sequences: 6(6) emPAI: 0.30
BiP [Mus musculus]

Query	Observed	Mr(expt)	Mr(calc)	Delta	Miss	Score	Expect	Rank	Unique	Peptide
7641	758.4543	757.4470	757.4334	0.0136	0	51	0.011	1	U	R.NTVVPTK.K
10764	499.1691	996.3237	996.5101	-0.1863	0	49	0.012	1	U	R.ALSSQHQAR.I
13124	609.0809	1216.1473	1216.6234	-0.4761	0	52	0.011	1	U	K.DAGTIAGLNVMR.I 13134

13280	615.1929	1228.3712	1227.6207	0.7504	0	66	0.00052	1		R.VEIIANDQGNR.I
14833	699.2899	1396.5653	1396.7813	-0.2160	0	60	0.0025	1	U	K.ELEEIVQPIISK.L 14827
15459	731.0733	1460.1320	1459.7518	0.3802	0	79	2.8e-005	1	U	K.SDIDEIVLVGGSTR.I 15458 15465

Proteins matching the same set of peptides:
gi|12835845 **Mass:** 72378 **Score:** 183 **Matches:** 10(8) **Sequences:** 6(6)
unnamed protein product [Mus musculus]
gi|74188814 **Mass:** 72305 **Score:** 183 **Matches:** 10(8) **Sequences:** 6(6)
unnamed protein product [Mus musculus]
gi|74198293 **Mass:** 72419 **Score:** 183 **Matches:** 10(8) **Sequences:** 6(6)
unnamed protein product [Mus musculus]
gi|74198974 **Mass:** 72361 **Score:** 183 **Matches:** 10(8) **Sequences:** 6(6)
unnamed protein product [Mus musculus]
gi|74207492 **Mass:** 72301 **Score:** 183 **Matches:** 10(8) **Sequences:** 6(6)
unnamed protein product [Mus musculus]
gi|74225394 **Mass:** 72337 **Score:** 183 **Matches:** 10(8) **Sequences:** 6(6)
unnamed protein product [Mus musculus]
gi|254540166 **Mass:** 72377 **Score:** 183 **Matches:** 10(8) **Sequences:** 6(6)
78 kDa glucose-regulated protein precursor [Mus musculus]

---

2. gi|14917005 **Mass:** 73483 **Score:** 174 **Matches:** 10(10) **Sequences:** 7(7) **emPAI:** 0.36
RecName: Full=Stress-70 protein, mitochondrial; AltName: Full=75 kDa glucose-regulated protein; Short=GRP-75; AltName: Full=Heat shock 70 kDa protein 9; AltName: Full=Mortalin; AltName: Full=Peptide

Query	Observed	Mr(expt)	Mr(calc)	Delta	Miss	Score	Expect	Rank	Unique	Peptide
10606	978.6167	977.6095	977.4488	0.1607	0	54	0.0056	1	U	K.AMQDAEVSK.S
13304	616.1958	1230.3770	1230.6568	-0.2797	0	48	0.034	1	U	R.QAASSLQQASLK.L
13836	645.6029	1289.1913	1289.6728	-0.4816	0	56	0.0048	1	U	K.VQQTVQDLFGR.A 13846 13847
14194	667.1742	1332.3338	1332.7289	-0.3951	0	56	0.0025	1	U	R.AQFEGIVTDLIK.R
15362	726.0390	1450.0635	1449.7100	0.3535	0	60	0.00088	1	U	R.TTPSVVAFTADGER.L 15364
16185	786.0322	1570.0499	1568.8272	1.2227	0	46	0.053	1	U	K.LYSPSQIGAFVLMK.M
15239	720.0499	2157.1280	2157.0372	0.0908	1	61	0.0018	1	U	K.ERVEAVNMAEGIIHDTETK.M

Proteins matching the same set of peptides:
gi|74204605 **Mass:** 73446 **Score:** 174 **Matches:** 10(10) **Sequences:** 7(7)
unnamed protein product [Mus musculus]
gi|74225724 **Mass:** 73431 **Score:** 174 **Matches:** 10(10) **Sequences:** 7(7)
unnamed protein product [Mus musculus]
gi|162461907 **Mass:** 73416 **Score:** 174 **Matches:** 10(10) **Sequences:** 7(7)
stress-70 protein, mitochondrial [Mus musculus]

---

3. gi|74211667 **Mass:** 60764 **Score:** 169 **Matches:** 9(8) **Sequences:** 9(8) **emPAI:** 0.61
unnamed protein product [Mus musculus]

Query	Observed	Mr(expt)	Mr(calc)	Delta	Miss	Score	Expect	Rank	Unique	Peptide
7734	765.3373	764.3300	764.4068	-0.0768	0	48	0.016	1	U	K.VQVEYK.G
13280	615.1929	1228.3712	1227.6207	0.7504	0	66	0.00052	1		K.VEIIANDQGNR.T
13478	627.0481	1252.0816	1252.6088	-0.5271	0	47	0.039	1	U	R.FEELNADLFR.G
13632	634.9739	1267.9332	1267.6482	0.2850	1	54	0.0091	1	U	K.MKEIAEAYLGK.T
13950	652.5933	1303.1720	1302.5914	0.5806	0	43	0.094	1	U	K.NSLESYAFNMK.A
15105	713.9242	1425.8338	1425.6558	0.1780	1	66	0.00051	1	U	R.RFDDAVVQSDMK.H
15642	741.6528	1481.2910	1480.7998	0.4912	0	70	0.00019	1	U	K.SQIHDIVLVGGSTR.I
16318	809.0371	1616.0595	1615.7804	0.2792	0	63	0.0011	1	U	K.SFYPEEVSSMVLTK.M
15925	759.1199	2274.3378	2275.1332	-0.7954	0	49	0.023	1	U	K.SINPDEAVAYGAAVQAAILSGDK.S

Proteins matching the same set of peptides:
gi|309319 **Mass:** 70793 **Score:** 169 **Matches:** 9(8) **Sequences:** 9(8)
heat shock protein 70 cognate [Mus musculus]
gi|13242237 **Mass:** 70827 **Score:** 169 **Matches:** 9(8) **Sequences:** 9(8)
heat shock cognate 71 kDa protein [Rattus norvegicus]
gi|42542422 **Mass:** 70828 **Score:** 169 **Matches:** 9(8) **Sequences:** 9(8)

Heat shock protein 8 [Mus musculus]
gi|63101351 **Mass:** 68736 **Score:** 169 **Matches:** 9(8) **Sequences:** 9(8)
Hspa8 protein [Mus musculus]
gi|74143862 **Mass:** 70813 **Score:** 169 **Matches:** 9(8) **Sequences:** 9(8)
unnamed protein product [Mus musculus]
gi|74181586 **Mass:** 70855 **Score:** 169 **Matches:** 9(8) **Sequences:** 9(8)
unnamed protein product [Mus musculus]
gi|74184057 **Mass:** 70857 **Score:** 169 **Matches:** 9(8) **Sequences:** 9(8)
unnamed protein product [Mus musculus]
gi|74186087 **Mass:** 70739 **Score:** 169 **Matches:** 9(8) **Sequences:** 9(8)
unnamed protein product [Mus musculus]
gi|74190799 **Mass:** 70813 **Score:** 169 **Matches:** 9(8) **Sequences:** 9(8)
unnamed protein product [Mus musculus]
gi|74191381 **Mass:** 70859 **Score:** 169 **Matches:** 9(8) **Sequences:** 9(8)
unnamed protein product [Mus musculus]
gi|74198858 **Mass:** 70852 **Score:** 169 **Matches:** 9(8) **Sequences:** 9(8)
unnamed protein product [Mus musculus]
gi|74198978 **Mass:** 70903 **Score:** 169 **Matches:** 9(8) **Sequences:** 9(8)
unnamed protein product [Mus musculus]
gi|74208631 **Mass:** 70827 **Score:** 169 **Matches:** 9(8) **Sequences:** 9(8)
unnamed protein product [Mus musculus]
gi|74214176 **Mass:** 70828 **Score:** 169 **Matches:** 9(8) **Sequences:** 9(8)
unnamed protein product [Mus musculus]
gi|74220416 **Mass:** 70755 **Score:** 169 **Matches:** 9(8) **Sequences:** 9(8)
unnamed protein product [Mus musculus]
gi|74220592 **Mass:** 70648 **Score:** 169 **Matches:** 9(8) **Sequences:** 9(8)
unnamed protein product [Mus musculus]
gi|74225511 **Mass:** 70783 **Score:** 169 **Matches:** 9(8) **Sequences:** 9(8)
unnamed protein product [Mus musculus]

---

4. gi|2498741 **Mass:** 60964 **Score:** 120 **Matches:** 2(2) **Sequences:** 2(2) **emPAI:** 0.11
RecName: Full=Prolyl 4-hydroxylase subunit alpha-2; Short=4-PH alpha-2; AltName: Full=Procollagen-proline,2-oxoglutarate-4-dioxygenase subunit alpha-2; Flags: Precursor

Query	Observed	Mr(expt)	Mr(calc)	Delta	Miss	Score	Expect	Rank	Unique	Peptide
11452	534.0728	1066.1309	1065.5819	0.5491	0	74	6.3e-005	1	U	K.TGVLTVASYR.V
16319	809.4653	1616.9160	1616.7318	0.1842	0	91	1.9e-006	1	U	K.SSWLEEDDDPVVAR.V

Proteins matching the same set of peptides:
gi|74216495 **Mass:** 21055 **Score:** 120 **Matches:** 2(2) **Sequences:** 2(2)
unnamed protein product [Mus musculus]
gi|148701597 **Mass:** 57417 **Score:** 120 **Matches:** 2(2) **Sequences:** 2(2)
procollagen-proline, 2-oxoglutarate 4-dioxygenase (proline 4-hydroxylase), alpha II polypeptide, isoform CRA_b [Mus musculus]
gi|148701598 **Mass:** 21273 **Score:** 120 **Matches:** 2(2) **Sequences:** 2(2)
procollagen-proline, 2-oxoglutarate 4-dioxygenase (proline 4-hydroxylase), alpha II polypeptide, isoform CRA_c [Mus musculus]
gi|148701599 **Mass:** 61292 **Score:** 120 **Matches:** 2(2) **Sequences:** 2(2)
procollagen-proline, 2-oxoglutarate 4-dioxygenase (proline 4-hydroxylase), alpha II polypeptide, isoform CRA_d [Mus musculus]
gi|148701600 **Mass:** 66870 **Score:** 120 **Matches:** 2(2) **Sequences:** 2(2)
procollagen-proline, 2-oxoglutarate 4-dioxygenase (proline 4-hydroxylase), alpha II polypeptide, isoform CRA_e [Mus musculus]
gi|209862961 **Mass:** 60774 **Score:** 120 **Matches:** 2(2) **Sequences:** 2(2)
prolyl 4-hydroxylase subunit alpha-2 isoform 1 precursor [Mus musculus]
gi|226874876 **Mass:** 60977 **Score:** 120 **Matches:** 2(2) **Sequences:** 2(2)
prolyl 4-hydroxylase subunit alpha-2 isoform 2 precursor [Mus musculus]

---

5. gi|387397 **Mass:** 57807 **Score:** 100 **Matches:** 2(2) **Sequences:** 2(2) **emPAI:** 0.12
epidermal keratin subunit I, partial [Mus musculus]

Query	Observed	Mr(expt)	Mr(calc)	Delta	Miss	Score	Expect	Rank	Unique	Peptide
14668	691.2913	1380.5680	1380.6408	-0.0729	0	68	0.00035	1	U	R.ALEESNYELEGK.I
14765	696.1694	1390.3243	1389.6736	0.6508	0	71	0.00013	1	U	K.QSLEASLAETEGR.Y

Proteins matching the same set of peptides:
gi|12852157 **Mass:** 58587 **Score:** 100 **Matches:** 2(2) **Sequences:** 2(2)
unnamed protein product [Mus musculus]
gi|26349141 **Mass:** 52653 **Score:** 100 **Matches:** 2(2) **Sequences:** 2(2)
unnamed protein product [Mus musculus]
gi|26349459 **Mass:** 49471 **Score:** 100 **Matches:** 2(2) **Sequences:** 2(2)
unnamed protein product [Mus musculus]
gi|112983636 **Mass:** 57007 **Score:** 100 **Matches:** 2(2) **Sequences:** 2(2)
keratin, type I cytoskeletal 10 [Mus musculus]
gi|116242600 **Mass:** 57735 **Score:** 100 **Matches:** 2(2) **Sequences:** 2(2)
RecName: Full=Keratin, type I cytoskeletal 10; AltName: Full=56 kDa cytokeratin; AltName: Full=Cytokeratin-10; Short=CK-10; AltName: Full=Keratin, type I cytoskeletal 59 kDa; AltName: Full=Keratin-1(

---

6. gi|46485130 **Mass:** 85186 **Score:** 79 **Matches:** 2(2) **Sequences:** 2(2) **emPAI:** 0.08
TPA_exp: keratin Kb40 [Mus musculus]

Query	Observed	Mr(expt)	Mr(calc)	Delta	Miss	Score	Expect	Rank	Unique	Peptide
14685	**692.1215**	**1382.2284**	**1382.6830**	**-0.4546**	**0**	**57**	**0.0039**	**1**	**U**	**R.SLNNQFASFIDK.V**
15600	**739.0794**	**1476.1442**	**1475.7984**	**0.3459**	**1**	**60**	**0.0008**	**1**		**R.FLEQQNKVLETK.W**

Proteins matching the same set of peptides:
gi|111185567 **Mass:** 54730 **Score:** 79 **Matches:** 2(2) **Sequences:** 2(2)
Krt78 protein [Mus musculus]
gi|111185722 **Mass:** 56744 **Score:** 79 **Matches:** 2(2) **Sequences:** 2(2)
Krt78 protein [Mus musculus]
gi|119850791 **Mass:** 54739 **Score:** 79 **Matches:** 2(2) **Sequences:** 2(2)
Krt78 protein [Mus musculus]
gi|145580629 **Mass:** 112194 **Score:** 79 **Matches:** 2(2) **Sequences:** 2(2)
keratin Kb40 [Mus musculus]

---

7. gi|85701680 **Mass:** 62806 **Score:** 72 **Matches:** 2(2) **Sequences:** 2(2) **emPAI:** 0.11
keratin, type II cytoskeletal 2 oral [Mus musculus]

Query	Observed	Mr(expt)	Mr(calc)	Delta	Miss	Score	Expect	Rank	Unique	Peptide
11433	**1065.4515**	**1064.4442**	**1064.5138**	**-0.0696**	**0**	**54**	**0.0051**	**1**	**U**	**K.AQYEDIAQK.S**
15600	739.0794	1476.1442	1475.7984	0.3459	1	60	0.0008	1		R.FLEQQNKVLETK.W

Proteins matching the same set of peptides:
gi|148672072 **Mass:** 63519 **Score:** 72 **Matches:** 2(2) **Sequences:** 2(2)
mCG144546 [Mus musculus]

---

8. gi|387399 **Mass:** 10655 **Score:** 64 **Matches:** 1(1) **Sequences:** 1(1) **emPAI:** 0.32
epidermal keratin type I, partial [Mus musculus]

Query	Observed	Mr(expt)	Mr(calc)	Delta	Miss	Score	Expect	Rank	Unique	Peptide
16047	**770.4561**	**2308.3463**	**2308.0567**	**0.2896**	**0**	**64**	**0.00083**	**1**	**U**	**R.LLEGEDAHLSSSQFSSGSQSSR.D**

---

9. gi|16303309 **Mass:** 61743 **Score:** 62 **Matches:** 1(1) **Sequences:** 1(1) **emPAI:** 0.05
type II keratin 5 [Mus musculus]

Query	Observed	Mr(expt)	Mr(calc)	Delta	Miss	Score	Expect	Rank	Unique	Peptide
15266	**721.0629**	**1440.1112**	**1440.6629**	**-0.5517**	**0**	**62**	**0.0013**	**1**	**U**	**R.VDALMDEINFMK.M**

Proteins matching the same set of peptides:
gi|20911031 **Mass:** 61729 **Score:** 62 **Matches:** 1(1) **Sequences:** 1(1)
keratin, type II cytoskeletal 5 [Mus musculus]

---

10. gi|26349293 **Mass:** 76506 **Score:** 61 **Matches:** 1(1) **Sequences:** 1(1) **emPAI:** 0.04
unnamed protein product [Mus musculus]

Query	Observed	Mr(expt)	Mr(calc)	Delta	Miss	Score	Expect	Rank	Unique	Peptide
13424	**624.1697**	**1246.3248**	**1245.6717**	**0.6531**	**0**	**61**	**0.0014**	**1**	**U**	**R.AFINLEAAGVGGK.E**

Proteins matching the same set of peptides:
gi|37360558 **Mass:** 99638 **Score:** 61 **Matches:** 1(1) **Sequences:** 1(1)
mKIAA1815 protein [Mus musculus]
gi|74209380 **Mass:** 100058 **Score:** 61 **Matches:** 1(1) **Sequences:** 1(1)
unnamed protein product [Mus musculus]
gi|124487057 **Mass:** 100084 **Score:** 61 **Matches:** 1(1) **Sequences:** 1(1)
endoplasmic reticulum metallopeptidase 1 [Mus musculus]
gi|148709741 **Mass:** 94565 **Score:** 61 **Matches:** 1(1) **Sequences:** 1(1)
mCG124990, isoform CRA_a [Mus musculus]
gi|148709742 **Mass:** 102045 **Score:** 61 **Matches:** 1(1) **Sequences:** 1(1)
mCG124990, isoform CRA_b [Mus musculus]

---

11. gi|6753620 **Mass:** 73056 **Score:** 61 **Matches:** 1(1) **Sequences:** 1(1) **emPAI:** 0.04
ATP-dependent RNA helicase DDX3X [Mus musculus]

Query	Observed	Mr(expt)	Mr(calc)	Delta	Miss	Score	Expect	Rank	Unique	Peptide
14436	**679.2409**	**1356.4673**	**1356.7765**	**-0.3093**	**0**	**61**	**0.0017**	**1**	**U**	**K.QYPISLVLAPTR.E**

Proteins matching the same set of peptides:
gi|14861844 **Mass:** 73095 **Score:** 61 **Matches:** 1(1) **Sequences:** 1(1)
putative ATP-dependent RNA helicase Pl10 [Mus musculus]
gi|25141235 **Mass:** 73382 **Score:** 61 **Matches:** 1(1) **Sequences:** 1(1)
ATP-dependent RNA helicase DDX3Y [Mus musculus]
gi|74181660 **Mass:** 73339 **Score:** 61 **Matches:** 1(1) **Sequences:** 1(1)
unnamed protein product [Mus musculus]
gi|148706200 **Mass:** 71999 **Score:** 61 **Matches:** 1(1) **Sequences:** 1(1)
DEAD (Asp-Glu-Ala-Asp) box polypeptide 3, Y-linked, isoform CRA_a [Mus musculus]
gi|219521150 **Mass:** 73027 **Score:** 61 **Matches:** 1(1) **Sequences:** 1(1)
Ddx3x protein [Mus musculus]
gi|223462261 **Mass:** 72969 **Score:** 61 **Matches:** 1(1) **Sequences:** 1(1)
Ddx3x protein [Mus musculus]

---

12. gi|47523977 **Mass:** 56715 **Score:** 60 **Matches:** 1(1) **Sequences:** 1(1) **emPAI:** 0.06
keratin, type II cytoskeletal 72 [Mus musculus]

Query	Observed	Mr(expt)	Mr(calc)	Delta	Miss	Score	Expect	Rank	Unique	Peptide
15600	739.0794	1476.1442	1475.7620	0.3822	0	60	0.00096	2	U	R.FLEQQNQVLETK.W

---

13. gi|16716569 **Mass:** 26118 **Score:** 59 **Matches:** 3(3) **Sequences:** 1(1) **emPAI:** 0.13
protease, serine, 1 precursor [Mus musculus]

Query	Observed	Mr(expt)	Mr(calc)	Delta	Miss	Score	Expect	Rank	Unique	Peptide
15580	**738.0673**	**2211.1800**	**2210.0967**	**1.0832**	**0**	**52**	**0.014**	**1**	**U**	**R.LGEHNINVLEGNEQFIDAAK.I** 15575 15577

---

14. gi|1389682 **Mass:** 68068 **Score:** 59 **Matches:** 1(1) **Sequences:** 1(1) **emPAI:** 0.05
plakoglobin, partial [Mus musculus]

Query	Observed	Mr(expt)	Mr(calc)	Delta	Miss	Score	Expect	Rank	Unique	Peptide
15662	**742.6564**	**1483.2982**	**1482.7467**	**0.5515**	**0**	**59**	**0.0027**	**1**	**U**	**R.NEGTATYAAAVLFR.I**

Proteins matching the same set of peptides:
gi|28395018 **Mass:** 81749 **Score:** 59 **Matches:** 1(1) **Sequences:** 1(1)
junction plakoglobin [Mus musculus]

---

15. gi|19354359 **Mass:** 54698 **Score:** 59 **Matches:** 1(1) **Sequences:** 1(1) **emPAI:** 0.06
Ranbp10 protein [Mus musculus]

Query	Observed	Mr(expt)	Mr(calc)	Delta	Miss	Score	Expect	Rank	Unique	Peptide
11862	**552.3564**	**1102.6982**	**1102.5618**	**0.1363**	**0**	**59**	**0.0018**	**1**	**U**	**R.VGEAIETTQR.F**

Proteins matching the same set of peptides:
gi|50510939 **Mass:** 66515 **Score:** 59 **Matches:** 1(1) **Sequences:** 1(1)

mKIAA1464 protein [Mus musculus]
gi|353526275 **Mass:** 67146 **Score:** 59 **Matches:** 1(1) **Sequences:** 1(1)
RecName: Full=Ran-binding protein 10; Short=RanBP10
gi|40804757 **Mass:** 70044 **Score:** 59 **Matches:** 1(1) **Sequences:** 1(1)
ran-binding protein 10 [Mus musculus]

---

16. gi|52789 **Mass:** 54415 **Score:** 57 **Matches:** 1(1) **Sequences:** 1(1) **emPAI:** 0.06
unnamed protein product [Mus musculus]

Query	Observed	Mr(expt)	Mr(calc)	Delta	Miss	Score	Expect	Rank	Unique	Peptide
14685	692.1215	1382.2284	1382.7194	-0.4910	1	57	0.0039	1	U	K.SLNNKFASFIDK.V

Proteins matching the same set of peptides:
gi|309215 **Mass:** 53210 **Score:** 57 **Matches:** 1(1) **Sequences:** 1(1)
EndoA' cytokeratin (5' end put.); putative [Mus musculus]
gi|511654 **Mass:** 54220 **Score:** 57 **Matches:** 1(1) **Sequences:** 1(1)
keratin type II [Mus musculus]
gi|74177777 **Mass:** 54546 **Score:** 57 **Matches:** 1(1) **Sequences:** 1(1)
unnamed protein product [Mus musculus]
gi|74219975 **Mass:** 54459 **Score:** 57 **Matches:** 1(1) **Sequences:** 1(1)
unnamed protein product [Mus musculus]
gi|76779293 **Mass:** 54514 **Score:** 57 **Matches:** 1(1) **Sequences:** 1(1)
Keratin 8 [Mus musculus]
gi|114145561 **Mass:** 54531 **Score:** 57 **Matches:** 1(1) **Sequences:** 1(1)
keratin, type II cytoskeletal 8 [Mus musculus]

---

17. gi|54777 **Mass:** 57108 **Score:** 56 **Matches:** 1(1) **Sequences:** 1(1) **emPAI:** 0.06
unnamed protein product [Mus musculus]

Query	Observed	Mr(expt)	Mr(calc)	Delta	Miss	Score	Expect	Rank	Unique	Peptide
13198	**611.9623**	**1221.9101**	**1221.6162**	**0.2939**	**0**	**56**	**0.0059**	**1**	**U**	**R.LITLEEEMTK.Y**

Proteins matching the same set of peptides:
gi|42415475 **Mass:** 57023 **Score:** 56 **Matches:** 1(1) **Sequences:** 1(1)
protein disulfide-isomerase precursor [Mus musculus]
gi|74141920 **Mass:** 57053 **Score:** 56 **Matches:** 1(1) **Sequences:** 1(1)
unnamed protein product [Mus musculus]
gi|74149659 **Mass:** 43005 **Score:** 56 **Matches:** 1(1) **Sequences:** 1(1)
unnamed protein product [Mus musculus]
gi|74178069 **Mass:** 57099 **Score:** 56 **Matches:** 1(1) **Sequences:** 1(1)
unnamed protein product [Mus musculus]
gi|74190076 **Mass:** 56963 **Score:** 56 **Matches:** 1(1) **Sequences:** 1(1)
unnamed protein product [Mus musculus]
gi|74198312 **Mass:** 57051 **Score:** 56 **Matches:** 1(1) **Sequences:** 1(1)
unnamed protein product [Mus musculus]
gi|74198706 **Mass:** 57000 **Score:** 56 **Matches:** 1(1) **Sequences:** 1(1)
unnamed protein product [Mus musculus]
gi|74203945 **Mass:** 56566 **Score:** 56 **Matches:** 1(1) **Sequences:** 1(1)
unnamed protein product [Mus musculus]
gi|74212231 **Mass:** 57053 **Score:** 56 **Matches:** 1(1) **Sequences:** 1(1)
unnamed protein product [Mus musculus]
gi|74219772 **Mass:** 57008 **Score:** 56 **Matches:** 1(1) **Sequences:** 1(1)
unnamed protein product [Mus musculus]
gi|74220649 **Mass:** 57067 **Score:** 56 **Matches:** 1(1) **Sequences:** 1(1)
unnamed protein product [Mus musculus]
gi|148702818 **Mass:** 58984 **Score:** 56 **Matches:** 1(1) **Sequences:** 1(1)
prolyl 4-hydroxylase, beta polypeptide, isoform CRA_a [Mus musculus]
gi|148702819 **Mass:** 61492 **Score:** 56 **Matches:** 1(1) **Sequences:** 1(1)
prolyl 4-hydroxylase, beta polypeptide, isoform CRA_b [Mus musculus]

---

18. gi|20070408 **Mass:** 113195 **Score:** 55 **Matches:** 1(1) **Sequences:** 1(1) **emPAI:** 0.03

glycine dehydrogenase [decarboxylating], mitochondrial precursor [Mus musculus]

Query	Observed	Mr(expt)	Mr(calc)	Delta	Miss	Score	Expect	Rank	Unique	Peptide
11734	546.4593	1090.9039	1091.4228	-0.5189	0	55	0.0027	1	U	R.DSSGGGGGGGGGDR.G

Proteins matching the same set of peptides:

gi\|148709754 **Mass:** 30341 **Score:** 55 **Matches:** 1(1) **Sequences:** 1(1)
glycine decarboxylase, isoform CRA_c [Mus musculus]

gi\|148709755 **Mass:** 114180 **Score:** 55 **Matches:** 1(1) **Sequences:** 1(1)
glycine decarboxylase, isoform CRA_d [Mus musculus]

gi\|148709756 **Mass:** 98319 **Score:** 55 **Matches:** 1(1) **Sequences:** 1(1)
glycine decarboxylase, isoform CRA_e [Mus musculus]

---

19. gi\|3642689 **Mass:** 9181 **Score:** 54 **Matches:** 1(1) **Sequences:** 1(1) **emPAI:** 0.38
immunoglobulin heavy chain-binding protein, partial [Mus musculus]

Query	Observed	Mr(expt)	Mr(calc)	Delta	Miss	Score	Expect	Rank	Unique	Peptide
15298	722.9624	1443.9102	1443.6994	0.2108	0	54	0.0046	1	U	-.TWNDPTVQQDIK.F

---

20. gi\|26344548 **Mass:** 58526 **Score:** 54 **Matches:** 1(1) **Sequences:** 1(1) **emPAI:** 0.06
unnamed protein product [Mus musculus]

Query	Observed	Mr(expt)	Mr(calc)	Delta	Miss	Score	Expect	Rank	Unique	Peptide
11185	521.0774	1040.1402	1039.6390	0.5013	0	54	0.003	1	U	K.VLEALQVLR.C

Proteins matching the same set of peptides:

gi\|35193112 **Mass:** 55286 **Score:** 54 **Matches:** 1(1) **Sequences:** 1(1)
Wdr26 protein [Mus musculus]

gi\|148681181 **Mass:** 58625 **Score:** 54 **Matches:** 1(1) **Sequences:** 1(1)
WD repeat domain 26 [Mus musculus]

gi\|264681550 **Mass:** 70499 **Score:** 54 **Matches:** 1(1) **Sequences:** 1(1)
WD repeat-containing protein 26 [Mus musculus]

---

21. gi\|836898 **Mass:** 59960 **Score:** 54 **Matches:** 2(1) **Sequences:** 2(1) **emPAI:** 0.11
prolyl 4-hydroxylase alpha(I)-subunit, partial [Mus musculus]

Query	Observed	Mr(expt)	Mr(calc)	Delta	Miss	Score	Expect	Rank	Unique	Peptide
12213	567.0950	1132.1754	1131.5924	0.5830	0	49	0.011	1	U	K.GIAVDYLPER.Q
13453	625.1786	1248.3426	1248.6462	-0.3036	0	43	0.091	1	U	K.LLELDPEHQR.A

Proteins matching the same set of peptides:

gi\|26336999 **Mass:** 60848 **Score:** 54 **Matches:** 2(1) **Sequences:** 2(1)
unnamed protein product [Mus musculus]

gi\|33859596 **Mass:** 60872 **Score:** 54 **Matches:** 2(1) **Sequences:** 2(1)
prolyl 4-hydroxylase subunit alpha-1 precursor [Mus musculus]

gi\|74224984 **Mass:** 60886 **Score:** 54 **Matches:** 2(1) **Sequences:** 2(1)
unnamed protein product [Mus musculus]

gi\|74225936 **Mass:** 63769 **Score:** 54 **Matches:** 2(1) **Sequences:** 2(1)
unnamed protein product [Mus musculus]

---

22. gi\|4159806 **Mass:** 65183 **Score:** 52 **Matches:** 1(1) **Sequences:** 1(1) **emPAI:** 0.05
type II keratin subunit protein [Mus musculus]

Query	Observed	Mr(expt)	Mr(calc)	Delta	Miss	Score	Expect	Rank	Unique	Peptide
13588	633.1061	1264.1977	1264.6299	-0.4322	0	52	0.01	1	U	R.TNAENEFVTIK.K

Proteins matching the same set of peptides:

gi\|12859782 **Mass:** 65586 **Score:** 52 **Matches:** 1(1) **Sequences:** 1(1)
unnamed protein product [Mus musculus]

gi\|126116585 **Mass:** 65565 **Score:** 52 **Matches:** 1(1) **Sequences:** 1(1)
keratin, type II cytoskeletal 1 [Mus musculus]

---

23. gi|37935731 **Mass:** 192078 **Score:** 52 **Matches:** 1(1) **Sequences:** 1(1) **emPAI:** 0.02
ATP-binding cassette transporter sub-family A member 16 [Mus musculus]

Query	Observed	Mr(expt)	Mr(calc)	Delta	Miss	Score	Expect	Rank	Unique	Peptide
13521	629.4885	1256.9625	1256.4835	0.4790	0	52	0.012	1	U	R.MEECEALCTR.L

Proteins matching the same set of peptides:
gi|148685261 **Mass:** 167887 **Score:** 52 **Matches:** 1(1) **Sequences:** 1(1)
ATP-binding cassette, sub-family A (ABC1), member 16 [Mus musculus]

24. gi|21595163 **Mass:** 71011 **Score:** 48 **Matches:** 1(1) **Sequences:** 1(1) **emPAI:** 0.05
Glycosyltransferase 25 domain containing 1 [Mus musculus]

Query	Observed	Mr(expt)	Mr(calc)	Delta	Miss	Score	Expect	Rank	Unique	Peptide
9480	435.0096	868.0046	867.5905	0.4140	0	48	0.0098	1	U	R.VLIALLAR.N

Proteins matching the same set of peptides:
gi|148697003 **Mass:** 54889 **Score:** 48 **Matches:** 1(1) **Sequences:** 1(1)
glycosyltransferase 25 domain containing 1, isoform CRA_b [Mus musculus]
gi|170784829 **Mass:** 71015 **Score:** 48 **Matches:** 1(1) **Sequences:** 1(1)
procollagen galactosyltransferase 1 precursor [Mus musculus]
gi|74217150 **Mass:** 70997 **Score:** 48 **Matches:** 1(1) **Sequences:** 1(1)
unnamed protein product [Mus musculus]

25. gi|12847276 **Score:** 48 **Matches:** 1(1) **Sequences:** 1(1) **emPAI:** 0.07
unnamed protein product [Mus musculus]

Query	Observed	Mr(expt)	Mr(calc)	Delta	Miss	Score	Expect	Rank	Unique	Peptide
15600	739.0794	1476.1442	1475.7442	0.4000	1	48	0.013	3	U	R.YEPKCPLGVDISR.E

Proteins matching the same set of peptides:
gi|31560081 **Score:** 48 **Matches:** 1(1) **Sequences:** 1(1)
gi|148667576 **Score:** 48 **Matches:** 1(1) **Sequences:** 1(1)

26. gi|74191500 **Mass:** 57022 **Score:** 46 **Matches:** 1(1) **Sequences:** 1(1) **emPAI:** 0.06
unnamed protein product [Mus musculus]

Query	Observed	Mr(expt)	Mr(calc)	Delta	Miss	Score	Expect	Rank	Unique	Peptide
13198	611.9623	1221.9101	1220.6686	1.2415	1	46	0.05	2	U	R.LITLEEKMTK.Y

27. gi|81875970 **Mass:** 71638 **Score:** 45 **Matches:** 1(0) **Sequences:** 1(0) **emPAI:** 0.05
RecName: Full=Alkyldihydroxyacetonephosphate synthase, peroxisomal; Short=Alkyl-DHAP synthase; AltName: Full=Alkylglycerone-phosphate synthase; Flags: Precursor

Query	Observed	Mr(expt)	Mr(calc)	Delta	Miss	Score	Expect	Rank	Unique	Peptide
15186	717.2085	2148.6037	2149.1127	-0.5090	1	45	0.06	1	U	R.RAASAAGASPAATPAAPESGTIPK.K

Proteins matching the same set of peptides:
gi|148695250 **Mass:** 53219 **Score:** 45 **Matches:** 1(0) **Sequences:** 1(0)
alkylglycerone phosphate synthase, isoform CRA_a [Mus musculus]
gi|295444834 **Mass:** 74296 **Score:** 45 **Matches:** 1(0) **Sequences:** 1(0)
alkyldihydroxyacetonephosphate synthase, peroxisomal [Mus musculus]

28. gi|7513694 **Mass:** 109137 **Score:** 44 **Matches:** 1(0) **Sequences:** 1(0) **emPAI:** 0.03
IgG Fc binding protein - mouse (fragment)

Query	Observed	Mr(expt)	Mr(calc)	Delta	Miss	Score	Expect	Rank	Unique	Peptide
9539	874.3927	873.3854	873.4920	-0.1066	0	44	0.06	1	U	R.ISVINGGSK.A

Proteins matching the same set of peptides:
gi|148692210 **Mass:** 147962 **Score:** 44 **Matches:** 1(0) **Sequences:** 1(0)
mCG145390, isoform CRA_a [Mus musculus]
gi|148692211 **Mass:** 146729 **Score:** 44 **Matches:** 1(0) **Sequences:** 1(0)

mCG145390, isoform CRA_b [Mus musculus]
gi|169790797 **Mass:** 275058 **Score:** 44 **Matches:** 1(0) **Sequences:** 1(0)
Fc fragment of IgG binding protein precursor [Mus musculus]
gi|257467625 **Mass:** 280044 **Score:** 44 **Matches:** 1(0) **Sequences:** 1(0)
Fc fragment of IgG binding protein-like precursor [Mus musculus]

---

29. gi|148664969 **Mass:** 26715 **Score:** 44 **Matches:** 1(1) **Sequences:** 1(1) **emPAI:** 0.12
RIKEN cDNA 2900011O08, isoform CRA_a [Mus musculus]

Query	Observed	Mr(expt)	Mr(calc)	Delta	Miss	Score	Expect	Rank	Unique	Peptide
12819	593.2423	1184.4701	1185.5812	-1.1111	0	44	0.053	1	U	-.NTADLSCLPPR.S

---

30. gi|12855410 **Mass:** 22073 **Score:** 43 **Matches:** 1(1) **Sequences:** 1(1) **emPAI:** 0.15
unnamed protein product [Mus musculus]

Query	Observed	Mr(expt)	Mr(calc)	Delta	Miss	Score	Expect	Rank	Unique	Peptide
13489	627.3521	1252.6896	1252.6526	0.0370	1	43	0.056	1	U	R.EKMVVFFPPK.E

Mascot: http://www.matrixscience.com/

PFHR9

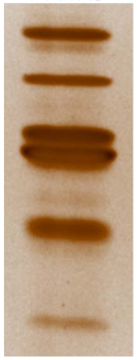

3

{MATRIX SCIENCE} Mascot Search Results

User	:
Email	:
Search title	:
MS data file	: ZIEN-29Nov2017_3m.mgf
Database	: NCBInr 20120419 (17893860 sequences; 6141683785 residues)
Taxonomy	: Mus musculus (house mouse) (138831 sequences)
Timestamp	: 30 Nov 2017 at 20:39:15 GMT
Enzyme	: Trypsin
Variable modifications	: Carbamidomethyl (C),Oxidation (M),Oxidation (P)
Mass values	: Monoisotopic
Protein Mass	: Unrestricted
Peptide Mass Tolerance	: ± 1.25 Da
Fragment Mass Tolerance	: ± 1.001 Da
Max Missed Cleavages	: 1
Instrument type	: Default
Number of queries	: 19332
Protein hits	: gi|54777 unnamed protein product [Mus musculus]
	gi|148701597 procollagen-proline, 2-oxoglutarate 4-dioxygenase (proline 4-hydroxylase), alpha II polypeptide, isoform CRA_b [Mus musculus]
	gi|148701600 procollagen-proline, 2-oxoglutarate 4-dioxygenase (proline 4-hydroxylase), alpha II polypeptide, isoform CRA_e [Mus musculus]
	gi|74225936 unnamed protein product [Mus musculus]
	gi|16716569 protease, serine, 1 precursor [Mus musculus]
	gi|347839 matricin [Mus musculus]
	gi|21312570 protein ERGIC-53 precursor [Mus musculus]
	gi|387398 epidermal keratin type I, partial [Mus musculus]
	gi|51452 unnamed protein product [Mus musculus]
	gi|74213832 unnamed protein product [Mus musculus]
	gi|22164776 keratin, type II cytoskeletal 79 [Mus musculus]
	gi|12843914 unnamed protein product [Mus musculus]
	gi|5295992 chaperonin containing TCP-1 theta subunit [Mus musculus]
	gi|1419012 CCT zeta2, zeta2 subunit of the chaperonin containing TCP-1 (CCT) [Mus musculus]
	gi|7710044 kelch-like ECH-associated protein 1 [Mus musculus]
	gi|2623222 ATP synthase beta-subunit [Mus musculus]
	gi|192262 pro-alpha-1 type I collagen, partial [Mus musculus]
	gi|551295 pyruvate kinase M [Mus musculus]
	gi|52789 unnamed protein product [Mus musculus]
	gi|46485130 TPA_exp: keratin Kb40 [Mus musculus]
	gi|1304157 78 kDa glucose-regulated protein [Mus musculus]
	gi|4159806 type II keratin subunit protein [Mus musculus]
	gi|293686 epidermal keratin subunit II [Mus musculus]
	gi|3687320 cartilage-associated protein (CASP) [Mus musculus]
	gi|74203983 unnamed protein product [Mus musculus]
	gi|468546 CCT (chaperonin containing TCP-1) beta subunit [Mus musculus]
	gi|22297268 AID, partial [Homo sapiens]
	gi|51450 heat shock protein [Mus musculus]
	gi|6680748 ATP synthase subunit alpha, mitochondrial precursor [Mus musculus]
	gi|148688671 mCG16097 [Mus musculus]
	gi|6755893 trypsin 4 precursor [Mus musculus]
	gi|148707748 nuclear casein kinase and cyclin-dependent kinase substrate 1, isoform CRA_b [Mus musculus]
	gi|53830774 lactase [Mus musculus]
	gi|26324736 unnamed protein product [Mus musculus]
	gi|7513694 IgG Fc binding protein - mouse (fragment)
	gi|111599858 Dehydrogenase E1 and transketolase domain containing 1 [Mus musculus]
	gi|74224513 unnamed protein product [Mus musculus]
	gi|21410133 Kidins220 protein [Mus musculus]
	gi|26333649 unnamed protein product [Mus musculus]
	gi|986918 A10 [Mus musculus]
	gi|21313084 chromodomain Y-like protein 2 [Mus musculus]
	gi|124504325 Armcx4 protein [Mus musculus]
	gi|12843046 unnamed protein product [Mus musculus]
	gi|12836661 unnamed protein product [Mus musculus]

gi|148681245 mCG145199 [Mus musculus]
gi|148667455 TEA domain family member 4, isoform CRA_b [Mus musculus]
gi|642072 fibrillin-1, partial [Mus musculus]
gi|12836791 unnamed protein product [Mus musculus]
gi|14250408 Aspartyl-tRNA synthetase [Mus musculus]
gi|17512475 Ankyrin repeat and SOCS box-containing 6 [Mus musculus]
gi|309271872 PREDICTED: zinc finger protein 469 [Mus musculus]
gi|26380166 unnamed protein product [Mus musculus]

Select Summary Report

Format As | Select Summary (protein hits) ▼ | Help

Significance threshold p< 0.05 Max. number of hits AUTO

Standard scoring ○ MudPIT scoring ◉ Ions score or expect cut-off 42 Show sub-sets 0

Show pop-ups ◉ Suppress pop-ups ○ Require bold red ☐

Re-Search ◉ All queries ○ Unassigned ○ Below homology threshold ○ Below identity threshold

1. gi|54777 **Mass:** 57108 **Score:** 426 **Matches:** 28(25) **Sequences:** 16(15) **emPAI:** 1.90
unnamed protein product [Mus musculus]

Query	Observed	Mr(expt)	Mr(calc)	Delta	Miss	Score	Expect	Rank	Unique	Peptide
10277	381.1882	760.3618	760.3715	-0.0097	0	50	0.015	1	U	K.AEGSEIR.L
10911	395.3300	788.6454	788.3664	0.2790	0	59	0.0018	1	U	K.NGDTASPK.E
13577	483.6877	965.3609	965.5586	-0.1977	0	55	0.0037	1	U	R.ILEFFGLK.K
14327	1051.4263	1050.4190	1050.4982	-0.0791	0	51	0.0088	1	U	R.NNFEGEITK.E
14499	1067.7178	1066.7105	1065.5091	1.2014	0	45	0.033	1	U	R.TVIDYNGER.T
14641	1081.6606	1080.6533	1080.6695	-0.0162	0	52	0.007	1	U	K.THILLFLPK.S 14649
14648	541.6124	1081.2102	1080.6695	0.5406	0	(48)	0.012	1	U	K.THILLFLPK.S 14646
16005	1203.5746	1202.5673	1201.5979	0.9694	0	49	0.017	1	U	R.EADDIVNWLK.K
16043	604.0506	1206.0866	1205.6213	0.4653	0	51	0.0064	1	U	R.LITLEEEMTK.Y 16046
16166	608.9402	1215.8658	1215.5884	0.2775	0	47	0.025	1	U	K.SNFEEALAAHK.Y
16232	611.6342	1221.2538	1221.6162	-0.3625	0	(44)	0.088	1	U	R.LITLEEEMTK.Y
16991	647.9220	1293.8294	1292.5918	1.2376	0	84	4.2e-006	1	U	K.MDSTANEVEAVK.V 16980 16985 16986 16990
17115	654.5397	1307.0648	1307.6357	-0.5709	1	46	0.043	1	U	R.NNFEGEITKEK.L
18095	475.6379	1423.8917	1423.7711	0.1206	1	(46)	0.031	1	U	K.YQLDKDGVVLFK.K
18099	713.1129	1424.2113	1423.7711	0.4402	1	55	0.0069	1	U	K.YQLDKDGVVLFK.K 18100
18159	717.4308	1432.8470	1431.6882	1.1589	1	63	0.0011	1	U	K.SVSDYDGKLSSFK.R
15815	594.4030	1780.1872	1779.8275	0.3597	0	(61)	0.0019	1	U	K.VDATEESDLAQQYGVR.G
19294	891.2250	1780.4354	1779.8275	0.6079	0	78	2.7e-005	1	U	K.VDATEESDLAQQYGVR.G
16231	611.6050	1831.7933	1832.9057	-1.1124	0	43	0.1	1	U	K.ILFIFIDSDHTDNQR.I
19326	1493.5814	2985.1482	2984.4866	0.6616	0	98	2.4e-007	1	U	R.TGPAATTLSDTAAAESLVDSSEVTVIGFFK.D

Proteins matching the same set of peptides:

gi|74212231 **Mass:** 57053 **Score:** 426 **Matches:** 28(25) **Sequences:** 16(15)
unnamed protein product [Mus musculus]

gi|74190076 **Mass:** 56963 **Score:** 426 **Matches:** 28(25) **Sequences:** 16(15)
unnamed protein product [Mus musculus]

gi|42415475 **Mass:** 57023 **Score:** 426 **Matches:** 28(25) **Sequences:** 16(15)
protein disulfide-isomerase precursor [Mus musculus]

gi|74141920 **Mass:** 57053 **Score:** 426 **Matches:** 28(25) **Sequences:** 16(15)
unnamed protein product [Mus musculus]

gi|74178069 **Mass:** 57099 **Score:** 426 **Matches:** 28(25) **Sequences:** 16(15)
unnamed protein product [Mus musculus]
gi|74198312 **Mass:** 57051 **Score:** 426 **Matches:** 28(25) **Sequences:** 16(15)
unnamed protein product [Mus musculus]
gi|74198706 **Mass:** 57000 **Score:** 426 **Matches:** 28(25) **Sequences:** 16(15)
unnamed protein product [Mus musculus]
gi|74219772 **Mass:** 57008 **Score:** 426 **Matches:** 28(25) **Sequences:** 16(15)
unnamed protein product [Mus musculus]
gi|148702818 **Mass:** 58984 **Score:** 426 **Matches:** 28(25) **Sequences:** 16(15)
prolyl 4-hydroxylase, beta polypeptide, isoform CRA_a [Mus musculus]
gi|148702819 **Mass:** 61492 **Score:** 426 **Matches:** 28(25) **Sequences:** 16(15)
prolyl 4-hydroxylase, beta polypeptide, isoform CRA_b [Mus musculus]

---

2. gi|148701597 **Mass:** 57417 **Score:** 223 **Matches:** 17(15) **Sequences:** 10(10) **emPAI:** 0.75
procollagen-proline, 2-oxoglutarate 4-dioxygenase (proline 4-hydroxylase), alpha II polypeptide, isoform CRA_b [Mus musculus]

Query	Observed	Mr(expt)	Mr(calc)	Delta	Miss	Score	Expect	Rank	Unique	Peptide
13332	**470.4389**	**938.8633**	**938.3981**	**0.4651**	**0**	**51**	**0.006**	**1**	**U**	**R.SDEQDAFK.R**
14757	**1093.6360**	**1092.6287**	**1092.5703**	**0.0585**	**0**	**44**	**0.054**	**1**		**K.EYILVEEAK.L** 14753
14783	**548.5247**	**1095.0348**	**1094.4992**	**0.5356**	**1**	**44**	**0.031**	**1**	**U**	**R.SDEQDAFKR.L**
15163	**1125.7443**	**1124.7370**	**1124.6958**	**0.0413**	**0**	**46**	**0.028**	**1**		**R.VPQLLIAPFK.E**
15611	**583.9658**	**1165.9171**	**1165.6091**	**0.3080**	**0**	**48**	**0.013**	**1**		**R.LLSLDPSHER.A**
16687	**631.0879**	**1260.1612**	**1260.6198**	**-0.4585**	**0**	**68**	**0.00026**	**1**		**K.QLDAGEEATVTK.S** 16695 16699
18396	**734.0641**	**1466.1136**	**1466.8034**	**-0.6898**	**1**	**59**	**0.0027**	**1**		**K.KGTAVFWYNLLR.S** 18398
18833	**774.6111**	**1547.2077**	**1547.6450**	**-0.4372**	**0**	**55**	**0.0061**	**1**		**R.YYDVMSDEEIER.I** 18840 18842
19065	**805.0440**	**1608.0735**	**1607.7250**	**0.3485**	**0**	**47**	**0.046**	**1**		**R.GQEFLRPCGTTEVD.-** 19066
19092	**809.5747**	**1617.1349**	**1616.7318**	**0.4030**	**0**	**69**	**0.00021**	**1**		**K.SSWLEEDDDPVVAR.V**

Proteins matching the same set of peptides:
gi|226874876 **Mass:** 60977 **Score:** 223 **Matches:** 17(15) **Sequences:** 10(10)
prolyl 4-hydroxylase subunit alpha-2 isoform 2 precursor [Mus musculus]

---

3. gi|148701600 **Mass:** 66870 **Score:** 216 **Matches:** 16(14) **Sequences:** 9(9) **emPAI:** 0.54
procollagen-proline, 2-oxoglutarate 4-dioxygenase (proline 4-hydroxylase), alpha II polypeptide, isoform CRA_e [Mus musculus]

Query	Observed	Mr(expt)	Mr(calc)	Delta	Miss	Score	Expect	Rank	Unique	Peptide
13143	**460.4962**	**918.9779**	**918.4923**	**0.4856**	**0**	**47**	**0.016**	**1**	**U**	**R.RPFDSGLK.T**
14757	1093.6360	1092.6287	1092.5703	0.0585	0	44	0.054	1		K.EYILVEEAK.L 14753
15163	1125.7443	1124.7370	1124.6958	0.0413	0	46	0.028	1		R.VPQLLIAPFK.E
15611	583.9658	1165.9171	1165.6091	0.3080	0	48	0.013	1		R.LLSLDPSHER.A
16687	631.0879	1260.1612	1260.6198	-0.4585	0	68	0.00026	1		K.QLDAGEEATVTK.S 16695 16699
18396	734.0641	1466.1136	1466.8034	-0.6898	1	59	0.0027	1		K.KGTAVFWYNLLR.S 18398
18833	774.6111	1547.2077	1547.6450	-0.4372	0	55	0.0061	1		R.YYDVMSDEEIER.I 18840 18842
19065	805.0440	1608.0735	1607.7250	0.3485	0	47	0.046	1		R.GQEFLRPCGTTEVD.- 19066
19092	809.5747	1617.1349	1616.7318	0.4030	0	69	0.00021	1		K.SSWLEEDDDPVVAR.V

Proteins matching the same set of peptides:
gi|209862961 **Mass:** 60774 **Score:** 216 **Matches:** 16(14) **Sequences:** 9(9)
prolyl 4-hydroxylase subunit alpha-2 isoform 1 precursor [Mus musculus]

---

4. gi|74225936 **Mass:** 63769 **Score:** 160 **Matches:** 10(8) **Sequences:** 8(6) **emPAI:** 0.42
unnamed protein product [Mus musculus]

Query	Observed	Mr(expt)	Mr(calc)	Delta	Miss	Score	Expect	Rank	Unique	Peptide
13568	483.3983	964.7821	964.5090	0.2731	0	50	0.0085	1	U	K.GNLPGVQHK.S
14305	524.3110	1046.6075	1045.5590	1.0485	0	44	0.11	1	U	R.RPCTLSELK.S
15244	1132.6241	1131.6168	1131.5924	0.0244	0	44	0.05	1	U	K.GIAVDYLPER.Q
15607	583.4688	1164.9231	1164.6074	0.3157	0	68	0.00014	1	U	R.HAACPVLVGNK.W
16994	648.0558	1294.0970	1293.7180	0.3790	1	47	0.017	1	U	K.DLVTSLKDYIK.A
18891	783.3275	1564.6405	1564.7522	-0.1117	0	53	0.013	1	U	K.SAWLSGYEDPVVSR.I
17262	662.6197	1984.8374	1984.0490	0.7884	1	44	0.076	1	U	R.RATISNPVTGALETVHYR.I
18487	739.6022	2215.7847	2215.1274	0.6573	1	58	0.0028	1	U	R.LTSTATKDPEGFVGHPVNAFK.L 18476 18483

---

5. gi|16716569 **Mass:** 26118 **Score:** 136 **Matches:** 5(4) **Sequences:** 1(1) **emPAI:** 0.27
protease, serine, 1 precursor [Mus musculus]

Query	Observed	Mr(expt)	Mr(calc)	Delta	Miss	Score	Expect	Rank	Unique	Peptide
18449	737.5288	2209.5646	2210.0967	-0.5321	0	(67)	0.00036	1	U	R.LGEHNINVLEGNEQFIDAAK.I 18450 18452 18459
19321	1106.1886	2210.3626	2210.0967	0.2659	0	78	2.9e-005	1	U	R.LGEHNINVLEGNEQFIDAAK.I

---

6. gi|347839 **Mass:** 60450 **Score:** 101 **Matches:** 2(2) **Sequences:** 2(2) **emPAI:** 0.11
matricin [Mus musculus]

Query	Observed	Mr(expt)	Mr(calc)	Delta	Miss	Score	Expect	Rank	Unique	Peptide
15211	565.4988	1128.9830	1128.6251	0.3579	1	73	4.7e-005	1	U	R.KVQSGNINAAK.T
16416	619.2510	1236.4874	1236.6094	-0.1220	0	70	0.00023	1	U	R.MALDDMISTLK.K

Proteins matching the same set of peptides:
gi|6753320 **Mass:** 60591 **Score:** 101 **Matches:** 2(2) **Sequences:** 2(2)
T-complex protein 1 subunit gamma [Mus musculus]
gi|74142612 **Mass:** 58241 **Score:** 101 **Matches:** 2(2) **Sequences:** 2(2)
unnamed protein product [Mus musculus]
gi|74226937 **Mass:** 60563 **Score:** 101 **Matches:** 2(2) **Sequences:** 2(2)
unnamed protein product [Mus musculus]

---

7. gi|21312570 **Mass:** 57753 **Score:** 91 **Matches:** 3(3) **Sequences:** 2(2) **emPAI:** 0.12
protein ERGIC-53 precursor [Mus musculus]

Query	Observed	Mr(expt)	Mr(calc)	Delta	Miss	Score	Expect	Rank	Unique	Peptide
16899	642.4886	1282.9626	1282.6405	0.3222	0	66	0.00044	1	U	R.YVSSLTEEISR.R 16889
18234	723.1138	2166.3195	2165.9978	0.3217	0	50	0.019	1	U	K.GHPDLQGQPADDIFESIGDR.E

Proteins matching the same set of peptides:
gi|74152119 **Mass:** 61236 **Score:** 91 **Matches:** 3(3) **Sequences:** 2(2)
unnamed protein product [Mus musculus]

---

8. gi|387398 **Mass:** 41454 **Score:** 83 **Matches:** 2(2) **Sequences:** 1(1) **emPAI:** 0.08
epidermal keratin type I, partial [Mus musculus]

Query	Observed	Mr(expt)	Mr(calc)	Delta	Miss	Score	Expect	Rank	Unique	Peptide
14151	515.5348	1029.0550	1028.5866	0.4684	0	66	0.00022	1	U	R.VLDELTLAR.A 14152

Proteins matching the same set of peptides:
gi|741022 **Mass:** 49086 **Score:** 83 **Matches:** 2(2) **Sequences:** 1(1)
keratin 15
gi|904215 **Mass:** 49129 **Score:** 83 **Matches:** 2(2) **Sequences:** 1(1)

cytokeratin 15 [Mus musculus]
gi|1083398 **Mass:** 7958 **Score:** 83 **Matches:** 2(2) **Sequences:** 1(1)
keratin 15, type I, cytoskeletal - mouse (fragments)
gi|6680606 **Mass:** 44515 **Score:** 83 **Matches:** 2(2) **Sequences:** 1(1)
keratin, type I cytoskeletal 19 [Mus musculus]
gi|7106335 **Mass:** 48132 **Score:** 83 **Matches:** 2(2) **Sequences:** 1(1)
keratin, type I cytoskeletal 17 [Mus musculus]
gi|13097093 **Mass:** 30629 **Score:** 83 **Matches:** 2(2) **Sequences:** 1(1)
Krt14 protein [Mus musculus]
gi|21489935 **Mass:** 52834 **Score:** 83 **Matches:** 2(2) **Sequences:** 1(1)
keratin, type I cytoskeletal 14 [Mus musculus]
gi|38503465 **Mass:** 50146 **Score:** 83 **Matches:** 2(2) **Sequences:** 1(1)
keratin 17n [Mus musculus]
gi|83304265 **Mass:** 49107 **Score:** 83 **Matches:** 2(2) **Sequences:** 1(1)
RecName: Full=Keratin, type I cytoskeletal 15; AltName: Full=Cytokeratin-15; Short=CK-15; AltName: Full=Keratin-15; Short=K15
gi|85726518 **Mass:** 49857 **Score:** 83 **Matches:** 2(2) **Sequences:** 1(1)
Krt42 protein [Mus musculus]
gi|148670626 **Mass:** 54668 **Score:** 83 **Matches:** 2(2) **Sequences:** 1(1)
mCG144006 [Mus musculus]
gi|154090941 **Mass:** 50102 **Score:** 83 **Matches:** 2(2) **Sequences:** 1(1)
keratin, type I cytoskeletal 42 [Mus musculus]
gi|156139032 **Mass:** 50162 **Score:** 83 **Matches:** 2(2) **Sequences:** 1(1)
Keratin 42 [Mus musculus]
gi|226823220 **Mass:** 49463 **Score:** 83 **Matches:** 2(2) **Sequences:** 1(1)
keratin, type I cytoskeletal 15 [Mus musculus]

---

9. gi|51452 **Mass:** 58833 **Score:** 66 **Matches:** 2(2) **Sequences:** 2(2) **emPAI:** 0.11
unnamed protein product [Mus musculus]

Query	Observed	Mr(expt)	Mr(calc)	Delta	Miss	Score	Expect	Rank	Unique	Peptide
17855	695.5581	1389.1017	1388.6976	0.4041	0	52	0.012	1	U	R.GYISPYFINTSK.G
18659	752.6903	1503.3661	1503.7490	-0.3830	0	53	0.011	1	U	K.TLNDELEIIEGMK.F

Proteins matching the same set of peptides:
gi|51455 **Mass:** 60903 **Score:** 66 **Matches:** 2(2) **Sequences:** 2(2)
heat shock protein 65 [Mus musculus]
gi|26353954 **Mass:** 60918 **Score:** 66 **Matches:** 2(2) **Sequences:** 2(2)
unnamed protein product [Mus musculus]
gi|76779273 **Mass:** 59388 **Score:** 66 **Matches:** 2(2) **Sequences:** 2(2)
Hspd1 protein, partial [Mus musculus]
gi|183396771 **Mass:** 60917 **Score:** 66 **Matches:** 2(2) **Sequences:** 2(2)
60 kDa heat shock protein, mitochondrial [Mus musculus]

---

10. gi|74213832 **Mass:** 52871 **Score:** 65 **Matches:** 1(1) **Sequences:** 1(1) **emPAI:** 0.06
unnamed protein product [Mus musculus]

Query	Observed	Mr(expt)	Mr(calc)	Delta	Miss	Score	Expect	Rank	Unique	Peptide
17796	692.4768	1382.9391	1382.5772	0.3618	0	65	0.00055	1	U	R.GGAEQFMEETER.S

Proteins matching the same set of peptides:
gi|148666718 **Mass:** 43932 **Score:** 65 **Matches:** 1(1) **Sequences:** 1(1)
chaperonin subunit 7 (eta), isoform CRA_c [Mus musculus]
gi|26346713 **Mass:** 59598 **Score:** 65 **Matches:** 1(1) **Sequences:** 1(1)

unnamed protein product [Mus musculus]
gi|74147234 **Mass:** 59610 **Score:** 65 **Matches:** 1(1) **Sequences:** 1(1)
unnamed protein product [Mus musculus]
gi|74151643 **Mass:** 59646 **Score:** 65 **Matches:** 1(1) **Sequences:** 1(1)
unnamed protein product [Mus musculus]
gi|74191272 **Mass:** 59600 **Score:** 65 **Matches:** 1(1) **Sequences:** 1(1)
unnamed protein product [Mus musculus]
gi|74212111 **Mass:** 59614 **Score:** 65 **Matches:** 1(1) **Sequences:** 1(1)
unnamed protein product [Mus musculus]
gi|148666717 **Mass:** 61343 **Score:** 65 **Matches:** 1(1) **Sequences:** 1(1)
chaperonin subunit 7 (eta), isoform CRA_b [Mus musculus]
gi|238814391 **Mass:** 59614 **Score:** 65 **Matches:** 1(1) **Sequences:** 1(1)
T-complex protein 1 subunit eta [Mus musculus]

---

11. gi|22164776 **Mass:** 57517 **Score:** 65 **Matches:** 2(1) **Sequences:** 2(1) **emPAI:** 0.06
keratin, type II cytoskeletal 79 [Mus musculus]

Query	Observed	Mr(expt)	Mr(calc)	Delta	Miss	Score	Expect	Rank	Unique	Peptide
16899	642.4886	1282.9626	1283.5491	-0.5864	0	43	0.084	2	U	K.GGFSSNSASGGGGSR.M
17315	**665.6135**	**1329.2125**	**1328.7187**	**0.4938**	**0**	**65**	**0.00064**	**1**	**U**	**R.NLDLDSIIAEVK.A**

Proteins matching the same set of peptides:
gi|148672069 **Mass:** 59231 **Score:** 65 **Matches:** 2(1) **Sequences:** 2(1)
cDNA sequence BC031593 [Mus musculus]

---

12. gi|12843914 **Mass:** 29993 **Score:** 63 **Matches:** 2(2) **Sequences:** 1(1) **emPAI:** 0.11
unnamed protein product [Mus musculus]

Query	Observed	Mr(expt)	Mr(calc)	Delta	Miss	Score	Expect	Rank	Unique	Peptide
17066	**652.3516**	**1302.6886**	**1301.7078**	**0.9807**	**0**	**56**	**0.0062**	**1**	**U**	**R.SLDLDSIIAEVK.A** 17059

Proteins matching the same set of peptides:
gi|16303309 **Mass:** 61743 **Score:** 63 **Matches:** 2(2) **Sequences:** 1(1)
type II keratin 5 [Mus musculus]
gi|20911031 **Mass:** 61729 **Score:** 63 **Matches:** 2(2) **Sequences:** 1(1)
keratin, type II cytoskeletal 5 [Mus musculus]
gi|54607171 **Mass:** 59299 **Score:** 63 **Matches:** 2(2) **Sequences:** 1(1)
keratin, type II cytoskeletal 6A [Mus musculus]
gi|59798479 **Mass:** 60285 **Score:** 63 **Matches:** 2(2) **Sequences:** 1(1)
RecName: Full=Keratin, type II cytoskeletal 6B; AltName: Full=Cytokeratin-6B; Short=CK-6B; AltName: Full=Keratin-6-beta; Short=mK6-beta; AltName: Full=Keratin-6B; Short=K6B
gi|110645788 **Mass:** 60236 **Score:** 63 **Matches:** 2(2) **Sequences:** 1(1)
Krt6b protein [Mus musculus]
gi|113195684 **Mass:** 59490 **Score:** 63 **Matches:** 2(2) **Sequences:** 1(1)
keratin, type II cytoskeletal 6B [Mus musculus]
gi|116063325 **Mass:** 60154 **Score:** 63 **Matches:** 2(2) **Sequences:** 1(1)
Krt6b protein [Mus musculus]
gi|148672084 **Mass:** 34775 **Score:** 63 **Matches:** 2(2) **Sequences:** 1(1)
mCG17577 [Mus musculus]
gi|148672085 **Mass:** 38119 **Score:** 63 **Matches:** 2(2) **Sequences:** 1(1)
mCG144996 [Mus musculus]
gi|13272554 **Mass:** 42357 **Score:** 63 **Matches:** 2(2) **Sequences:** 1(1)
cytokeratin KRT2-6HF [Mus musculus]
gi|29789317 **Mass:** 59704 **Score:** 63 **Matches:** 2(2) **Sequences:** 1(1)

keratin, type II cytoskeletal 75 [Mus musculus]
gi|148672089 **Mass:** 62241 **Score:** 63 **Matches:** 2(2) **Sequences:** 1(1)
keratin 75 [Mus musculus]

---

13. gi|5295992 **Mass:** 59531 **Score:** 63 **Matches:** 2(2) **Sequences:** 2(2) **emPAI:** 0.11
chaperonin containing TCP-1 theta subunit [Mus musculus]

Query	Observed	Mr(expt)	Mr(calc)	Delta	Miss	Score	Expect	Rank	Unique	Peptide
13522	481.9923	961.9701	961.5055	0.4646	0	52	0.0053	1	U	K.APGFAQMLK.D
15269	567.3992	1132.7838	1131.5772	1.2066	0	53	0.012	1	U	R.DVDEVSSLLR.T

Proteins matching the same set of peptides:
gi|50510319 **Mass:** 60190 **Score:** 63 **Matches:** 2(2) **Sequences:** 2(2)
mKIAA0002 protein [Mus musculus]
gi|74219231 **Mass:** 59518 **Score:** 63 **Matches:** 2(2) **Sequences:** 2(2)
unnamed protein product [Mus musculus]
gi|126723461 **Mass:** 59518 **Score:** 63 **Matches:** 2(2) **Sequences:** 2(2)
T-complex protein 1 subunit theta [Mus musculus]

---

14. gi|1419012 **Mass:** 58147 **Score:** 63 **Matches:** 1(1) **Sequences:** 1(1) **emPAI:** 0.06
CCT zeta2, zeta2 subunit of the chaperonin containing TCP-1 (CCT) [Mus musculus]

Query	Observed	Mr(expt)	Mr(calc)	Delta	Miss	Score	Expect	Rank	Unique	Peptide
13828	496.0008	989.9870	989.5216	0.4655	0	63	0.00052	1	U	K.MLVSGAGDIK.L

Proteins matching the same set of peptides:
gi|6753324 **Mass:** 57968 **Score:** 63 **Matches:** 1(1) **Sequences:** 1(1)
T-complex protein 1 subunit zeta [Mus musculus]
gi|62948125 **Mass:** 58040 **Score:** 63 **Matches:** 1(1) **Sequences:** 1(1)
Chaperonin containing Tcp1, subunit 6a (zeta) [Mus musculus]
gi|74198471 **Mass:** 58067 **Score:** 63 **Matches:** 1(1) **Sequences:** 1(1)
unnamed protein product [Mus musculus]
gi|74204475 **Mass:** 57967 **Score:** 63 **Matches:** 1(1) **Sequences:** 1(1)
unnamed protein product [Mus musculus]
gi|74204595 **Mass:** 58068 **Score:** 63 **Matches:** 1(1) **Sequences:** 1(1)
unnamed protein product [Mus musculus]
gi|226693361 **Mass:** 58148 **Score:** 63 **Matches:** 1(1) **Sequences:** 1(1)
T-complex protein 1 subunit zeta-2 [Mus musculus]

---

15. gi|7710044 **Mass:** 69508 **Score:** 60 **Matches:** 1(1) **Sequences:** 1(1) **emPAI:** 0.05
kelch-like ECH-associated protein 1 [Mus musculus]

Query	Observed	Mr(expt)	Mr(calc)	Delta	Miss	Score	Expect	Rank	Unique	Peptide
17782	691.6884	1381.3623	1381.6990	-0.3367	0	60	0.0021	1	U	R.LLYAVGGFDGTNR.L

Proteins matching the same set of peptides:
gi|26337871 **Mass:** 69478 **Score:** 60 **Matches:** 1(1) **Sequences:** 1(1)
unnamed protein product [Mus musculus]
gi|37359786 **Mass:** 71015 **Score:** 60 **Matches:** 1(1) **Sequences:** 1(1)
mKIAA0132 protein [Mus musculus]
gi|74181739 **Mass:** 69492 **Score:** 60 **Matches:** 1(1) **Sequences:** 1(1)
unnamed protein product [Mus musculus]
gi|74200263 **Mass:** 43846 **Score:** 60 **Matches:** 1(1) **Sequences:** 1(1)

unnamed protein product [Mus musculus]
gi|74212473 **Mass:** 69482 **Score:** 60 **Matches:** 1(1) **Sequences:** 1(1)
unnamed protein product [Mus musculus]
gi|93278448 **Mass:** 34652 **Score:** 60 **Matches:** 1(1) **Sequences:** 1(1)
Chain A, Structural Basis For The Defects Of Human Lung Cancer Somatic Mutations In The Repression Activity Of Keap1 On Nrf2
gi|158428176 **Mass:** 34946 **Score:** 60 **Matches:** 1(1) **Sequences:** 1(1)
Chain A, Crystal Structure Of The Keap1 Protein In Complexed With The N-Terminal Region Of The Nrf2 Transcription Factor

---

16. gi|2623222 **Mass:** 56344 **Score:** 60 **Matches:** 1(1) **Sequences:** 1(1) **emPAI:** 0.06
ATP synthase beta-subunit [Mus musculus]

Query	Observed	Mr(expt)	Mr(calc)	Delta	Miss	Score	Expect	Rank	Unique	Peptide
18179	718.5294	1435.0442	1434.7467	0.2975	0	60	0.00091	1	U	R.FTQAGSEVSALLGR.I

Proteins matching the same set of peptides:
gi|23272966 **Mass:** 56632 **Score:** 60 **Matches:** 1(1) **Sequences:** 1(1)
Atp5b protein [Mus musculus]
gi|31980648 **Mass:** 56265 **Score:** 60 **Matches:** 1(1) **Sequences:** 1(1)
ATP synthase subunit beta, mitochondrial precursor [Mus musculus]
gi|74139650 **Mass:** 56207 **Score:** 60 **Matches:** 1(1) **Sequences:** 1(1)
unnamed protein product [Mus musculus]
gi|74151565 **Mass:** 56294 **Score:** 60 **Matches:** 1(1) **Sequences:** 1(1)
unnamed protein product [Mus musculus]
gi|74185232 **Mass:** 56278 **Score:** 60 **Matches:** 1(1) **Sequences:** 1(1)
unnamed protein product [Mus musculus]
gi|74197074 **Mass:** 56294 **Score:** 60 **Matches:** 1(1) **Sequences:** 1(1)
unnamed protein product [Mus musculus]
gi|74198645 **Mass:** 56294 **Score:** 60 **Matches:** 1(1) **Sequences:** 1(1)
unnamed protein product [Mus musculus]
gi|74204509 **Mass:** 56294 **Score:** 60 **Matches:** 1(1) **Sequences:** 1(1)
unnamed protein product [Mus musculus]
gi|74220566 **Mass:** 56221 **Score:** 60 **Matches:** 1(1) **Sequences:** 1(1)
unnamed protein product [Mus musculus]
gi|74225421 **Mass:** 56235 **Score:** 60 **Matches:** 1(1) **Sequences:** 1(1)
unnamed protein product [Mus musculus]
gi|89574015 **Mass:** 48047 **Score:** 60 **Matches:** 1(1) **Sequences:** 1(1)
mitochondrial ATP synthase, H+ transporting F1 complex beta subunit [Mus musculus]

---

17. gi|192262 **Mass:** 54219 **Score:** 58 **Matches:** 1(1) **Sequences:** 1(1) **emPAI:** 0.06
pro-alpha-1 type I collagen, partial [Mus musculus]

Query	Observed	Mr(expt)	Mr(calc)	Delta	Miss	Score	Expect	Rank	Unique	Peptide
15700	589.5136	1177.0126	1176.5598	0.4528	0	58	0.0014	1	U	R.GQAGVMGFPGPK.G

Proteins matching the same set of peptides:
gi|470674 **Mass:** 137859 **Score:** 58 **Matches:** 1(1) **Sequences:** 1(1)
collagen pro-alpha-1 type I chain [Mus musculus]
gi|34328108 **Mass:** 137948 **Score:** 58 **Matches:** 1(1) **Sequences:** 1(1)
collagen alpha-1(I) chain precursor [Mus musculus]
gi|37589303 **Mass:** 117788 **Score:** 58 **Matches:** 1(1) **Sequences:** 1(1)
Col1a1 protein [Mus musculus]

---

18. gi|551295 **Mass:** 57824 **Score:** 57 **Matches:** 1(1) **Sequences:** 1(1) **emPAI:** 0.06

pyruvate kinase M [Mus musculus]

Query	Observed	Mr(expt)	Mr(calc)	Delta	Miss	Score	Expect	Rank	Unique	Peptide
18372	732.1815	1462.3485	1461.8079	0.5406	0	57	0.0032	1	U	K.IYVDDGLISLQVK.E

Proteins matching the same set of peptides:

gi\|1405933 **Mass:** 57878 **Score:** 57 **Matches:** 1(1) **Sequences:** 1(1)
M2-type pyruvate kinase [Mus musculus]
gi\|31981562 **Mass:** 57808 **Score:** 57 **Matches:** 1(1) **Sequences:** 1(1)
pyruvate kinase isozymes M1/M2 isoform 1 [Mus musculus]
gi\|74151988 **Mass:** 57820 **Score:** 57 **Matches:** 1(1) **Sequences:** 1(1)
unnamed protein product [Mus musculus]
gi\|74196318 **Mass:** 57807 **Score:** 57 **Matches:** 1(1) **Sequences:** 1(1)
unnamed protein product [Mus musculus]
gi\|74212815 **Mass:** 43138 **Score:** 57 **Matches:** 1(1) **Sequences:** 1(1)
unnamed protein product [Mus musculus]
gi\|74221210 **Mass:** 57809 **Score:** 57 **Matches:** 1(1) **Sequences:** 1(1)
unnamed protein product [Mus musculus]
gi\|359807367 **Mass:** 57948 **Score:** 57 **Matches:** 1(1) **Sequences:** 1(1)
pyruvate kinase isozymes M1/M2 isoform 2 [Mus musculus]

---

19. gi\|52789 **Mass:** 54415 **Score:** 57 **Matches:** 1(1) **Sequences:** 1(1) **emPAI:** 0.06
unnamed protein product [Mus musculus]

Query	Observed	Mr(expt)	Mr(calc)	Delta	Miss	Score	Expect	Rank	Unique	Peptide
17798	692.5499	1383.0852	1382.7194	0.3659	1	57	0.0016	1	U	K.SLNNKFASFIDK.V

Proteins matching the same set of peptides:

gi\|309215 **Mass:** 53210 **Score:** 57 **Matches:** 1(1) **Sequences:** 1(1)
EndoA' cytokeratin (5' end put.); putative [Mus musculus]
gi\|511654 **Mass:** 54220 **Score:** 57 **Matches:** 1(1) **Sequences:** 1(1)
keratin type II [Mus musculus]
gi\|74177777 **Mass:** 54546 **Score:** 57 **Matches:** 1(1) **Sequences:** 1(1)
unnamed protein product [Mus musculus]
gi\|74219975 **Mass:** 54459 **Score:** 57 **Matches:** 1(1) **Sequences:** 1(1)
unnamed protein product [Mus musculus]
gi\|76779293 **Mass:** 54514 **Score:** 57 **Matches:** 1(1) **Sequences:** 1(1)
Keratin 8 [Mus musculus]
gi\|114145561 **Mass:** 54531 **Score:** 57 **Matches:** 1(1) **Sequences:** 1(1)
keratin, type II cytoskeletal 8 [Mus musculus]

---

20. gi\|46485130 **Mass:** 85186 **Score:** 57 **Matches:** 1(1) **Sequences:** 1(1) **emPAI:** 0.04
TPA_exp: keratin Kb40 [Mus musculus]

Query	Observed	Mr(expt)	Mr(calc)	Delta	Miss	Score	Expect	Rank	Unique	Peptide
17798	692.5499	1383.0852	1382.6830	0.4022	0	57	0.0017	2	U	R.SLNNQFASFIDK.V

Proteins matching the same set of peptides:

gi\|111185567 **Score:** 57 **Matches:** 1(1) **Sequences:** 1(1)
gi\|111185722 **Score:** 57 **Matches:** 1(1) **Sequences:** 1(1)
gi\|119850791 **Score:** 57 **Matches:** 1(1) **Sequences:** 1(1)
gi\|145580629 **Mass:** 112194 **Score:** 57 **Matches:** 1(1) **Sequences:** 1(1)
keratin Kb40 [Mus musculus]

---

21. gi|1304157 **Mass:** 72412 **Score:** 57 **Matches:** 1(1) **Sequences:** 1(1) **emPAI:** 0.05
78 kDa glucose-regulated protein [Mus musculus]

Query	Observed	Mr(expt)	Mr(calc)	Delta	Miss	Score	Expect	Rank	Unique	Peptide
18139	715.6650	1429.3155	1429.6838	-0.3682	0	57	0.0043	1	U	R.TWNDPSVQQDIK.F

Proteins matching the same set of peptides:
gi|2598562 **Mass:** 72433 **Score:** 57 **Matches:** 1(1) **Sequences:** 1(1)
BiP [Mus musculus]
gi|12835845 **Mass:** 72378 **Score:** 57 **Matches:** 1(1) **Sequences:** 1(1)
unnamed protein product [Mus musculus]
gi|74143673 **Mass:** 56858 **Score:** 57 **Matches:** 1(1) **Sequences:** 1(1)
unnamed protein product [Mus musculus]
gi|74188814 **Mass:** 72305 **Score:** 57 **Matches:** 1(1) **Sequences:** 1(1)
unnamed protein product [Mus musculus]
gi|74198293 **Mass:** 72419 **Score:** 57 **Matches:** 1(1) **Sequences:** 1(1)
unnamed protein product [Mus musculus]
gi|74198974 **Mass:** 72361 **Score:** 57 **Matches:** 1(1) **Sequences:** 1(1)
unnamed protein product [Mus musculus]
gi|74207492 **Mass:** 72301 **Score:** 57 **Matches:** 1(1) **Sequences:** 1(1)
unnamed protein product [Mus musculus]
gi|74220199 **Mass:** 68455 **Score:** 57 **Matches:** 1(1) **Sequences:** 1(1)
unnamed protein product [Mus musculus]
gi|74225394 **Mass:** 72337 **Score:** 57 **Matches:** 1(1) **Sequences:** 1(1)
unnamed protein product [Mus musculus]
gi|254540166 **Mass:** 72377 **Score:** 57 **Matches:** 1(1) **Sequences:** 1(1)
78 kDa glucose-regulated protein precursor [Mus musculus]

---

22. gi|4159806 **Mass:** 65183 **Score:** 55 **Matches:** 2(2) **Sequences:** 2(2) **emPAI:** 0.10
type II keratin subunit protein [Mus musculus]

Query	Observed	Mr(expt)	Mr(calc)	Delta	Miss	Score	Expect	Rank	Unique	Peptide
17798	692.5499	1383.0852	1383.7034	-0.6182	1	44	0.031	3	U	K.SLNDKFASFIDK.V
17884	697.6450	1393.2755	1392.7249	0.5506	1	55	0.0067	1	U	R.TNAENEFVTIKK.D

Proteins matching the same set of peptides:
gi|12859782 **Mass:** 65586 **Score:** 55 **Matches:** 2(2) **Sequences:** 2(2)
unnamed protein product [Mus musculus]
gi|126116585 **Mass:** 65565 **Score:** 55 **Matches:** 2(2) **Sequences:** 2(2)
keratin, type II cytoskeletal 1 [Mus musculus]

---

23. gi|293686 **Mass:** 59414 **Score:** 55 **Matches:** 1(1) **Sequences:** 1(1) **emPAI:** 0.06
epidermal keratin subunit II [Mus musculus]

Query	Observed	Mr(expt)	Mr(calc)	Delta	Miss	Score	Expect	Rank	Unique	Peptide
17884	697.6450	1393.2755	1393.7089	-0.4334	1	55	0.0067	1	U	R.TDAENEFVTLKK.D

---

24. gi|3687320 **Mass:** 46137 **Score:** 52 **Matches:** 1(1) **Sequences:** 1(1) **emPAI:** 0.07
cartilage-associated protein (CASP) [Mus musculus]

Query	Observed	Mr(expt)	Mr(calc)	Delta	Miss	Score	Expect	Rank	Unique	Peptide
19209	840.6470	1679.2794	1678.8488	0.4306	0	52	0.01	1	U	R.TSISDMELALPDFLK.A

Proteins matching the same set of peptides:

gi|12857227 **Mass:** 46121 **Score:** 52 **Matches:** 1(1) **Sequences:** 1(1)
unnamed protein product [Mus musculus]
gi|148677365 **Mass:** 46110 **Score:** 52 **Matches:** 1(1) **Sequences:** 1(1)
mCG7119 [Mus musculus]
gi|225543173 **Mass:** 46140 **Score:** 52 **Matches:** 1(1) **Sequences:** 1(1)
cartilage-associated protein precursor [Mus musculus]

---

25. gi|74203983 **Mass:** 60183 **Score:** 51 **Matches:** 1(1) **Sequences:** 1(1) **emPAI:** 0.05
unnamed protein product [Mus musculus]

Query	Observed	Mr(expt)	Mr(calc)	Delta	Miss	Score	Expect	Rank	Unique	Peptide
17926	700.4083	1398.8021	1398.6159	0.1862	0	51	0.02	1	U	K.SIDMSWDSVTMK.H

Proteins matching the same set of peptides:
gi|76677895 **Mass:** 58508 **Score:** 51 **Matches:** 1(1) **Sequences:** 1(1)
poly(U)-binding-splicing factor PUF60 isoform b [Mus musculus]
gi|111185612 **Mass:** 26264 **Score:** 51 **Matches:** 1(1) **Sequences:** 1(1)
Puf60 protein [Mus musculus]
gi|148697575 **Mass:** 58380 **Score:** 51 **Matches:** 1(1) **Sequences:** 1(1)
RIKEN cDNA 2410104I19, isoform CRA_c [Mus musculus]
gi|148697576 **Mass:** 58982 **Score:** 51 **Matches:** 1(1) **Sequences:** 1(1)
RIKEN cDNA 2410104I19, isoform CRA_d [Mus musculus]
gi|257196183 **Mass:** 60211 **Score:** 51 **Matches:** 1(1) **Sequences:** 1(1)
poly(U)-binding-splicing factor PUF60 isoform a [Mus musculus]
gi|257196186 **Mass:** 53995 **Score:** 51 **Matches:** 1(1) **Sequences:** 1(1)
poly(U)-binding-splicing factor PUF60 isoform c [Mus musculus]

---

26. gi|468546 **Mass:** 57411 **Score:** 50 **Matches:** 1(1) **Sequences:** 1(1) **emPAI:** 0.06
CCT (chaperonin containing TCP-1) beta subunit [Mus musculus]

Query	Observed	Mr(expt)	Mr(calc)	Delta	Miss	Score	Expect	Rank	Unique	Peptide
18778	767.4490	1532.8834	1531.7916	1.0918	0	50	0.023	1	U	R.DAALMVTNDGATILK.N

Proteins matching the same set of peptides:
gi|7670405 **Mass:** 52436 **Score:** 50 **Matches:** 1(1) **Sequences:** 1(1)
unnamed protein product [Mus musculus]
gi|126521835 **Mass:** 57441 **Score:** 50 **Matches:** 1(1) **Sequences:** 1(1)
T-complex protein 1 subunit beta [Mus musculus]
gi|148689878 **Mass:** 44497 **Score:** 50 **Matches:** 1(1) **Sequences:** 1(1)
chaperonin subunit 2 (beta), isoform CRA_b [Mus musculus]

---

27. gi|22297268 **Score:** 50 **Matches:** 1(1) **Sequences:** 1(1) **emPAI:** 0.16
AID, partial [Homo sapiens]

Query	Observed	Mr(expt)	Mr(calc)	Delta	Miss	Score	Expect	Rank	Unique	Peptide
10277	381.1882	760.3618	760.3715	-0.0097	0	50	0.015	2	U	K.AESEGLR.R

---

28. gi|51450 **Mass:** 46560 **Score:** 49 **Matches:** 1(1) **Sequences:** 1(1) **emPAI:** 0.07
heat shock protein [Mus musculus]

Query	Observed	Mr(expt)	Mr(calc)	Delta	Miss	Score	Expect	Rank	Unique	Peptide
16265	612.9139	1223.8133	1223.6510	0.1623	0	49	0.027	1	U	K.GVVEVTHDLQK.H

Proteins matching the same set of peptides:

gi|200966 **Mass:** 45623 **Score:** 49 **Matches:** 1(1) **Sequences:** 1(1)
put. serine protease inhibitor [Mus musculus]
gi|26345418 **Mass:** 46481 **Score:** 49 **Matches:** 1(1) **Sequences:** 1(1)
unnamed protein product [Mus musculus]
gi|26348007 **Mass:** 46490 **Score:** 49 **Matches:** 1(1) **Sequences:** 1(1)
unnamed protein product [Mus musculus]
gi|74139266 **Mass:** 46490 **Score:** 49 **Matches:** 1(1) **Sequences:** 1(1)
unnamed protein product [Mus musculus]
gi|74191337 **Mass:** 46505 **Score:** 49 **Matches:** 1(1) **Sequences:** 1(1)
unnamed protein product [Mus musculus]
gi|74198254 **Mass:** 46500 **Score:** 49 **Matches:** 1(1) **Sequences:** 1(1)
unnamed protein product [Mus musculus]
gi|148684430 **Mass:** 44954 **Score:** 49 **Matches:** 1(1) **Sequences:** 1(1)
serine (or cysteine) peptidase inhibitor, clade H, member 1, isoform CRA_a [Mus musculus]
gi|161353502 **Mass:** 46504 **Score:** 49 **Matches:** 1(1) **Sequences:** 1(1)
serpin H1 precursor [Mus musculus]

---

29. gi|6680748 **Mass:** 59716 **Score:** 49 **Matches:** 1(1) **Sequences:** 1(1) **emPAI:** 0.06
ATP synthase subunit alpha, mitochondrial precursor [Mus musculus]

Query	Observed	Mr(expt)	Mr(calc)	Delta	Miss	Score	Expect	Rank	Unique	Peptide
9365	362.2126	722.4106	722.4439	-0.0333	0	49	0.012	1	U	K.APGIIPR.I

Proteins matching the same set of peptides:
gi|74139457 **Mass:** 59656 **Score:** 49 **Matches:** 1(1) **Sequences:** 1(1)
unnamed protein product [Mus musculus]
gi|74146998 **Mass:** 55919 **Score:** 49 **Matches:** 1(1) **Sequences:** 1(1)
unnamed protein product [Mus musculus]
gi|74182195 **Mass:** 32607 **Score:** 49 **Matches:** 1(1) **Sequences:** 1(1)
unnamed protein product [Mus musculus]
gi|74211072 **Mass:** 59730 **Score:** 49 **Matches:** 1(1) **Sequences:** 1(1)
unnamed protein product [Mus musculus]
gi|74211198 **Mass:** 51304 **Score:** 49 **Matches:** 1(1) **Sequences:** 1(1)
unnamed protein product [Mus musculus]
gi|74211977 **Mass:** 59746 **Score:** 49 **Matches:** 1(1) **Sequences:** 1(1)
unnamed protein product [Mus musculus]
gi|148677500 **Mass:** 51819 **Score:** 49 **Matches:** 1(1) **Sequences:** 1(1)
ATP synthase, H+ transporting, mitochondrial F1 complex, alpha subunit, isoform 1, isoform CRA_d [Mus musculus]
gi|148677501 **Mass:** 54561 **Score:** 49 **Matches:** 1(1) **Sequences:** 1(1)
ATP synthase, H+ transporting, mitochondrial F1 complex, alpha subunit, isoform 1, isoform CRA_e [Mus musculus]
gi|148677504 **Mass:** 54738 **Score:** 49 **Matches:** 1(1) **Sequences:** 1(1)
ATP synthase, H+ transporting, mitochondrial F1 complex, alpha subunit, isoform 1, isoform CRA_h [Mus musculus]

---

30. gi|148688671 **Score:** 49 **Matches:** 1(1) **Sequences:** 1(1) **emPAI:** 0.30
mCG16097 [Mus musculus]

Query	Observed	Mr(expt)	Mr(calc)	Delta	Miss	Score	Expect	Rank	Unique	Peptide
9365	362.2126	722.4106	722.4439	-0.0333	0	49	0.012	1	U	K.APGLLPR.L

---

31. gi|6755893 **Mass:** 26257 **Score:** 48 **Matches:** 2(2) **Sequences:** 1(1) **emPAI:** 0.27
trypsin 4 precursor [Mus musculus]

Query	Observed	Mr(expt)	Mr(calc)	Delta	Miss	Score	Expect	Rank	Unique	Peptide
18452	737.6481	2209.9224	2211.0920	-1.1696	0	47	0.039	2	U	R.LGEHNINVLEGNEQFVNSAK.I

19321	1106.1886	2210.3626	2211.0920	-0.7293	0	(47)	0.044	2	U	R.LGEHNINVLEGNEQFVNSAK.I

Proteins matching the same set of peptides:
gi|51010909 **Mass:** 26260 **Score:** 48 **Matches:** 2(2) **Sequences:** 1(1)
trypsin 5 precursor [Mus musculus]
gi|74203392 **Mass:** 27146 **Score:** 48 **Matches:** 2(2) **Sequences:** 1(1)
unnamed protein product [Mus musculus]

---

32. gi|148707748 **Score:** 47 **Matches:** 1(1) **Sequences:** 1(1) **emPAI:** 0.10
nuclear casein kinase and cyclin-dependent kinase substrate 1, isoform CRA_b [Mus musculus]

Query	Observed	Mr(expt)	Mr(calc)	Delta	Miss	Score	Expect	Rank	Unique	Peptide
18778	767.4490	1532.8834	1531.6525	1.2309	1	47	0.044	2	U	K.EEDEEAESPPEKK.S

Proteins matching the same set of peptides:
gi|224809559 **Score:** 47 **Matches:** 1(1) **Sequences:** 1(1)
gi|224809563 **Score:** 47 **Matches:** 1(1) **Sequences:** 1(1)

---

33. gi|53830774 **Mass:** 34092 **Score:** 47 **Matches:** 1(1) **Sequences:** 1(1) **emPAI:** 0.10
lactase [Mus musculus]

Query	Observed	Mr(expt)	Mr(calc)	Delta	Miss	Score	Expect	Rank	Unique	Peptide
14788	**548.6042**	**1095.1938**	**1094.5132**	**0.6806**	**0**	**47**	**0.016**	**1**	**U**	**R.LPEFTESEK.K**

Proteins matching the same set of peptides:
gi|74192292 **Mass:** 139170 **Score:** 47 **Matches:** 1(1) **Sequences:** 1(1)
unnamed protein product [Mus musculus]
gi|124487297 **Mass:** 217665 **Score:** 47 **Matches:** 1(1) **Sequences:** 1(1)
lactase-phlorizin hydrolase preproprotein [Mus musculus]
gi|148707805 **Mass:** 217693 **Score:** 47 **Matches:** 1(1) **Sequences:** 1(1)
mCG128560 [Mus musculus]

---

34. gi|26324736 **Score:** 47 **Matches:** 1(1) **Sequences:** 1(1) **emPAI:** 0.06
unnamed protein product [Mus musculus]

Query	Observed	Mr(expt)	Mr(calc)	Delta	Miss	Score	Expect	Rank	Unique	Peptide
17059	651.8716	1301.7286	1300.7238	1.0048	0	47	0.029	2	U	R.SLNLDSIIAEVK.A

Proteins matching the same set of peptides:
gi|109735018 **Score:** 47 **Matches:** 1(1) **Sequences:** 1(1)
gi|148672090 **Score:** 47 **Matches:** 1(1) **Sequences:** 1(1)
gi|269914154 **Score:** 47 **Matches:** 1(1) **Sequences:** 1(1)

---

35. gi|7513694 **Mass:** 109137 **Score:** 46 **Matches:** 1(1) **Sequences:** 1(1) **emPAI:** 0.03
IgG Fc binding protein - mouse (fragment)

Query	Observed	Mr(expt)	Mr(calc)	Delta	Miss	Score	Expect	Rank	Unique	Peptide
12529	**437.9564**	**873.8983**	**873.4920**	**0.4063**	**0**	**46**	**0.022**	**1**	**U**	**R.ISVINGGSK.A**

Proteins matching the same set of peptides:
gi|148692210 **Mass:** 147962 **Score:** 46 **Matches:** 1(1) **Sequences:** 1(1)
mCG145390, isoform CRA_a [Mus musculus]
gi|148692211 **Mass:** 146729 **Score:** 46 **Matches:** 1(1) **Sequences:** 1(1)
mCG145390, isoform CRA_b [Mus musculus]

gi|169790797 **Mass:** 275058 **Score:** 46 **Matches:** 1(1) **Sequences:** 1(1)
Fc fragment of IgG binding protein precursor [Mus musculus]
gi|257467625 **Mass:** 280044 **Score:** 46 **Matches:** 1(1) **Sequences:** 1(1)
Fc fragment of IgG binding protein-like precursor [Mus musculus]

---

36. gi|111599858 **Score:** 46 **Matches:** 1(1) **Sequences:** 1(1) **emPAI:** 0.03
Dehydrogenase E1 and transketolase domain containing 1 [Mus musculus]

Query	Observed	Mr(expt)	Mr(calc)	Delta	Miss	Score	Expect	Rank	Unique	Peptide
10277	381.1882	760.3618	759.3875	0.9743	1	46	0.034	3	U	K.AKSGEPR.G

Proteins matching the same set of peptides:
gi|124487485 **Score:** 46 **Matches:** 1(1) **Sequences:** 1(1)

---

37. gi|74224513 **Mass:** 37387 **Score:** 44 **Matches:** 1(1) **Sequences:** 1(1) **emPAI:** 0.09
unnamed protein product [Mus musculus]

Query	Observed	Mr(expt)	Mr(calc)	Delta	Miss	Score	Expect	Rank	Unique	Peptide
11939	**419.3358**	**836.6570**	**835.5280**	**1.1290**	**0**	**44**	**0.035**	**1**	**U**	**R.VAAPPLLR.V**

---

38. gi|21410133 **Score:** 44 **Matches:** 1(1) **Sequences:** 1(1) **emPAI:** 0.13
Kidins220 protein [Mus musculus]

Query	Observed	Mr(expt)	Mr(calc)	Delta	Miss	Score	Expect	Rank	Unique	Peptide
10277	381.1882	760.3618	759.3875	0.9743	1	44	0.055	4	U	K.AEGKAER.V

Proteins matching the same set of peptides:
gi|28385979 **Mass:** 172572 **Score:** 44 **Matches:** 1(1) **Sequences:** 1(1)
Kidins220 protein [Mus musculus]
gi|28972688 **Mass:** 188137 **Score:** 44 **Matches:** 1(1) **Sequences:** 1(1)
mKIAA1250 protein [Mus musculus]
gi|62826025 **Score:** 44 **Matches:** 1(1) **Sequences:** 1(1)
gi|124487039 **Mass:** 199059 **Score:** 44 **Matches:** 1(1) **Sequences:** 1(1)
kinase D-interacting substrate of 220 kDa [Mus musculus]
gi|148705033 **Mass:** 190650 **Score:** 44 **Matches:** 1(1) **Sequences:** 1(1)
mCG18896 [Mus musculus]
gi|187957160 **Mass:** 185440 **Score:** 44 **Matches:** 1(1) **Sequences:** 1(1)
Kidins220 protein [Mus musculus]

---

39. gi|26333649 **Score:** 44 **Matches:** 1(1) **Sequences:** 1(1) **emPAI:** 0.07
unnamed protein product [Mus musculus]

Query	Observed	Mr(expt)	Mr(calc)	Delta	Miss	Score	Expect	Rank	Unique	Peptide
13828	496.0008	989.9870	989.5182	0.4689	1	44	0.04	2	U	K.FPKDAGLDK.L

Proteins matching the same set of peptides:
gi|74146722 **Score:** 44 **Matches:** 1(1) **Sequences:** 1(1)
gi|74192493 **Score:** 44 **Matches:** 1(1) **Sequences:** 1(1)
gi|113865995 **Score:** 44 **Matches:** 1(1) **Sequences:** 1(1)
gi|116325981 **Score:** 44 **Matches:** 1(1) **Sequences:** 1(1)
gi|148669182 **Score:** 44 **Matches:** 1(1) **Sequences:** 1(1)
gi|148707033 **Score:** 44 **Matches:** 1(1) **Sequences:** 1(1)
gi|254847433 **Score:** 44 **Matches:** 1(1) **Sequences:** 1(1)

gi|254847435 **Score:** 44 **Matches:** 1(1) **Sequences:** 1(1)
gi|254847438 **Score:** 44 **Matches:** 1(1) **Sequences:** 1(1)
gi|377837285 **Score:** 44 **Matches:** 1(1) **Sequences:** 1(1)
gi|377837287 **Score:** 44 **Matches:** 1(1) **Sequences:** 1(1)

---

40. gi|986918 **Mass:** 51416 **Score:** 44 **Matches:** 1(1) **Sequences:** 1(1) **emPAI:** 0.06
A10 [Mus musculus]

Query	Observed	Mr(expt)	Mr(calc)	Delta	Miss	Score	Expect	Rank	Unique	Peptide
15221	566.0025	1129.9904	1129.5979	0.3925	0	44	0.036	1	U	K.VTADVINAAEK.L

Proteins matching the same set of peptides:
gi|26345686 **Mass:** 56546 **Score:** 44 **Matches:** 1(1) **Sequences:** 1(1)
unnamed protein product [Mus musculus]
gi|52353955 **Mass:** 56549 **Score:** 44 **Matches:** 1(1) **Sequences:** 1(1)
D-3-phosphoglycerate dehydrogenase [Mus musculus]

---

41. gi|21313084 **Mass:** 56107 **Score:** 44 **Matches:** 1(1) **Sequences:** 1(1) **emPAI:** 0.06
chromodomain Y-like protein 2 [Mus musculus]

Query	Observed	Mr(expt)	Mr(calc)	Delta	Miss	Score	Expect	Rank	Unique	Peptide
15639	585.4729	1168.9312	1168.6676	0.2636	1	44	0.035	1	U	K.RVNSPLSRPK.K

Proteins matching the same set of peptides:
gi|32479356 **Mass:** 56107 **Score:** 44 **Matches:** 1(1) **Sequences:** 1(1)
chromodomain Y-like protein 2 [Mus musculus]
gi|74206467 **Mass:** 56077 **Score:** 44 **Matches:** 1(1) **Sequences:** 1(1)
unnamed protein product [Mus musculus]

---

42. gi|124504325 **Mass:** 128405 **Score:** 44 **Matches:** 1(0) **Sequences:** 1(0) **emPAI:** 0.03
Armcx4 protein [Mus musculus]

Query	Observed	Mr(expt)	Mr(calc)	Delta	Miss	Score	Expect	Rank	Unique	Peptide
12706	887.4671	886.4599	886.4621	-0.0022	1	44	0.071	1	U	K.GNRNSVPK.A

Proteins matching the same set of peptides:
gi|363989837 **Mass:** 242806 **Score:** 44 **Matches:** 1(0) **Sequences:** 1(0)
armadillo repeat-containing X-linked protein 4 [Mus musculus]

---

43. gi|12843046 **Mass:** 26390 **Score:** 43 **Matches:** 1(0) **Sequences:** 1(0) **emPAI:** 0.13
unnamed protein product [Mus musculus]

Query	Observed	Mr(expt)	Mr(calc)	Delta	Miss	Score	Expect	Rank	Unique	Peptide
18063	711.1305	1420.2464	1419.7146	0.5318	0	43	0.081	1	U	K.YVNWIQQTIAAN.-

Proteins matching the same set of peptides:
gi|71043961 **Mass:** 26405 **Score:** 43 **Matches:** 1(0) **Sequences:** 1(0)
trypsinogen 7 precursor [Mus musculus]
gi|148681611 **Mass:** 25182 **Score:** 43 **Matches:** 1(0) **Sequences:** 1(0)
RIKEN cDNA 2210010C04, isoform CRA_a [Mus musculus]

---

44. gi|12836661 **Mass:** 117980 **Score:** 43 **Matches:** 1(1) **Sequences:** 1(1) **emPAI:** 0.03
unnamed protein product [Mus musculus]

Query	Observed	Mr(expt)	Mr(calc)	Delta	Miss	Score	Expect	Rank	Unique	Peptide

14417	530.9701	1059.9256	1059.5093	0.4164	0	43	0.045	1	U	R.CPPPLACALK.A

Proteins matching the same set of peptides:
gi|16741201 **Mass:** 70327 **Score:** 43 **Matches:** 1(1) **Sequences:** 1(1)
Pelp1 protein [Mus musculus]
gi|37046775 **Mass:** 91728 **Score:** 43 **Matches:** 1(1) **Sequences:** 1(1)
Pelp1 protein, partial [Mus musculus]
gi|257900472 **Mass:** 117995 **Score:** 43 **Matches:** 1(1) **Sequences:** 1(1)
proline-, glutamic acid- and leucine-rich protein 1 [Mus musculus]

---

45. gi|148681245 **Mass:** 9487 **Score:** 43 **Matches:** 1(0) **Sequences:** 1(0) **emPAI:** 0.37
mCG145199 [Mus musculus]

Query	Observed	Mr(expt)	Mr(calc)	Delta	Miss	Score	Expect	Rank	Unique	Peptide
11940	419.3534	836.6922	836.4729	0.2193	1	43	0.085	1	U	R.LAGHGRAR.S

---

46. gi|148667455 **Mass:** 58456 **Score:** 43 **Matches:** 1(1) **Sequences:** 1(1) **emPAI:** 0.06
TEA domain family member 4, isoform CRA_b [Mus musculus]

Query	Observed	Mr(expt)	Mr(calc)	Delta	Miss	Score	Expect	Rank	Unique	Peptide
14488	534.0168	1066.0190	1066.5995	-0.5805	1	43	0.041	1	U	R.ARGPAAVAAAGR.L

Proteins matching the same set of peptides:
gi|148667459 **Mass:** 62771 **Score:** 43 **Matches:** 1(1) **Sequences:** 1(1)
TEA domain family member 4, isoform CRA_f [Mus musculus]
gi|148667454 **Mass:** 59545 **Score:** 43 **Matches:** 1(1) **Sequences:** 1(1)
TEA domain family member 4, isoform CRA_a [Mus musculus]

---

47. gi|642072 **Mass:** 118421 **Score:** 43 **Matches:** 1(0) **Sequences:** 1(0) **emPAI:** 0.03
fibrillin-1, partial [Mus musculus]

Query	Observed	Mr(expt)	Mr(calc)	Delta	Miss	Score	Expect	Rank	Unique	Peptide
14550	536.8515	1071.6884	1070.4451	1.2433	0	43	0.076	1	U	R.CVNTDGSYR.C

Proteins matching the same set of peptides:
gi|726324 **Mass:** 312194 **Score:** 43 **Matches:** 1(0) **Sequences:** 1(0)
fibrillin-1 [Mus musculus]
gi|2494284 **Mass:** 312051 **Score:** 43 **Matches:** 1(0) **Sequences:** 1(0)
RecName: Full=Fibrillin-1; Flags: Precursor
gi|3688648 **Mass:** 418019 **Score:** 43 **Matches:** 1(0) **Sequences:** 1(0)
mutant fibrillin-1 [Mus musculus]
gi|118197277 **Mass:** 312083 **Score:** 43 **Matches:** 1(0) **Sequences:** 1(0)
fibrillin-1 precursor [Mus musculus]
gi|148696192 **Mass:** 312056 **Score:** 43 **Matches:** 1(0) **Sequences:** 1(0)
fibrillin 1, isoform CRA_a [Mus musculus]

---

48. gi|12836791 **Mass:** 23191 **Score:** 43 **Matches:** 1(0) **Sequences:** 1(0) **emPAI:** 0.14
unnamed protein product [Mus musculus]

Query	Observed	Mr(expt)	Mr(calc)	Delta	Miss	Score	Expect	Rank	Unique	Peptide
16636	629.0249	1256.0352	1255.5478	0.4874	0	43	0.11	1	U	-.MPPGPCAWPPR.A

Proteins matching the same set of peptides:

gi|47059145 **Mass:** 23306 **Score:** 43 **Matches:** 1(0) **Sequences:** 1(0)
fibronectin type III domain-containing protein 5 precursor [Mus musculus]

---

49. gi|14250408 **Mass:** 57081 **Score:** 42 **Matches:** 1(0) **Sequences:** 1(0) **emPAI:** 0.06
Aspartyl-tRNA synthetase [Mus musculus]

Query	Observed	Mr(expt)	Mr(calc)	Delta	Miss	Score	Expect	Rank	Unique	Peptide
15565	581.3489	1160.6833	1159.6601	1.0232	0	42	0.077	1	U	K.IYVISLAEPR.L

Proteins matching the same set of peptides:
gi|26346400 **Mass:** 57111 **Score:** 42 **Matches:** 1(0) **Sequences:** 1(0)
unnamed protein product [Mus musculus]
gi|74139282 **Mass:** 57145 **Score:** 42 **Matches:** 1(0) **Sequences:** 1(0)
unnamed protein product [Mus musculus]
gi|74141887 **Mass:** 57067 **Score:** 42 **Matches:** 1(0) **Sequences:** 1(0)
unnamed protein product [Mus musculus]
gi|74226918 **Mass:** 57109 **Score:** 42 **Matches:** 1(0) **Sequences:** 1(0)
unnamed protein product [Mus musculus]
gi|148707802 **Mass:** 64317 **Score:** 42 **Matches:** 1(0) **Sequences:** 1(0)
aspartyl-tRNA synthetase [Mus musculus]
gi|211065507 **Mass:** 57111 **Score:** 42 **Matches:** 1(0) **Sequences:** 1(0)
aspartate--tRNA ligase, cytoplasmic isoform 1 [Mus musculus]

---

50. gi|17512475 **Mass:** 46233 **Score:** 42 **Matches:** 1(0) **Sequences:** 1(0) **emPAI:** 0.07
Ankyrin repeat and SOCS box-containing 6 [Mus musculus]

Query	Observed	Mr(expt)	Mr(calc)	Delta	Miss	Score	Expect	Rank	Unique	Peptide
18446	736.9879	2207.9419	2208.1902	-0.2483	1	42	0.14	1	U	K.IQALHASLRQLESYPPPLK.H

Proteins matching the same set of peptides:
gi|19111154 **Mass:** 46247 **Score:** 42 **Matches:** 1(0) **Sequences:** 1(0)
ankyrin repeat and SOCS box protein 6 [Mus musculus]
gi|26354010 **Mass:** 46217 **Score:** 42 **Matches:** 1(0) **Sequences:** 1(0)
unnamed protein product [Mus musculus]

---

51. gi|309271872 **Mass:** 403142 **Score:** 42 **Matches:** 1(1) **Sequences:** 1(1) **emPAI:** 0.01
PREDICTED: zinc finger protein 469 [Mus musculus]

Query	Observed	Mr(expt)	Mr(calc)	Delta	Miss	Score	Expect	Rank	Unique	Peptide
14830	551.5288	1101.0431	1101.5051	-0.4620	0	42	0.054	1	U	R.SSPGQPSSSPR.L

---

52. gi|26380166 **Mass:** 13950 **Score:** 42 **Matches:** 1(1) **Sequences:** 1(1) **emPAI:** 0.24
unnamed protein product [Mus musculus]

Query	Observed	Mr(expt)	Mr(calc)	Delta	Miss	Score	Expect	Rank	Unique	Peptide
17965	703.0179	1404.0212	1404.6885	-0.6673	0	42	0.056	1	U	K.GPSEGAYDVILPR.A

Proteins matching the same set of peptides:
gi|46395891 **Mass:** 48390 **Score:** 42 **Matches:** 1(1) **Sequences:** 1(1)
RecName: Full=G-protein coupled receptor family C group 5 member C; AltName: Full=Retinoic acid-induced gene 3 protein; Short=RAIG-3; Flags: Precursor
gi|148669181 **Mass:** 48467 **Score:** 42 **Matches:** 1(1) **Sequences:** 1(1)
G protein-coupled receptor, family C, group 5, member C [Mus musculus]
gi|160333231 **Mass:** 48453 **Score:** 42 **Matches:** 1(1) **Sequences:** 1(1)
G-protein coupled receptor family C group 5 member C isoform a precursor [Mus musculus]

gi|160333233 **Mass:** 48416 **Score:** 42 **Matches:** 1(1) **Sequences:** 1(1)
G-protein coupled receptor family C group 5 member C isoform b precursor [Mus musculus]
gi|215406578 **Mass:** 11983 **Score:** 42 **Matches:** 1(1) **Sequences:** 1(1)
G protein-coupled receptor, family C, group 5, member C [Mus musculus]

Mascot: http://www.matrixscience.com/

PFHR9

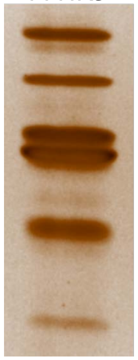

4

Mascot Search Results

```
User                    :
Email                   :
Search title            :
MS data file            : ZIEN-29Nov2017_4m.mgf
Database                : NCBInr 20120419 (17893860 sequences; 6141683785 residues)
Taxonomy                : Mus musculus (house mouse) (138831 sequences)
Timestamp               : 30 Nov 2017 at 20:51:28 GMT
Enzyme                  : Trypsin
Variable modifications  : Oxidation (M),Carbamidomethyl (C),Oxidation (P)
Mass values             : Monoisotopic
Protein Mass            : Unrestricted
Peptide Mass Tolerance  : ± 1.25 Da
Fragment Mass Tolerance : ± 1.001 Da
Max Missed Cleavages    : 1
Instrument type         : Default
Number of queries       : 16414
Protein hits            : gi|22164776  keratin, type II cytoskeletal 79 [Mus musculus]
                          gi|51450     heat shock protein [Mus musculus]
                          gi|7638398   epidermal keratin 10 [Mus musculus]
                          gi|16716569  protease, serine, 1 precursor [Mus musculus]
                          gi|12843914  unnamed protein product [Mus musculus]
                          gi|9910294   keratin, type II cytoskeletal 71 [Mus musculus]
                          gi|2498741   RecName: Full=Prolyl 4-hydroxylase subunit alpha-2; Short=4-PH alpha-2; AltName: Full=Procollagen-proline,2-oxoglutarate-4-dioxygenase subunit alpha-2; Flags: Precursc
                          gi|148672076 mCG17605, isoform CRA_b [Mus musculus]
                          gi|51092303  Try10-like trypsinogen precursor [Mus musculus]
                          gi|4159806   type II keratin subunit protein [Mus musculus]
                          gi|6166378   growth suppressor 1L [Mus musculus]
                          gi|295841496 tubulin beta 3 [Mus musculus]
                          gi|438134    DNA-directed DNA polymerase [Mus musculus]
                          gi|6680888   CD63 antigen [Mus musculus]
                          gi|202210    alpha-tubulin isotype M-alpha-2 [Mus musculus]
                          gi|3687320   cartilage-associated protein (CASP) [Mus musculus]
                          gi|85701680  keratin, type II cytoskeletal 2 oral [Mus musculus]
                          gi|21489935  keratin, type I cytoskeletal 14 [Mus musculus]
                          gi|468546    CCT (chaperonin containing TCP-1) beta subunit [Mus musculus]
                          gi|6755893   trypsin 4 precursor [Mus musculus]
                          gi|27370092  elongation factor Tu, mitochondrial isoform 1 [Mus musculus]
                          gi|293686    epidermal keratin subunit II [Mus musculus]
                          gi|3212116   prefoldin subunit 2 [Mus musculus]
                          gi|193645    glucose-regulated protein 78, partial [Mus musculus]
                          gi|53830774  lactase [Mus musculus]
                          gi|81872882  RecName: Full=Adenomatous polyposis coli protein 2
                          gi|191765    alpha-fetoprotein, partial [Mus musculus]
                          gi|54777     unnamed protein product [Mus musculus]
                          gi|26390362  unnamed protein product [Mus musculus]
```

Select Summary Report

Format As | Select Summary (protein hits) ▼ | Help

Significance threshold p< 0.05 | Max. number of hits AUTO

Standard scoring ○ MudPIT scoring ◉ | Ions score or expect cut-off 43 | Show sub-sets 0

Show pop-ups ◉ Suppress pop-ups ○ | Require bold red ☐

Re-Search | ◉ All queries ○ Unassigned ○ Below homology threshold ○ Below identity threshold

1. gi|22164776 **Mass:** 57517 **Score:** 102 **Matches:** 2(2) **Sequences:** 1(1) **emPAI:** 0.06

keratin, type II cytoskeletal 79 [Mus musculus]

Query	Observed	Mr(expt)	Mr(calc)	Delta	Miss	Score	Expect	Rank	Unique	Peptide
14241	665.9768	1329.9391	1328.7187	1.2204	0	79	1.2e-005	1	U	R.NLDLDSIIAEVK.A 14234

Proteins matching the same set of peptides:

gi|148672069 **Mass:** 59231 **Score:** 102 **Matches:** 2(2) **Sequences:** 1(1)
cDNA sequence BC031593 [Mus musculus]

---

2. gi|51450 **Mass:** 46560 **Score:** 102 **Matches:** 4(3) **Sequences:** 4(3) **emPAI:** 0.32
heat shock protein [Mus musculus]

Query	Observed	Mr(expt)	Mr(calc)	Delta	Miss	Score	Expect	Rank	Unique	Peptide
14017	653.5609	1305.1073	1305.6677	-0.5604	0	67	0.00035	1	U	R.DNQSGSLLFIGR.L
14227	665.1298	1328.2451	1327.6628	0.5823	0	52	0.013	1	U	K.LQMVEMPLAHK.L
14757	690.9507	1379.8868	1379.5881	0.2987	0	44	0.097	1	U	R.TGLYNYYDDEK.E
16290	830.6638	1659.3131	1658.7941	0.5190	0	59	0.0027	1	U	R.LYGPSSVSFADDFVR.S

Proteins matching the same set of peptides:
gi|200966 **Mass:** 45623 **Score:** 102 **Matches:** 4(3) **Sequences:** 4(3)
put. serine protease inhibitor [Mus musculus]
gi|26345418 **Mass:** 46481 **Score:** 102 **Matches:** 4(3) **Sequences:** 4(3)
unnamed protein product [Mus musculus]
gi|26348007 **Mass:** 46490 **Score:** 102 **Matches:** 4(3) **Sequences:** 4(3)
unnamed protein product [Mus musculus]
gi|74191337 **Mass:** 46505 **Score:** 102 **Matches:** 4(3) **Sequences:** 4(3)
unnamed protein product [Mus musculus]
gi|74198254 **Mass:** 46500 **Score:** 102 **Matches:** 4(3) **Sequences:** 4(3)
unnamed protein product [Mus musculus]
gi|148684430 **Mass:** 44954 **Score:** 102 **Matches:** 4(3) **Sequences:** 4(3)
serine (or cysteine) peptidase inhibitor, clade H, member 1, isoform CRA_a [Mus musculus]
gi|161353502 **Mass:** 46504 **Score:** 102 **Matches:** 4(3) **Sequences:** 4(3)
serpin H1 precursor [Mus musculus]

---

3. gi|7638398 **Mass:** 34962 **Score:** 85 **Matches:** 2(2) **Sequences:** 2(2) **emPAI:** 0.20
epidermal keratin 10 [Mus musculus]

Query	Observed	Mr(expt)	Mr(calc)	Delta	Miss	Score	Expect	Rank	Unique	Peptide
11545	546.0249	1090.0351	1089.5237	0.5115	0	46	0.023	1		K.VTMQNLNDR.L
14774	691.5409	1381.0672	1380.6408	0.4264	0	80	1.6e-005	1	U	R.ALEESNYELEGK.I

Proteins matching the same set of peptides:
gi|387397 **Mass:** 57807 **Score:** 85 **Matches:** 2(2) **Sequences:** 2(2)
epidermal keratin subunit I, partial [Mus musculus]
gi|12852157 **Mass:** 58587 **Score:** 85 **Matches:** 2(2) **Sequences:** 2(2)
unnamed protein product [Mus musculus]
gi|26349141 **Mass:** 52653 **Score:** 85 **Matches:** 2(2) **Sequences:** 2(2)
unnamed protein product [Mus musculus]
gi|26349459 **Mass:** 49471 **Score:** 85 **Matches:** 2(2) **Sequences:** 2(2)
unnamed protein product [Mus musculus]
gi|112983636 **Mass:** 57007 **Score:** 85 **Matches:** 2(2) **Sequences:** 2(2)
keratin, type I cytoskeletal 10 [Mus musculus]
gi|116242600 **Mass:** 57735 **Score:** 85 **Matches:** 2(2) **Sequences:** 2(2)
RecName: Full=Keratin, type I cytoskeletal 10; AltName: Full=56 kDa cytokeratin; AltName: Full=Cytokeratin-10; Short=CK-10; AltName: Full=Keratin, type I cytoskeletal 59 kDa; AltName: Full=Keratin-1[illegible]

---

4. gi|16716569 **Mass:** 26118 **Score:** 83 **Matches:** 2(1) **Sequences:** 1(1) **emPAI:** 0.13
protease, serine, 1 precursor [Mus musculus]

Query	Observed	Mr(expt)	Mr(calc)	Delta	Miss	Score	Expect	Rank	Unique	Peptide
15632	737.8900	2210.6482	2210.0967	0.5515	0	(44)	0.083	1	U	R.LGEHNINVLEGNEQFIDAAK.I
16412	1106.3871	2210.7596	2210.0967	0.6629	0	83	7.4e-006	1	U	R.LGEHNINVLEGNEQFIDAAK.I

---

5. gi|12843914 **Mass:** 29993 **Score:** 82 **Matches:** 1(1) **Sequences:** 1(1) **emPAI:** 0.11
unnamed protein product [Mus musculus]

Query	Observed	Mr(expt)	Mr(calc)	Delta	Miss	Score	Expect	Rank	Unique	Peptide
13984	651.6257	1301.2369	1301.7078	-0.4709	0	82	1.5e-005	1	U	R.SLDLDSIIAEVK.A

Proteins matching the same set of peptides:
gi|13272554 **Mass:** 42357 **Score:** 82 **Matches:** 1(1) **Sequences:** 1(1)
cytokeratin KRT2-6HF [Mus musculus]
gi|16303309 **Mass:** 61743 **Score:** 82 **Matches:** 1(1) **Sequences:** 1(1)
type II keratin 5 [Mus musculus]
gi|20911031 **Mass:** 61729 **Score:** 82 **Matches:** 1(1) **Sequences:** 1(1)
keratin, type II cytoskeletal 5 [Mus musculus]
gi|29789317 **Mass:** 59704 **Score:** 82 **Matches:** 1(1) **Sequences:** 1(1)
keratin, type II cytoskeletal 75 [Mus musculus]
gi|54607171 **Mass:** 59299 **Score:** 82 **Matches:** 1(1) **Sequences:** 1(1)
keratin, type II cytoskeletal 6A [Mus musculus]
gi|59798479 **Mass:** 60285 **Score:** 82 **Matches:** 1(1) **Sequences:** 1(1)
RecName: Full=Keratin, type II cytoskeletal 6B; AltName: Full=Cytokeratin-6B; Short=CK-6B; AltName: Full=Keratin-6-beta; Short=mK6-beta; AltName: Full=Keratin-6B; Short=K6B
gi|110645788 **Mass:** 60236 **Score:** 82 **Matches:** 1(1) **Sequences:** 1(1)
Krt6b protein [Mus musculus]
gi|113195684 **Mass:** 59490 **Score:** 82 **Matches:** 1(1) **Sequences:** 1(1)
keratin, type II cytoskeletal 6B [Mus musculus]
gi|116063325 **Mass:** 60154 **Score:** 82 **Matches:** 1(1) **Sequences:** 1(1)
Krt6b protein [Mus musculus]
gi|148672084 **Mass:** 34775 **Score:** 82 **Matches:** 1(1) **Sequences:** 1(1)
mCG17577 [Mus musculus]
gi|148672089 **Mass:** 62241 **Score:** 82 **Matches:** 1(1) **Sequences:** 1(1)
keratin 75 [Mus musculus]
gi|148672085 **Mass:** 38119 **Score:** 82 **Matches:** 1(1) **Sequences:** 1(1)
mCG144996 [Mus musculus]

---

6. gi|9910294 **Mass:** 57347 **Score:** 75 **Matches:** 2(2) **Sequences:** 2(2) **emPAI:** 0.12
keratin, type II cytoskeletal 71 [Mus musculus]

Query	Observed	Mr(expt)	Mr(calc)	Delta	Miss	Score	Expect	Rank	Unique	Peptide
11976	562.3885	1122.7624	1122.5557	0.2068	0	49	0.029	1		K.AEAEALYQTK.F
15642	738.2304	1474.4461	1474.7780	-0.3318	0	65	0.00053	1	U	R.FLEQQNQVLQTK.W

Proteins matching the same set of peptides:
gi|33146295 **Mass:** 57391 **Score:** 75 **Matches:** 2(2) **Sequences:** 2(2)
type II keratin [Mus musculus]
gi|148672081 **Mass:** 43429 **Score:** 75 **Matches:** 2(2) **Sequences:** 2(2)
mCG17589, isoform CRA_b [Mus musculus]

---

7. gi|2498741 **Mass:** 60964 **Score:** 66 **Matches:** 1(1) **Sequences:** 1(1) **emPAI:** 0.05
RecName: Full=Prolyl 4-hydroxylase subunit alpha-2; Short=4-PH alpha-2; AltName: Full=Procollagen-proline,2-oxoglutarate-4-dioxygenase subunit alpha-2; Flags: Precursor

Query	Observed	Mr(expt)	Mr(calc)	Delta	Miss	Score	Expect	Rank	Unique	Peptide
16157	785.1390	1568.2634	1568.6743	-0.4109	0	66	0.00051	1	U	R.QFFPTDEDESGAAR.A

Proteins matching the same set of peptides:
gi|148701596 **Mass:** 40313 **Score:** 66 **Matches:** 1(1) **Sequences:** 1(1)
procollagen-proline, 2-oxoglutarate 4-dioxygenase (proline 4-hydroxylase), alpha II polypeptide, isoform CRA_a [Mus musculus]
gi|148701597 **Mass:** 57417 **Score:** 66 **Matches:** 1(1) **Sequences:** 1(1)
procollagen-proline, 2-oxoglutarate 4-dioxygenase (proline 4-hydroxylase), alpha II polypeptide, isoform CRA_b [Mus musculus]
gi|148701599 **Mass:** 61292 **Score:** 66 **Matches:** 1(1) **Sequences:** 1(1)
procollagen-proline, 2-oxoglutarate 4-dioxygenase (proline 4-hydroxylase), alpha II polypeptide, isoform CRA_d [Mus musculus]
gi|148701600 **Mass:** 66870 **Score:** 66 **Matches:** 1(1) **Sequences:** 1(1)
procollagen-proline, 2-oxoglutarate 4-dioxygenase (proline 4-hydroxylase), alpha II polypeptide, isoform CRA_e [Mus musculus]
gi|209862961 **Mass:** 60774 **Score:** 66 **Matches:** 1(1) **Sequences:** 1(1)
prolyl 4-hydroxylase subunit alpha-2 isoform 1 precursor [Mus musculus]
gi|226874876 **Mass:** 60977 **Score:** 66 **Matches:** 1(1) **Sequences:** 1(1)
prolyl 4-hydroxylase subunit alpha-2 isoform 2 precursor [Mus musculus]

---

8. gi|148672076 **Mass:** 72140 **Score:** 65 **Matches:** 2(1) **Sequences:** 2(1) **emPAI:** 0.09

mCG17605, isoform CRA_b [Mus musculus]

Query	Observed	Mr(expt)	Mr(calc)	Delta	Miss	Score	Expect	Rank	Unique	Peptide
11007	521.2697	1040.5249	1040.5502	-0.0253	0	46	0.067	1	U	K.VDPEIQNVK.S
13545	628.0165	1254.0184	1253.6001	0.4184	0	65	0.0007	1	U	R.GFSSGSAVVSGGSR.R

Proteins matching the same set of peptides:

gi|398168 **Mass:** 70934 **Score:** 65 **Matches:** 2(1) **Sequences:** 2(1)
keratin 2 epidermis [Mus musculus]
gi|111308159 **Mass:** 70880 **Score:** 65 **Matches:** 2(1) **Sequences:** 2(1)
Keratin 2 [Mus musculus]
gi|124487419 **Mass:** 70880 **Score:** 65 **Matches:** 2(1) **Sequences:** 2(1)
keratin, type II cytoskeletal 2 epidermal [Mus musculus]
gi|148672075 **Mass:** 71783 **Score:** 65 **Matches:** 2(1) **Sequences:** 2(1)
mCG17605, isoform CRA_a [Mus musculus]

---

9. gi|51092303 **Mass:** 26514 **Score:** 65 **Matches:** 1(1) **Sequences:** 1(1) **emPAI:** 0.13
Try10-like trypsinogen precursor [Mus musculus]

Query	Observed	Mr(expt)	Mr(calc)	Delta	Miss	Score	Expect	Rank	Unique	Peptide
12639	588.3151	1174.6156	1174.6267	-0.0112	0	65	0.00087	1	U	K.TLDNDIMLIK.L

Proteins matching the same set of peptides:

gi|84781771 **Mass:** 26204 **Score:** 65 **Matches:** 1(1) **Sequences:** 1(1)
trypsin 10 precursor [Mus musculus]

---

10. gi|4159806 **Mass:** 65183 **Score:** 64 **Matches:** 1(1) **Sequences:** 1(1) **emPAI:** 0.05
type II keratin subunit protein [Mus musculus]

Query	Observed	Mr(expt)	Mr(calc)	Delta	Miss	Score	Expect	Rank	Unique	Peptide
15642	738.2304	1474.4461	1474.8144	-0.3682	1	64	0.0007	2	U	R.FLEQQNKVLQTK.W

---

11. gi|6166378 **Mass:** 84758 **Score:** 58 **Matches:** 1(1) **Sequences:** 1(1) **emPAI:** 0.04
growth suppressor 1L [Mus musculus]

Query	Observed	Mr(expt)	Mr(calc)	Delta	Miss	Score	Expect	Rank	Unique	Peptide
15783	747.1336	1492.2527	1491.7398	0.5129	0	58	0.0013	1	U	R.SPYNYLQVAYFK.I

Proteins matching the same set of peptides:

gi|6166380 **Mass:** 61560 **Score:** 58 **Matches:** 1(1) **Sequences:** 1(1)
growth suppressor 1S [Mus musculus]
gi|12846125 **Mass:** 84033 **Score:** 58 **Matches:** 1(1) **Sequences:** 1(1)
unnamed protein product [Mus musculus]
gi|23271416 **Mass:** 83540 **Score:** 58 **Matches:** 1(1) **Sequences:** 1(1)
Leprecan 1 [Mus musculus]
gi|26326437 **Mass:** 83476 **Score:** 58 **Matches:** 1(1) **Sequences:** 1(1)
unnamed protein product [Mus musculus]
gi|74205527 **Mass:** 84439 **Score:** 58 **Matches:** 1(1) **Sequences:** 1(1)
unnamed protein product [Mus musculus]
gi|109150433 **Mass:** 83598 **Score:** 58 **Matches:** 1(1) **Sequences:** 1(1)
prolyl 3-hydroxylase 1 isoform 2 precursor [Mus musculus]
gi|109150437 **Mass:** 84426 **Score:** 58 **Matches:** 1(1) **Sequences:** 1(1)
prolyl 3-hydroxylase 1 isoform 1 precursor [Mus musculus]
gi|148698509 **Mass:** 84378 **Score:** 58 **Matches:** 1(1) **Sequences:** 1(1)
leprecan 1, isoform CRA_a [Mus musculus]
gi|148698510 **Mass:** 83550 **Score:** 58 **Matches:** 1(1) **Sequences:** 1(1)
leprecan 1, isoform CRA_b [Mus musculus]
gi|148698511 **Mass:** 63605 **Score:** 58 **Matches:** 1(1) **Sequences:** 1(1)
leprecan 1, isoform CRA_c [Mus musculus]

---

12. gi|295841496 **Mass:** 14518 **Score:** 58 **Matches:** 2(2) **Sequences:** 1(1) **emPAI:** 0.23

tubulin beta 3 [Mus musculus]

Query	Observed	Mr(expt)	Mr(calc)	Delta	Miss	Score	Expect	Rank	Unique	Peptide
14141	660.4166	1318.8186	1318.6955	0.1231	0	52	0.016	1	U	R.IMNTFSVVPSPK.V 14142

Proteins matching the same set of peptides:

gi|202229 **Mass:** 42212 **Score:** 58 **Matches:** 2(2) **Sequences:** 1(1)
beta-tubulin, partial [Mus musculus]
gi|5174735 **Mass:** 49799 **Score:** 58 **Matches:** 2(2) **Sequences:** 1(1)
tubulin beta-4B chain [Homo sapiens]
gi|7106439 **Mass:** 49639 **Score:** 58 **Matches:** 2(2) **Sequences:** 1(1)
tubulin beta-5 chain [Mus musculus]
gi|12846758 **Mass:** 49608 **Score:** 58 **Matches:** 2(2) **Sequences:** 1(1)
unnamed protein product [Mus musculus]
gi|12851187 **Mass:** 49496 **Score:** 58 **Matches:** 2(2) **Sequences:** 1(1)
unnamed protein product [Mus musculus]
gi|12963615 **Mass:** 50386 **Score:** 58 **Matches:** 2(2) **Sequences:** 1(1)
tubulin beta-3 chain [Mus musculus]
gi|13542680 **Mass:** 49783 **Score:** 58 **Matches:** 2(2) **Sequences:** 1(1)
Tubulin, beta 2C [Mus musculus]
gi|21361322 **Mass:** 49554 **Score:** 58 **Matches:** 2(2) **Sequences:** 1(1)
tubulin beta-4A chain [Homo sapiens]
gi|26355849 **Mass:** 32238 **Score:** 58 **Matches:** 2(2) **Sequences:** 1(1)
unnamed protein product [Mus musculus]
gi|26356799 **Mass:** 34123 **Score:** 58 **Matches:** 2(2) **Sequences:** 1(1)
unnamed protein product [Mus musculus]
gi|74141821 **Mass:** 49667 **Score:** 58 **Matches:** 2(2) **Sequences:** 1(1)
unnamed protein product [Mus musculus]
gi|74144588 **Mass:** 49771 **Score:** 58 **Matches:** 2(2) **Sequences:** 1(1)
unnamed protein product [Mus musculus]
gi|74204140 **Mass:** 49616 **Score:** 58 **Matches:** 2(2) **Sequences:** 1(1)
unnamed protein product [Mus musculus]
gi|74212109 **Mass:** 49620 **Score:** 58 **Matches:** 2(2) **Sequences:** 1(1)
unnamed protein product [Mus musculus]
gi|74223737 **Mass:** 49652 **Score:** 58 **Matches:** 2(2) **Sequences:** 1(1)
unnamed protein product [Mus musculus]
gi|74223783 **Mass:** 50402 **Score:** 58 **Matches:** 2(2) **Sequences:** 1(1)
unnamed protein product [Mus musculus]
gi|148676266 **Mass:** 39342 **Score:** 58 **Matches:** 2(2) **Sequences:** 1(1)
mCG20287 [Mus musculus]
gi|148678613 **Mass:** 49970 **Score:** 58 **Matches:** 2(2) **Sequences:** 1(1)
mCG1424 [Mus musculus]
gi|148682710 **Mass:** 49784 **Score:** 58 **Matches:** 2(2) **Sequences:** 1(1)
mCG49614, isoform CRA_b [Mus musculus]
gi|148691289 **Mass:** 49910 **Score:** 58 **Matches:** 2(2) **Sequences:** 1(1)
tubulin, beta 5 [Mus musculus]

---

13. gi|438134 **Mass:** 123705 **Score:** 57 **Matches:** 1(1) **Sequences:** 1(1) **emPAI:** 0.03
DNA-directed DNA polymerase [Mus musculus]

Query	Observed	Mr(expt)	Mr(calc)	Delta	Miss	Score	Expect	Rank	Unique	Peptide
11720	553.2844	1104.5543	1104.6040	-0.0497	1	57	0.0047	1	U	R.RQGPGPGVPPK.R

Proteins matching the same set of peptides:

gi|2827903 **Mass:** 123657 **Score:** 57 **Matches:** 1(1) **Sequences:** 1(1)
DNA polymerase delta catalytic subunit [Mus musculus]
gi|14318657 **Mass:** 123666 **Score:** 57 **Matches:** 1(1) **Sequences:** 1(1)
Polymerase (DNA directed), delta 1, catalytic subunit [Mus musculus]
gi|26353290 **Mass:** 123698 **Score:** 57 **Matches:** 1(1) **Sequences:** 1(1)
unnamed protein product [Mus musculus]

gi|148690782 **Mass:** 125906 **Score:** 57 **Matches:** 1(1) **Sequences:** 1(1)
polymerase (DNA directed), delta 1, catalytic subunit, isoform CRA_a [Mus musculus]
gi|148690783 **Mass:** 90830 **Score:** 57 **Matches:** 1(1) **Sequences:** 1(1)
polymerase (DNA directed), delta 1, catalytic subunit, isoform CRA_b [Mus musculus]
gi|148690785 **Mass:** 122966 **Score:** 57 **Matches:** 1(1) **Sequences:** 1(1)
polymerase (DNA directed), delta 1, catalytic subunit, isoform CRA_d [Mus musculus]
gi|148690786 **Mass:** 123682 **Score:** 57 **Matches:** 1(1) **Sequences:** 1(1)
polymerase (DNA directed), delta 1, catalytic subunit, isoform CRA_e [Mus musculus]
gi|148690787 **Mass:** 107166 **Score:** 57 **Matches:** 1(1) **Sequences:** 1(1)
polymerase (DNA directed), delta 1, catalytic subunit, isoform CRA_f [Mus musculus]
gi|254587977 **Mass:** 123712 **Score:** 57 **Matches:** 1(1) **Sequences:** 1(1)
DNA polymerase delta catalytic subunit [Mus musculus]

---

14. gi|6680888 **Mass:** 25749 **Score:** 56 **Matches:** 1(1) **Sequences:** 1(1) **emPAI:** 0.13
CD63 antigen [Mus musculus]

Query	Observed	Mr(expt)	Mr(calc)	Delta	Miss	Score	Expect	Rank	Unique	Peptide
15128	708.0230	1414.0315	1413.6711	0.3604	0	56	0.0049	1	U	K.SFQQQMQNYLK.D

Proteins matching the same set of peptides:
gi|26389974 **Mass:** 26766 **Score:** 56 **Matches:** 1(1) **Sequences:** 1(1)
unnamed protein product [Mus musculus]
gi|74151897 **Mass:** 25777 **Score:** 56 **Matches:** 1(1) **Sequences:** 1(1)
unnamed protein product [Mus musculus]

---

15. gi|202210 **Mass:** 50134 **Score:** 55 **Matches:** 1(1) **Sequences:** 1(1) **emPAI:** 0.07
alpha-tubulin isotype M-alpha-2 [Mus musculus]

Query	Observed	Mr(expt)	Mr(calc)	Delta	Miss	Score	Expect	Rank	Unique	Peptide
10750	508.1963	1014.3781	1014.5709	-0.1928	0	55	0.004	1	U	K.DVNAAIATIK.T

Proteins matching the same set of peptides:
gi|202223 **Mass:** 22209 **Score:** 55 **Matches:** 1(1) **Sequences:** 1(1)
alpha-tubulin, partial [Mus musculus]
gi|202225 **Mass:** 39293 **Score:** 55 **Matches:** 1(1) **Sequences:** 1(1)
alpha-tubulin, partial [Mus musculus]
gi|6678465 **Mass:** 49928 **Score:** 55 **Matches:** 1(1) **Sequences:** 1(1)
tubulin alpha-3 chain [Mus musculus]
gi|6678469 **Mass:** 49877 **Score:** 55 **Matches:** 1(1) **Sequences:** 1(1)
tubulin alpha-1C chain [Mus musculus]
gi|6755901 **Mass:** 50104 **Score:** 55 **Matches:** 1(1) **Sequences:** 1(1)
tubulin alpha-1A chain [Mus musculus]
gi|12839396 **Mass:** 44021 **Score:** 55 **Matches:** 1(1) **Sequences:** 1(1)
unnamed protein product [Mus musculus]
gi|12850141 **Mass:** 38332 **Score:** 55 **Matches:** 1(1) **Sequences:** 1(1)
unnamed protein product [Mus musculus]
gi|13435888 **Mass:** 38746 **Score:** 55 **Matches:** 1(1) **Sequences:** 1(1)
Tuba1b protein [Mus musculus]
gi|34740335 **Mass:** 50120 **Score:** 55 **Matches:** 1(1) **Sequences:** 1(1)
tubulin alpha-1B chain [Mus musculus]
gi|53733821 **Mass:** 50120 **Score:** 55 **Matches:** 1(1) **Sequences:** 1(1)
Tubulin, alpha 1A [Mus musculus]
gi|74181454 **Mass:** 50104 **Score:** 55 **Matches:** 1(1) **Sequences:** 1(1)
unnamed protein product [Mus musculus]
gi|74186501 **Mass:** 50108 **Score:** 55 **Matches:** 1(1) **Sequences:** 1(1)
unnamed protein product [Mus musculus]
gi|74198443 **Mass:** 49907 **Score:** 55 **Matches:** 1(1) **Sequences:** 1(1)
unnamed protein product [Mus musculus]
gi|74198980 **Mass:** 50048 **Score:** 55 **Matches:** 1(1) **Sequences:** 1(1)
unnamed protein product [Mus musculus]
gi|74216624 **Mass:** 50116 **Score:** 55 **Matches:** 1(1) **Sequences:** 1(1)

unnamed protein product [Mus musculus]
gi|74220042 **Mass:** 50105 **Score:** 55 **Matches:** 1(1) **Sequences:** 1(1)
unnamed protein product [Mus musculus]
gi|148672205 **Mass:** 50888 **Score:** 55 **Matches:** 1(1) **Sequences:** 1(1)
mCG18413, isoform CRA_b [Mus musculus]
gi|148704842 **Mass:** 43956 **Score:** 55 **Matches:** 1(1) **Sequences:** 1(1)
mCG125663 [Mus musculus]

---

16. gi|3687320 **Mass:** 46137 **Score:** 54 **Matches:** 1(1) **Sequences:** 1(1) **emPAI:** 0.07
cartilage-associated protein (CASP) [Mus musculus]

Query	Observed	Mr(expt)	Mr(calc)	Delta	Miss	Score	Expect	Rank	Unique	Peptide
11191	**532.0100**	**1062.0053**	**1062.5458**	**-0.5405**	**0**	**54**	**0.0083**	**1**	**U**	**R.SVLADFQQR.E**

Proteins matching the same set of peptides:
gi|12857227 **Mass:** 46121 **Score:** 54 **Matches:** 1(1) **Sequences:** 1(1)
unnamed protein product [Mus musculus]
gi|148677365 **Mass:** 46110 **Score:** 54 **Matches:** 1(1) **Sequences:** 1(1)
mCG7119 [Mus musculus]
gi|225543173 **Mass:** 46140 **Score:** 54 **Matches:** 1(1) **Sequences:** 1(1)
cartilage-associated protein precursor [Mus musculus]

---

17. gi|85701680 **Mass:** 62806 **Score:** 53 **Matches:** 2(2) **Sequences:** 2(2) **emPAI:** 0.11
keratin, type II cytoskeletal 2 oral [Mus musculus]

Query	Observed	Mr(expt)	Mr(calc)	Delta	Miss	Score	Expect	Rank	Unique	Peptide
11227	**1065.4674**	**1064.4601**	**1064.5138**	**-0.0537**	**0**	**43**	**0.058**	**1**	**U**	**K.AQYEDIAQK.S**
11976	562.3885	1122.7624	1122.5557	0.2068	0	49	0.029	1		K.AEAEALYQTK.L

Proteins matching the same set of peptides:
gi|148672072 **Mass:** 63519 **Score:** 53 **Matches:** 2(2) **Sequences:** 2(2)
mCG144546 [Mus musculus]

---

18. gi|21489935 **Mass:** 52834 **Score:** 52 **Matches:** 2(2) **Sequences:** 2(2) **emPAI:** 0.13
keratin, type I cytoskeletal 14 [Mus musculus]

Query	Observed	Mr(expt)	Mr(calc)	Delta	Miss	Score	Expect	Rank	Unique	Peptide
10856	**515.0688**	**1028.1230**	**1028.5866**	**-0.4635**	**0**	**45**	**0.03**	**1**	**U**	**R.VLDELTLAR.A**
11545	546.0249	1090.0351	1089.5237	0.5115	0	46	0.023	1		K.VTMQNLNDR.L

Proteins matching the same set of peptides:
gi|148670626 **Mass:** 54668 **Score:** 52 **Matches:** 2(2) **Sequences:** 2(2)
mCG144006 [Mus musculus]
gi|741022 **Mass:** 49086 **Score:** 52 **Matches:** 2(2) **Sequences:** 2(2)
keratin 15
gi|904215 **Mass:** 49129 **Score:** 52 **Matches:** 2(2) **Sequences:** 2(2)
cytokeratin 15 [Mus musculus]
gi|83304265 **Mass:** 49107 **Score:** 52 **Matches:** 2(2) **Sequences:** 2(2)
RecName: Full=Keratin, type I cytoskeletal 15; AltName: Full=Cytokeratin-15; Short=CK-15; AltName: Full=Keratin-15; Short=K15
gi|226823220 **Mass:** 49463 **Score:** 52 **Matches:** 2(2) **Sequences:** 2(2)
keratin, type I cytoskeletal 15 [Mus musculus]

---

19. gi|468546 **Mass:** 57411 **Score:** 51 **Matches:** 1(1) **Sequences:** 1(1) **emPAI:** 0.06
CCT (chaperonin containing TCP-1) beta subunit [Mus musculus]

Query	Observed	Mr(expt)	Mr(calc)	Delta	Miss	Score	Expect	Rank	Unique	Peptide
16188	**791.9488**	**1581.8830**	**1581.9090**	**-0.0259**	**0**	**51**	**0.017**	**1**	**U**	**R.QVLLSAAEAAEVILR.V**

Proteins matching the same set of peptides:
gi|7670405 **Mass:** 52436 **Score:** 51 **Matches:** 1(1) **Sequences:** 1(1)
unnamed protein product [Mus musculus]

gi|126521835 **Mass:** 57441 **Score:** 51 **Matches:** 1(1) **Sequences:** 1(1)
T-complex protein 1 subunit beta [Mus musculus]

---

20. gi|6755893 **Score:** 50 **Matches:** 1(1) **Sequences:** 1(1) **emPAI:** 0.12
trypsin 4 precursor [Mus musculus]

Query	Observed	Mr(expt)	Mr(calc)	Delta	Miss	Score	Expect	Rank	Unique	Peptide
16412	1106.3871	2210.7596	2211.0920	-0.3323	0	50	0.017	2	U	R.LGEHNINVLEGNEQFVNSAK.I

Proteins matching the same set of peptides:
gi|51010909 **Score:** 50 **Matches:** 1(1) **Sequences:** 1(1)
gi|74203392 **Score:** 50 **Matches:** 1(1) **Sequences:** 1(1)

---

21. gi|27370092 **Mass:** 49477 **Score:** 50 **Matches:** 1(1) **Sequences:** 1(1) **emPAI:** 0.07
elongation factor Tu, mitochondrial isoform 1 [Mus musculus]

Query	Observed	Mr(expt)	Mr(calc)	Delta	Miss	Score	Expect	Rank	Unique	Peptide
12768	**593.2388**	**1184.4630**	**1184.6149**	**-0.1519**	**0**	**50**	**0.027**	**1**	**U**	**R.AEAGDNLGALVR.G**

Proteins matching the same set of peptides:
gi|148685430 **Mass:** 43302 **Score:** 50 **Matches:** 1(1) **Sequences:** 1(1)
mCG22399, isoform CRA_e [Mus musculus]
gi|148696761 **Mass:** 16047 **Score:** 50 **Matches:** 1(1) **Sequences:** 1(1)
mCG1048875, isoform CRA_a [Mus musculus]
gi|148696762 **Mass:** 12433 **Score:** 50 **Matches:** 1(1) **Sequences:** 1(1)
mCG1048875, isoform CRA_b [Mus musculus]
gi|148696763 **Mass:** 49507 **Score:** 50 **Matches:** 1(1) **Sequences:** 1(1)
mCG1048875, isoform CRA_c [Mus musculus]
gi|254911131 **Mass:** 47193 **Score:** 50 **Matches:** 1(1) **Sequences:** 1(1)
elongation factor Tu, mitochondrial isoform 2 [Mus musculus]
gi|148685429 **Mass:** 49613 **Score:** 50 **Matches:** 1(1) **Sequences:** 1(1)
mCG22399, isoform CRA_d [Mus musculus]

---

22. gi|293686 **Mass:** 59414 **Score:** 49 **Matches:** 1(1) **Sequences:** 1(1) **emPAI:** 0.06
epidermal keratin subunit II [Mus musculus]

Query	Observed	Mr(expt)	Mr(calc)	Delta	Miss	Score	Expect	Rank	Unique	Peptide
14931	**698.0006**	**1393.9867**	**1393.7089**	**0.2778**	**1**	**49**	**0.025**	**1**	**U**	**R.TDAENEFVTLKK.D**

---

23. gi|3212116 **Mass:** 16766 **Score:** 49 **Matches:** 1(1) **Sequences:** 1(1) **emPAI:** 0.20
prefoldin subunit 2 [Mus musculus]

Query	Observed	Mr(expt)	Mr(calc)	Delta	Miss	Score	Expect	Rank	Unique	Peptide
14659	**686.9688**	**1371.9231**	**1370.7769**	**1.1462**	**0**	**49**	**0.014**	**1**	**U**	**K.IIETLSQQLQAK.G**

Proteins matching the same set of peptides:
gi|31981577 **Mass:** 16524 **Score:** 49 **Matches:** 1(1) **Sequences:** 1(1)
prefoldin subunit 2 [Mus musculus]
gi|148707143 **Mass:** 11754 **Score:** 49 **Matches:** 1(1) **Sequences:** 1(1)
prefoldin 2, isoform CRA_b [Mus musculus]

---

24. gi|193645 **Mass:** 18932 **Score:** 46 **Matches:** 1(1) **Sequences:** 1(1) **emPAI:** 0.18
glucose-regulated protein 78, partial [Mus musculus]

Query	Observed	Mr(expt)	Mr(calc)	Delta	Miss	Score	Expect	Rank	Unique	Peptide
14961	**699.5220**	**1397.0295**	**1396.7813**	**0.2482**	**0**	**46**	**0.041**	**1**	**U**	**K.ELEEIVQPIISK.L**

Proteins matching the same set of peptides:
gi|387113 **Mass:** 16164 **Score:** 46 **Matches:** 1(1) **Sequences:** 1(1)
immunoglobulin heavy chain binding protein, partial [Mus musculus]
gi|1304157 **Mass:** 72412 **Score:** 46 **Matches:** 1(1) **Sequences:** 1(1)
78 kDa glucose-regulated protein [Mus musculus]

gi|2598562 **Mass:** 72433 **Score:** 46 **Matches:** 1(1) **Sequences:** 1(1)
BiP [Mus musculus]
gi|12835845 **Mass:** 72378 **Score:** 46 **Matches:** 1(1) **Sequences:** 1(1)
unnamed protein product [Mus musculus]
gi|74188814 **Mass:** 72305 **Score:** 46 **Matches:** 1(1) **Sequences:** 1(1)
unnamed protein product [Mus musculus]
gi|74198293 **Mass:** 72419 **Score:** 46 **Matches:** 1(1) **Sequences:** 1(1)
unnamed protein product [Mus musculus]
gi|74198974 **Mass:** 72361 **Score:** 46 **Matches:** 1(1) **Sequences:** 1(1)
unnamed protein product [Mus musculus]
gi|74207492 **Mass:** 72301 **Score:** 46 **Matches:** 1(1) **Sequences:** 1(1)
unnamed protein product [Mus musculus]
gi|74225394 **Mass:** 72337 **Score:** 46 **Matches:** 1(1) **Sequences:** 1(1)
unnamed protein product [Mus musculus]
gi|148676670 **Mass:** 56257 **Score:** 46 **Matches:** 1(1) **Sequences:** 1(1)
heat shock 70kD protein 5 (glucose-regulated protein), isoform CRA_b [Mus musculus]
gi|254540166 **Mass:** 72377 **Score:** 46 **Matches:** 1(1) **Sequences:** 1(1)
78 kDa glucose-regulated protein precursor [Mus musculus]

---

25. gi|53830774 **Mass:** 34092 **Score:** 46 **Matches:** 1(1) **Sequences:** 1(1) **emPAI:** 0.10
lactase [Mus musculus]

Query	Observed	Mr(expt)	Mr(calc)	Delta	Miss	Score	Expect	Rank	Unique	Peptide
11588	548.0338	1094.0531	1094.5132	-0.4600	0	46	0.018	1	U	R.LPEFTESEK.K

Proteins matching the same set of peptides:
gi|74192292 **Mass:** 139170 **Score:** 46 **Matches:** 1(1) **Sequences:** 1(1)
unnamed protein product [Mus musculus]
gi|124487297 **Mass:** 217665 **Score:** 46 **Matches:** 1(1) **Sequences:** 1(1)
lactase-phlorizin hydrolase preproprotein [Mus musculus]
gi|148707805 **Mass:** 217693 **Score:** 46 **Matches:** 1(1) **Sequences:** 1(1)
mCG128560 [Mus musculus]

---

26. gi|81872882 **Mass:** 242989 **Score:** 46 **Matches:** 1(1) **Sequences:** 1(1) **emPAI:** 0.01
RecName: Full=Adenomatous polyposis coli protein 2

Query	Observed	Mr(expt)	Mr(calc)	Delta	Miss	Score	Expect	Rank	Unique	Peptide
15985	762.1919	2283.5538	2284.1344	-0.5806	1	46	0.042	1	U	K.YQAAAMAVSPGTCVPSLYVRK.Q

Proteins matching the same set of peptides:
gi|117938322 **Mass:** 242963 **Score:** 46 **Matches:** 1(1) **Sequences:** 1(1)
adenomatous polyposis coli protein 2 [Mus musculus]

---

27. gi|191765 **Mass:** 47195 **Score:** 45 **Matches:** 1(0) **Sequences:** 1(0) **emPAI:** 0.07
alpha-fetoprotein, partial [Mus musculus]

Query	Observed	Mr(expt)	Mr(calc)	Delta	Miss	Score	Expect	Rank	Unique	Peptide
15680	740.7523	1479.4900	1478.7881	0.7018	0	45	0.065	1	U	K.LGEYGFQNAILVR.Y

Proteins matching the same set of peptides:
gi|26340966 **Mass:** 68678 **Score:** 45 **Matches:** 1(0) **Sequences:** 1(0)
unnamed protein product [Mus musculus]
gi|26341396 **Mass:** 64961 **Score:** 45 **Matches:** 1(0) **Sequences:** 1(0)
unnamed protein product [Mus musculus]
gi|74137565 **Mass:** 68688 **Score:** 45 **Matches:** 1(0) **Sequences:** 1(0)
unnamed protein product [Mus musculus]
gi|163310765 **Mass:** 68648 **Score:** 45 **Matches:** 1(0) **Sequences:** 1(0)
serum albumin precursor [Mus musculus]

---

28. gi|54777 **Mass:** 57108 **Score:** 45 **Matches:** 1(0) **Sequences:** 1(0) **emPAI:** 0.06
unnamed protein product [Mus musculus]

Query	Observed	Mr(expt)	Mr(calc)	Delta	Miss	Score	Expect	Rank	Unique	Peptide
13901	647.1276	1292.2406	1292.5918	-0.3512	0	45	0.055	1	U	K.MDSTANEVEAVK.V

Proteins matching the same set of peptides:

gi|42415475 **Mass:** 57023 **Score:** 45 **Matches:** 1(0) **Sequences:** 1(0)
protein disulfide-isomerase precursor [Mus musculus]
gi|74138891 **Mass:** 56951 **Score:** 45 **Matches:** 1(0) **Sequences:** 1(0)
unnamed protein product [Mus musculus]
gi|74141920 **Mass:** 57053 **Score:** 45 **Matches:** 1(0) **Sequences:** 1(0)
unnamed protein product [Mus musculus]
gi|74178069 **Mass:** 57099 **Score:** 45 **Matches:** 1(0) **Sequences:** 1(0)
unnamed protein product [Mus musculus]
gi|74190076 **Mass:** 56963 **Score:** 45 **Matches:** 1(0) **Sequences:** 1(0)
unnamed protein product [Mus musculus]
gi|74191500 **Mass:** 57022 **Score:** 45 **Matches:** 1(0) **Sequences:** 1(0)
unnamed protein product [Mus musculus]
gi|74198312 **Mass:** 57051 **Score:** 45 **Matches:** 1(0) **Sequences:** 1(0)
unnamed protein product [Mus musculus]
gi|74198632 **Mass:** 16308 **Score:** 45 **Matches:** 1(0) **Sequences:** 1(0)
unnamed protein product [Mus musculus]
gi|74198706 **Mass:** 57000 **Score:** 45 **Matches:** 1(0) **Sequences:** 1(0)
unnamed protein product [Mus musculus]
gi|74203945 **Mass:** 56566 **Score:** 45 **Matches:** 1(0) **Sequences:** 1(0)
unnamed protein product [Mus musculus]
gi|74212231 **Mass:** 57053 **Score:** 45 **Matches:** 1(0) **Sequences:** 1(0)
unnamed protein product [Mus musculus]
gi|74219772 **Mass:** 57008 **Score:** 45 **Matches:** 1(0) **Sequences:** 1(0)
unnamed protein product [Mus musculus]
gi|74220649 **Mass:** 57067 **Score:** 45 **Matches:** 1(0) **Sequences:** 1(0)
unnamed protein product [Mus musculus]
gi|148702818 **Mass:** 58984 **Score:** 45 **Matches:** 1(0) **Sequences:** 1(0)
prolyl 4-hydroxylase, beta polypeptide, isoform CRA_a [Mus musculus]
gi|148702819 **Mass:** 61492 **Score:** 45 **Matches:** 1(0) **Sequences:** 1(0)
prolyl 4-hydroxylase, beta polypeptide, isoform CRA_b [Mus musculus]

---

29. gi|26390362 **Mass:** 39663 **Score:** 45 **Matches:** 1(0) **Sequences:** 1(0) **emPAI:** 0.08
unnamed protein product [Mus musculus]

Query	Observed	Mr(expt)	Mr(calc)	Delta	Miss	Score	Expect	Rank	Unique	Peptide
12452	581.0289	1160.0432	1159.5622	0.4810	0	45	0.068	1	U	R.EGWTVQDVAR.V

Proteins matching the same set of peptides:

gi|148287013 **Mass:** 39693 **Score:** 45 **Matches:** 1(0) **Sequences:** 1(0)
paraneoplastic antigen Ma1 homolog [Mus musculus]

Mascot: http://www.matrixscience.com/

PFHR9

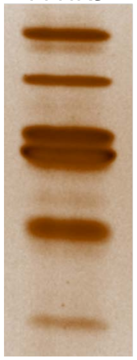

5

Mascot Search Results

User :
Email :
Search title :
MS data file : ZIEN-29Nov2017_5m.mgf
Database : NCBInr 20120419 (17893860 sequences; 6141683785 residues)
Taxonomy : Mus musculus (house mouse) (138831 sequences)
Timestamp : 30 Nov 2017 at 21:31:00 GMT
Enzyme : Trypsin
Variable modifications : Carbamidomethyl (C),Oxidation (M),Oxidation (P)
Mass values : Monoisotopic
Protein Mass : Unrestricted
Peptide Mass Tolerance : ± 1.25 Da
Fragment Mass Tolerance: ± 1.001 Da
Max Missed Cleavages : 1
Instrument type : Default
Number of queries : 15860
Protein hits : gi|4159806 type II keratin subunit protein [Mus musculus]
gi|46485130 TPA_exp: keratin Kb40 [Mus musculus]
gi|12859782 unnamed protein product [Mus musculus]
gi|47523977 keratin, type II cytoskeletal 72 [Mus musculus]
gi|387397 epidermal keratin subunit I, partial [Mus musculus]
gi|2498741 RecName: Full=Prolyl 4-hydroxylase subunit alpha-2; Short=4-PH alpha-2; AltName: Full=Procollagen-proline,2-oxoglutarate-4-dioxygenase subunit alpha-2; Flags: Precursor
gi|16303309 type II keratin 5 [Mus musculus]
gi|1389682 plakoglobin, partial [Mus musculus]
gi|16716569 protease, serine, 1 precursor [Mus musculus]
gi|26337253 unnamed protein product [Mus musculus]
gi|12846468 unnamed protein product [Mus musculus]
gi|6755893 trypsin 4 precursor [Mus musculus]
gi|3687320 cartilage-associated protein (CASP) [Mus musculus]
gi|26336999 unnamed protein product [Mus musculus]
gi|53830774 lactase [Mus musculus]
gi|51450 heat shock protein [Mus musculus]
gi|52789 unnamed protein product [Mus musculus]
gi|21489937 lanC-like protein 1 [Mus musculus]
gi|12859178 unnamed protein product [Mus musculus]
gi|19353667 Pcmtd2 protein, partial [Mus musculus]

Select Summary Report

Format As | Select Summary (protein hits) | Help

Significance threshold p< 0.05 Max. number of hits AUTO

Standard scoring ○ MudPIT scoring ◉ Ions score or expect cut-off 43 Show sub-sets 0

Show pop-ups ◉ Suppress pop-ups ○ Require bold red ☐

Re-Search ◉ All queries ○ Unassigned ○ Below homology threshold ○ Below identity threshold

1. gi|4159806 **Mass:** 65183 **Score:** 178 **Matches:** 5(5) **Sequences:** 2(2) **emPAI:** 0.10
type II keratin subunit protein [Mus musculus]

Query	Observed	Mr(expt)	Mr(calc)	Delta	Miss	Score	Expect	Rank	Unique	Peptide
14220	692.5523	1383.0901	1383.7034	-0.6133	1	49	0.022	1		K.SLNDKFASFIDK.V
15069	738.1921	1474.3697	1474.8144	-0.4446	1	81	1.3e-005	1	U	R.FLEQQNKVLQTK.W 15077 15082 15083

2. gi|46485130 **Mass:** 85186 **Score:** 171 **Matches:** 6(6) **Sequences:** 2(2) **emPAI:** 0.08
TPA_exp: keratin Kb40 [Mus musculus]

Query	Observed	Mr(expt)	Mr(calc)	Delta	Miss	Score	Expect	Rank	Unique	Peptide
15082	738.6157	1475.2169	1475.7984	-0.5815	1	85	2.5e-006	1	U	R.FLEQQNKVLETK.W 15077 15083 15090 15094
15719	820.4082	1638.8018	1637.8525	0.9493	1	55	0.0075	1	U	R.SLNNQFASFIDKVR.F

Proteins matching the same set of peptides:
gi|111185567 **Mass:** 54730 **Score:** 171 **Matches:** 6(6) **Sequences:** 2(2)
Krt78 protein [Mus musculus]
gi|111185722 **Mass:** 56744 **Score:** 171 **Matches:** 6(6) **Sequences:** 2(2)
Krt78 protein [Mus musculus]
gi|119850791 **Mass:** 54739 **Score:** 171 **Matches:** 6(6) **Sequences:** 2(2)
Krt78 protein [Mus musculus]
gi|145580629 **Mass:** 112194 **Score:** 171 **Matches:** 6(6) **Sequences:** 2(2)
keratin Kb40 [Mus musculus]

---

3. gi|12859782 **Mass:** 65586 **Score:** 152 **Matches:** 5(5) **Sequences:** 2(2) **emPAI:** 0.10
unnamed protein product [Mus musculus]

Query	Observed	Mr(expt)	Mr(calc)	Delta	Miss	Score	Expect	Rank	Unique	Peptide
14220	692.5523	1383.0901	1383.7034	-0.6133	1	49	0.022	1		K.SLNDKFASFIDK.V
15082	738.6157	1475.2169	1474.7780	0.4389	0	81	7.5e-006	3	U	R.FLEQQNQVLQTK.W 15069 15077 15083

Proteins matching the same set of peptides:
gi|126116585 **Mass:** 65565 **Score:** 152 **Matches:** 5(5) **Sequences:** 2(2)
keratin, type II cytoskeletal 1 [Mus musculus]

---

4. gi|47523977 **Mass:** 56715 **Score:** 145 **Matches:** 5(5) **Sequences:** 1(1) **emPAI:** 0.06
keratin, type II cytoskeletal 72 [Mus musculus]

Query	Observed	Mr(expt)	Mr(calc)	Delta	Miss	Score	Expect	Rank	Unique	Peptide
15082	738.6157	1475.2169	1475.7620	-0.5451	0	80	7.9e-006	4	U	R.FLEQQNQVLETK.W 15077 15083 15090 15094

---

5. gi|387397 **Mass:** 57807 **Score:** 122 **Matches:** 3(3) **Sequences:** 2(2) **emPAI:** 0.12
epidermal keratin subunit I, partial [Mus musculus]

Query	Observed	Mr(expt)	Mr(calc)	Delta	Miss	Score	Expect	Rank	Unique	Peptide
14203	**691.6326**	**1381.2506**	**1380.6408**	**0.6097**	**0**	**81**	**1.7e-005**	**1**	**U**	**R.ALEESNYELEGK.I**
14287	**696.0388**	**1390.0630**	**1389.6736**	**0.3894**	**0**	**72**	**0.00013**	**1**	**U**	**K.QSLEASLAETEGR.Y** 14292

Proteins matching the same set of peptides:
gi|12852157 **Mass:** 58587 **Score:** 122 **Matches:** 3(3) **Sequences:** 2(2)
unnamed protein product [Mus musculus]
gi|26349141 **Mass:** 52653 **Score:** 122 **Matches:** 3(3) **Sequences:** 2(2)
unnamed protein product [Mus musculus]
gi|26349459 **Mass:** 49471 **Score:** 122 **Matches:** 3(3) **Sequences:** 2(2)
unnamed protein product [Mus musculus]
gi|112983636 **Mass:** 57007 **Score:** 122 **Matches:** 3(3) **Sequences:** 2(2)
keratin, type I cytoskeletal 10 [Mus musculus]
gi|116242600 **Mass:** 57735 **Score:** 122 **Matches:** 3(3) **Sequences:** 2(2)
RecName: Full=Keratin, type I cytoskeletal 10; AltName: Full=56 kDa cytokeratin; AltName: Full=Cytokeratin-10; Short=CK-10; AltName: Full=Keratin, type I cytoskeletal 59 kDa; AltName: Full=Keratin-1[illegible]

---

6. gi|2498741 **Mass:** 60964 **Score:** 74 **Matches:** 1(1) **Sequences:** 1(1) **emPAI:** 0.05
RecName: Full=Prolyl 4-hydroxylase subunit alpha-2; Short=4-PH alpha-2; AltName: Full=Procollagen-proline,2-oxoglutarate-4-dioxygenase subunit alpha-2; Flags: Precursor

Query	Observed	Mr(expt)	Mr(calc)	Delta	Miss	Score	Expect	Rank	Unique	Peptide
10969	**533.6311**	**1065.2477**	**1065.5819**	**-0.3342**	**0**	**74**	**3.2e-005**	**1**	**U**	**K.TGVLTVASYR.V**

Proteins matching the same set of peptides:
gi|74216495 **Mass:** 21055 **Score:** 74 **Matches:** 1(1) **Sequences:** 1(1)
unnamed protein product [Mus musculus]
gi|148701597 **Mass:** 57417 **Score:** 74 **Matches:** 1(1) **Sequences:** 1(1)
procollagen-proline, 2-oxoglutarate 4-dioxygenase (proline 4-hydroxylase), alpha II polypeptide, isoform CRA_b [Mus musculus]
gi|148701598 **Mass:** 21273 **Score:** 74 **Matches:** 1(1) **Sequences:** 1(1)
procollagen-proline, 2-oxoglutarate 4-dioxygenase (proline 4-hydroxylase), alpha II polypeptide, isoform CRA_c [Mus musculus]
gi|148701599 **Mass:** 61292 **Score:** 74 **Matches:** 1(1) **Sequences:** 1(1)
procollagen-proline, 2-oxoglutarate 4-dioxygenase (proline 4-hydroxylase), alpha II polypeptide, isoform CRA_d [Mus musculus]

gi|148701600 **Mass:** 66870 **Score:** 74 **Matches:** 1(1) **Sequences:** 1(1)
procollagen-proline, 2-oxoglutarate 4-dioxygenase (proline 4-hydroxylase), alpha II polypeptide, isoform CRA_e [Mus musculus]
gi|209862961 **Mass:** 60774 **Score:** 74 **Matches:** 1(1) **Sequences:** 1(1)
prolyl 4-hydroxylase subunit alpha-2 isoform 1 precursor [Mus musculus]
gi|226874876 **Mass:** 60977 **Score:** 74 **Matches:** 1(1) **Sequences:** 1(1)
prolyl 4-hydroxylase subunit alpha-2 isoform 2 precursor [Mus musculus]

---

7. gi|16303309 **Mass:** 61743 **Score:** 73 **Matches:** 2(2) **Sequences:** 2(2) **emPAI:** 0.11
type II keratin 5 [Mus musculus]

Query	Observed	Mr(expt)	Mr(calc)	Delta	Miss	Score	Expect	Rank	Unique	Peptide
13007	621.5424	1241.0702	1241.5564	-0.4862	0	53	0.01	1	U	R.TEAESWYQTK.Y
14648	713.6679	1425.3211	1424.6680	0.6532	0	57	0.0037	1	U	R.VDALMDEINFMK.M

Proteins matching the same set of peptides:
gi|20911031 **Mass:** 61729 **Score:** 73 **Matches:** 2(2) **Sequences:** 2(2)
keratin, type II cytoskeletal 5 [Mus musculus]

---

8. gi|1389682 **Mass:** 68068 **Score:** 73 **Matches:** 1(1) **Sequences:** 1(1) **emPAI:** 0.05
plakoglobin, partial [Mus musculus]

Query	Observed	Mr(expt)	Mr(calc)	Delta	Miss	Score	Expect	Rank	Unique	Peptide
15139	742.2332	1482.4517	1482.7467	-0.2949	0	73	8.8e-005	1	U	R.NEGTATYAAAVLFR.I

Proteins matching the same set of peptides:
gi|28395018 **Mass:** 81749 **Score:** 73 **Matches:** 1(1) **Sequences:** 1(1)
junction plakoglobin [Mus musculus]

---

9. gi|16716569 **Mass:** 26118 **Score:** 65 **Matches:** 2(2) **Sequences:** 1(1) **emPAI:** 0.13
protease, serine, 1 precursor [Mus musculus]

Query	Observed	Mr(expt)	Mr(calc)	Delta	Miss	Score	Expect	Rank	Unique	Peptide
15061	738.0139	2211.0197	2210.0967	0.9230	0	59	0.0026	1	U	R.LGEHNINVLEGNEQFIDAAK.I 15060

---

10. gi|26337253 **Mass:** 53417 **Score:** 63 **Matches:** 1(1) **Sequences:** 1(1) **emPAI:** 0.06
unnamed protein product [Mus musculus]

Query	Observed	Mr(expt)	Mr(calc)	Delta	Miss	Score	Expect	Rank	Unique	Peptide
14805	722.0333	1442.0520	1441.7235	0.3285	0	63	0.0012	1	U	K.GPAGVELNSMQPVK.E

Proteins matching the same set of peptides:
gi|71060037 **Mass:** 53416 **Score:** 63 **Matches:** 1(1) **Sequences:** 1(1)
Slc2a3 [Mus musculus]
gi|261862282 **Mass:** 53444 **Score:** 63 **Matches:** 1(1) **Sequences:** 1(1)
solute carrier family 2, facilitated glucose transporter member 3 [Mus musculus]

---

11. gi|12846468 **Mass:** 40872 **Score:** 58 **Matches:** 1(1) **Sequences:** 1(1) **emPAI:** 0.08
unnamed protein product [Mus musculus]

Query	Observed	Mr(expt)	Mr(calc)	Delta	Miss	Score	Expect	Rank	Unique	Peptide
14387	700.5706	1399.1266	1398.7983	0.3283	0	58	0.0028	1	U	K.LLAAFPNLANLSR.T

Proteins matching the same set of peptides:
gi|26341012 **Mass:** 38517 **Score:** 58 **Matches:** 1(1) **Sequences:** 1(1)
unnamed protein product [Mus musculus]
gi|26343781 **Mass:** 33610 **Score:** 58 **Matches:** 1(1) **Sequences:** 1(1)
unnamed protein product [Mus musculus]
gi|26351471 **Mass:** 49011 **Score:** 58 **Matches:** 1(1) **Sequences:** 1(1)
unnamed protein product [Mus musculus]
gi|50510431 **Mass:** 45578 **Score:** 58 **Matches:** 1(1) **Sequences:** 1(1)
mKIAA0265 protein [Mus musculus]
gi|58037463 **Mass:** 48981 **Score:** 58 **Matches:** 1(1) **Sequences:** 1(1)

kelch domain-containing protein 10 [Mus musculus]
gi|62945268 **Mass:** 45351 **Score:** 58 **Matches:** 1(1) **Sequences:** 1(1)
kelch domain-containing protein 10 [Rattus norvegicus]
gi|148681799 **Mass:** 33511 **Score:** 58 **Matches:** 1(1) **Sequences:** 1(1)
RIKEN cDNA 2410127E18, isoform CRA_a [Mus musculus]
gi|148681802 **Mass:** 32113 **Score:** 58 **Matches:** 1(1) **Sequences:** 1(1)
RIKEN cDNA 2410127E18, isoform CRA_c [Mus musculus]

---

12. gi|6755893 **Mass:** 26257 **Score:** 57 **Matches:** 1(1) **Sequences:** 1(1) **emPAI:** 0.13
trypsin 4 precursor [Mus musculus]

Query	Observed	Mr(expt)	Mr(calc)	Delta	Miss	Score	Expect	Rank	Unique	Peptide
15071	738.3822	2212.1248	2211.0920	1.0328	0	57	0.0052	1	U	R.LGEHNINVLEGNEQFVNSAK.I

Proteins matching the same set of peptides:
gi|51010909 **Mass:** 26260 **Score:** 57 **Matches:** 1(1) **Sequences:** 1(1)
trypsin 5 precursor [Mus musculus]
gi|74203392 **Mass:** 27146 **Score:** 57 **Matches:** 1(1) **Sequences:** 1(1)
unnamed protein product [Mus musculus]

---

13. gi|3687320 **Mass:** 46137 **Score:** 57 **Matches:** 2(2) **Sequences:** 2(2) **emPAI:** 0.15
cartilage-associated protein (CASP) [Mus musculus]

Query	Observed	Mr(expt)	Mr(calc)	Delta	Miss	Score	Expect	Rank	Unique	Peptide
13636	662.2533	1322.4920	1322.6176	-0.1256	0	47	0.045	1	U	R.DELMPLESAYR.H
15767	840.3280	1678.6414	1678.8488	-0.2073	0	47	0.043	1	U	R.TSISDMELALPDFLK.A

Proteins matching the same set of peptides:
gi|12857227 **Mass:** 46121 **Score:** 57 **Matches:** 2(2) **Sequences:** 2(2)
unnamed protein product [Mus musculus]
gi|148677365 **Mass:** 46110 **Score:** 57 **Matches:** 2(2) **Sequences:** 2(2)
mCG7119 [Mus musculus]
gi|225543173 **Mass:** 46140 **Score:** 57 **Matches:** 2(2) **Sequences:** 2(2)
cartilage-associated protein precursor [Mus musculus]

---

14. gi|26336999 **Mass:** 60848 **Score:** 55 **Matches:** 1(1) **Sequences:** 1(1) **emPAI:** 0.05
unnamed protein product [Mus musculus]

Query	Observed	Mr(expt)	Mr(calc)	Delta	Miss	Score	Expect	Rank	Unique	Peptide
15096	739.4654	2215.3743	2215.1274	0.2470	1	55	0.006	1	U	R.LTSTATKDPEGFVGHPVNAFK.L

Proteins matching the same set of peptides:
gi|33859596 **Mass:** 60872 **Score:** 55 **Matches:** 1(1) **Sequences:** 1(1)
prolyl 4-hydroxylase subunit alpha-1 precursor [Mus musculus]
gi|74148153 **Mass:** 51722 **Score:** 55 **Matches:** 1(1) **Sequences:** 1(1)
unnamed protein product [Mus musculus]
gi|74224984 **Mass:** 60886 **Score:** 55 **Matches:** 1(1) **Sequences:** 1(1)
unnamed protein product [Mus musculus]
gi|74225936 **Mass:** 63769 **Score:** 55 **Matches:** 1(1) **Sequences:** 1(1)
unnamed protein product [Mus musculus]

---

15. gi|53830774 **Mass:** 34092 **Score:** 49 **Matches:** 1(1) **Sequences:** 1(1) **emPAI:** 0.10
lactase [Mus musculus]

Query	Observed	Mr(expt)	Mr(calc)	Delta	Miss	Score	Expect	Rank	Unique	Peptide
11285	547.7469	1093.4792	1094.5132	-1.0339	0	49	0.014	1	U	R.LPEFTESEK.K

Proteins matching the same set of peptides:
gi|74192292 **Mass:** 139170 **Score:** 49 **Matches:** 1(1) **Sequences:** 1(1)
unnamed protein product [Mus musculus]
gi|124487297 **Mass:** 217665 **Score:** 49 **Matches:** 1(1) **Sequences:** 1(1)

lactase-phlorizin hydrolase preproprotein [Mus musculus]
gi|148707805 **Mass:** 217693 **Score:** 49 **Matches:** 1(1) **Sequences:** 1(1)
mCG128560 [Mus musculus]

---

16. gi|51450 **Mass:** 46560 **Score:** 48 **Matches:** 1(1) **Sequences:** 1(1) **emPAI:** 0.07
heat shock protein [Mus musculus]

Query	Observed	Mr(expt)	Mr(calc)	Delta	Miss	Score	Expect	Rank	Unique	Peptide
12831	613.0960	1224.1775	1223.6510	0.5265	0	48	0.012	1	U	K.GVVEVTHDLQK.H

Proteins matching the same set of peptides:
gi|200966 **Mass:** 45623 **Score:** 48 **Matches:** 1(1) **Sequences:** 1(1)
put. serine protease inhibitor [Mus musculus]
gi|26345418 **Mass:** 46481 **Score:** 48 **Matches:** 1(1) **Sequences:** 1(1)
unnamed protein product [Mus musculus]
gi|26348007 **Mass:** 46490 **Score:** 48 **Matches:** 1(1) **Sequences:** 1(1)
unnamed protein product [Mus musculus]
gi|74139266 **Mass:** 46490 **Score:** 48 **Matches:** 1(1) **Sequences:** 1(1)
unnamed protein product [Mus musculus]
gi|74191337 **Mass:** 46505 **Score:** 48 **Matches:** 1(1) **Sequences:** 1(1)
unnamed protein product [Mus musculus]
gi|74198254 **Mass:** 46500 **Score:** 48 **Matches:** 1(1) **Sequences:** 1(1)
unnamed protein product [Mus musculus]
gi|148684430 **Mass:** 44954 **Score:** 48 **Matches:** 1(1) **Sequences:** 1(1)
serine (or cysteine) peptidase inhibitor, clade H, member 1, isoform CRA_a [Mus musculus]
gi|161353502 **Mass:** 46504 **Score:** 48 **Matches:** 1(1) **Sequences:** 1(1)
serpin H1 precursor [Mus musculus]

---

17. gi|52789 **Mass:** 54415 **Score:** 45 **Matches:** 1(1) **Sequences:** 1(1) **emPAI:** 0.06
unnamed protein product [Mus musculus]

Query	Observed	Mr(expt)	Mr(calc)	Delta	Miss	Score	Expect	Rank	Unique	Peptide
14209	692.0989	1382.1832	1382.7194	-0.5362	1	45	0.058	1	U	K.SLNNKFASFIDK.V

Proteins matching the same set of peptides:
gi|309215 **Mass:** 53210 **Score:** 45 **Matches:** 1(1) **Sequences:** 1(1)
EndoA' cytokeratin (5' end put.); putative [Mus musculus]
gi|511654 **Mass:** 54220 **Score:** 45 **Matches:** 1(1) **Sequences:** 1(1)
keratin type II [Mus musculus]
gi|74177777 **Mass:** 54546 **Score:** 45 **Matches:** 1(1) **Sequences:** 1(1)
unnamed protein product [Mus musculus]
gi|74219975 **Mass:** 54459 **Score:** 45 **Matches:** 1(1) **Sequences:** 1(1)
unnamed protein product [Mus musculus]
gi|76779293 **Mass:** 54514 **Score:** 45 **Matches:** 1(1) **Sequences:** 1(1)
Keratin 8 [Mus musculus]
gi|114145561 **Mass:** 54531 **Score:** 45 **Matches:** 1(1) **Sequences:** 1(1)
keratin, type II cytoskeletal 8 [Mus musculus]

---

18. gi|21489937 **Mass:** 45312 **Score:** 45 **Matches:** 1(1) **Sequences:** 1(1) **emPAI:** 0.07
lanC-like protein 1 [Mus musculus]

Query	Observed	Mr(expt)	Mr(calc)	Delta	Miss	Score	Expect	Rank	Unique	Peptide
10769	524.0907	1046.1668	1045.5226	0.6442	0	45	0.029	1	U	R.ELLQQMER.G

Proteins matching the same set of peptides:
gi|148667828 **Mass:** 45346 **Score:** 45 **Matches:** 1(1) **Sequences:** 1(1)
LanC (bacterial lantibiotic synthetase component C)-like 1, isoform CRA_a [Mus musculus]
gi|148667829 **Mass:** 47990 **Score:** 45 **Matches:** 1(1) **Sequences:** 1(1)
LanC (bacterial lantibiotic synthetase component C)-like 1, isoform CRA_b [Mus musculus]

---

19. gi|12859178 **Mass:** 26301 **Score:** 45 **Matches:** 1(0) **Sequences:** 1(0) **emPAI:** 0.13

unnamed protein product [Mus musculus]

Query	Observed	Mr(expt)	Mr(calc)	Delta	Miss	Score	Expect	Rank	Unique	Peptide
13273	639.2183	1914.6330	1913.8795	0.7534	1	45	0.072	1	U	K.DAEEWFFTKTEELNR.E

Proteins matching the same set of peptides:

gi\|38503465 **Mass:** 50146 **Score:** 45 **Matches:** 1(0) **Sequences:** 1(0)
keratin 17n [Mus musculus]

gi\|85726518 **Mass:** 49857 **Score:** 45 **Matches:** 1(0) **Sequences:** 1(0)
Krt42 protein [Mus musculus]

gi\|154090941 **Mass:** 50102 **Score:** 45 **Matches:** 1(0) **Sequences:** 1(0)
keratin, type I cytoskeletal 42 [Mus musculus]

gi\|156139032 **Mass:** 50162 **Score:** 45 **Matches:** 1(0) **Sequences:** 1(0)
Keratin 42 [Mus musculus]

---

20. gi\|19353667 **Mass:** 19691 **Score:** 43 **Matches:** 2(1) **Sequences:** 2(1) **emPAI:** 0.17
Pcmtd2 protein, partial [Mus musculus]

Query	Observed	Mr(expt)	Mr(calc)	Delta	Miss	Score	Expect	Rank	Unique	Peptide
10138	489.0460	976.0774	975.4662	0.6112	0	43	0.045	1	U	R.TGPSAWETK.K
11516	556.1887	1110.3629	1110.5631	-0.2002	0	43	0.1	1	U	R.METIVFLDK.E

Proteins matching the same set of peptides:

gi\|23956398 **Mass:** 40730 **Score:** 43 **Matches:** 2(1) **Sequences:** 2(1)
protein-L-isoaspartate O-methyltransferase domain-containing protein 2 [Mus musculus]

gi\|26348291 **Mass:** 21087 **Score:** 43 **Matches:** 2(1) **Sequences:** 2(1)
unnamed protein product [Mus musculus]

Mascot: http://www.matrixscience.com/

Mascot Search Results

D17

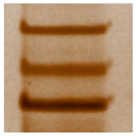

A

```
User                   :
Email                  :
Search title           :
MS data file           : ZIEN-29Nov2017_flyA.mgf
Database               : NCBInr 20120419 (17893860 sequences; 6141683785 residues)
Taxonomy               : Drosophila (fruit flies) (222527 sequences)
Timestamp              : 30 Nov 2017 at 18:31:47 GMT
Enzyme                 : Trypsin
Variable modifications : Carbamidomethyl (C),Oxidation (M)
Mass values            : Monoisotopic
Protein Mass           : Unrestricted
Peptide Mass Tolerance : ± 1.25 Da
Fragment Mass Tolerance: ± 1.001 Da
Max Missed Cleavages   : 1
Instrument type        : ESI-TRAP
Number of queries      : 16095
Protein hits           : gi|17737967  heat shock protein cognate 4, isoform A [Drosophila melanogaster]
                         gi|157658    heat shock protein cognate 72 [Drosophila melanogaster]
                         gi|157667    heat shock protein cognate 71 [Drosophila melanogaster]
                         gi|157678    heat shock cognate 70 protein (partial) (at locus 88E), partial [Drosophila melanogaster]
                         gi|6118254   DEAD-box protein abstrakt [Drosophila melanogaster]
                         gi|195449894 GK22765 [Drosophila willistoni]
                         gi|24651407  prolyl-4-hydroxylase-alpha EFB [Drosophila melanogaster]
                         gi|195341536 GM12882 [Drosophila sechellia]
                         gi|24644396  CG2082, isoform C [Drosophila melanogaster]
                         gi|157891    myosin heavy chain [Drosophila melanogaster]
                         gi|195161163 GL25329 [Drosophila persimilis]
                         gi|194864176 GG23181 [Drosophila erecta]
                         gi|194769542 GF19055 [Drosophila ananassae]
                         gi|194766886 GF22554 [Drosophila ananassae]
```

Select Summary Report

Format As | Select Summary (protein hits) ▼ | Help

Significance threshold p< 0.05 | Max. number of hits AUTO

Standard scoring ○ MudPIT scoring ◉ | Ions score or expect cut-off 44 | Show sub-sets 0

Show pop-ups ◉ Suppress pop-ups ○ | Require bold red ☐

Re-Search ◉ All queries ○ Unassigned ○ Below homology threshold ○ Below identity threshold

1. gi|17737967 **Mass:** 71087 **Score:** 168 **Matches:** 7(7) **Sequences:** 6(6) **emPAI:** 0.31
heat shock protein cognate 4, isoform A [Drosophila melanogaster]

Query	Observed	Mr(expt)	Mr(calc)	Delta	Miss	Score	Expect	Rank	Unique	Peptide
12119	587.4327	1172.8509	1172.5826	0.2684	0	51	0.011	1	U	K.WLDANQLADK.E
12465	600.5588	1199.1031	1198.6670	0.4362	0	57	0.0044	1	U	K.DAGTIAGLNVLR.I 12466
12751	613.9276	1225.8405	1225.5285	0.3120	0	49	0.032	1		K.FDDAAVQSDMK.H
13970	678.0540	1354.0934	1353.6235	0.4699	1	53	0.011	1		R.KFDDAAVQSDMK.H
15110	727.5170	1453.0195	1451.8096	1.2099	0	76	3e-005	1	U	K.SVIHDIVLVGGSTR.I
15964	839.5435	1677.0724	1676.8192	0.2532	0	92	1.2e-006	1	U	K.NQVAMNPTQTIFDAK.R

Proteins matching the same set of peptides:

gi|19527633 **Mass:** 71060 **Score:** 168 **Matches:** 7(7) **Sequences:** 6(6)
RH04426p [Drosophila melanogaster]

gi|194900946 **Mass:** 71001 **Score:** 168 **Matches:** 7(7) **Sequences:** 6(6)
GG16900 [Drosophila erecta]

gi|195328813 **Mass:** 70971 **Score:** 168 **Matches:** 7(7) **Sequences:** 6(6)
GM24208 [Drosophila sechellia]

gi|195570732 **Mass:** 70916 **Score:** 168 **Matches:** 7(7) **Sequences:** 6(6)
Hsc70-4 [Drosophila simulans]

---

2. gi|157658 **Mass:** 72190 **Score:** 108 **Matches:** 5(5) **Sequences:** 5(5) **emPAI:** 0.25
heat shock protein cognate 72 [Drosophila melanogaster]

Query	Observed	Mr(expt)	Mr(calc)	Delta	Miss	Score	Expect	Rank	Unique	Peptide
9446	478.0703	954.1261	953.5043	0.6218	0	62	0.00066	1	U	R.ALSGSHQVR.I
11066	552.6559	1103.2972	1103.5710	-0.2738	0	56	0.0035	1	U	K.LESAIDESIK.W
13643	659.0761	1316.1375	1315.6295	0.5080	0	55	0.006	1	U	R.NELESYAYSLK.N
15556	754.5173	1507.0201	1505.7800	1.2402	0	51	0.015	1	U	K.VFAPEEISAMVLGK.M
15667	764.1249	1526.2352	1525.7413	0.4939	0	48	0.028	1	U	R.ITPSYVAFTADGER.L

Proteins matching the same set of peptides:

gi|24641402 **Mass:** 72216 **Score:** 108 **Matches:** 5(5) **Sequences:** 5(5)
heat shock protein cognate 3, isoform A [Drosophila melanogaster]

gi|28557577 **Mass:** 72069 **Score:** 108 **Matches:** 5(5) **Sequences:** 5(5)
RH21402p [Drosophila melanogaster]

gi|125981509 **Mass:** 72261 **Score:** 108 **Matches:** 5(5) **Sequences:** 5(5)
GA17988 [Drosophila pseudoobscura pseudoobscura]

gi|194762782 **Mass:** 72278 **Score:** 108 **Matches:** 5(5) **Sequences:** 5(5)
GF20248 [Drosophila ananassae]
gi|195165202 **Mass:** 72272 **Score:** 108 **Matches:** 5(5) **Sequences:** 5(5)
GL20354 [Drosophila persimilis]
gi|195457222 **Mass:** 72381 **Score:** 108 **Matches:** 5(5) **Sequences:** 5(5)
GK18328 [Drosophila willistoni]
gi|195480743 **Mass:** 72226 **Score:** 108 **Matches:** 5(5) **Sequences:** 5(5)
Hsc70-3 [Drosophila yakuba]

---

3. gi|157667 **Mass:** 74162 **Score:** 100 **Matches:** 6(5) **Sequences:** 5(4) **emPAI:** 0.24
heat shock protein cognate 71 [Drosophila melanogaster]

Query	Observed	Mr(expt)	Mr(calc)	Delta	Miss	Score	Expect	Rank	Unique	Peptide
9395	953.4493	952.4420	952.4865	-0.0445	0	51	0.013	1	U	K.DITNLSYK.V
9494	958.5143	957.5070	957.4879	0.0191	0	45	0.064	1	U	K.VIENAEGAR.T
11337	563.4215	1124.8284	1124.5349	0.2935	1	49	0.015	1	U	K.KAEEYATADK.Q
12759	614.1332	1226.2518	1225.6779	0.5739	0	66	0.00049	1	U	K.DAGQIAGLNVLR.V 12742
13805	668.6088	1335.2030	1334.6718	0.5312	0	45	0.035	1	U	K.ETAEAYLNTPVK.N

Proteins matching the same set of peptides:
gi|24653595 **Mass:** 74020 **Score:** 100 **Matches:** 6(5) **Sequences:** 5(4)
heat shock protein cognate 5 [Drosophila melanogaster]
gi|194883130 **Mass:** 74029 **Score:** 100 **Matches:** 6(5) **Sequences:** 5(4)
GG22433 [Drosophila erecta]
gi|195334294 **Mass:** 74060 **Score:** 100 **Matches:** 6(5) **Sequences:** 5(4)
GM20220 [Drosophila sechellia]
gi|195485915 **Mass:** 74021 **Score:** 100 **Matches:** 6(5) **Sequences:** 5(4)
GE12323 [Drosophila yakuba]
gi|195583328 **Mass:** 74074 **Score:** 100 **Matches:** 6(5) **Sequences:** 5(4)
GD25692 [Drosophila simulans]
gi|194756900 **Mass:** 73926 **Score:** 100 **Matches:** 6(5) **Sequences:** 5(4)
GF11360 [Drosophila ananassae]

---

4. gi|157678 **Mass:** 11411 **Score:** 97 **Matches:** 3(3) **Sequences:** 3(3) **emPAI:** 1.20
heat shock cognate 70 protein (partial) (at locus 88E), partial [Drosophila melanogaster]

Query	Observed	Mr(expt)	Mr(calc)	Delta	Miss	Score	Expect	Rank	Unique	Peptide
12751	613.9276	1225.8405	1225.5285	0.3120	0	49	0.032	1		K.FDDAAVQSDMK.H
13970	678.0540	1354.0934	1353.6235	0.4699	1	53	0.011	1		R.KFDDAAVQSDMK.H
15964	839.5435	1677.0724	1677.8032	-0.7308	0	79	2.5e-005	2	U	K.NEVAMNPTQTIFDAK.R

---

5. gi|6118254 **Mass:** 68888 **Score:** 60 **Matches:** 1(1) **Sequences:** 1(1) **emPAI:** 0.05
DEAD-box protein abstrakt [Drosophila melanogaster]

Query	Observed	Mr(expt)	Mr(calc)	Delta	Miss	Score	Expect	Rank	Unique	Peptide
14629	704.5223	1407.0300	1406.7769	0.2531	0	60	0.002	1	U	R.ILVEGETPSPPIR.S

Proteins matching the same set of peptides:
gi|17977678 **Mass:** 69444 **Score:** 60 **Matches:** 1(1) **Sequences:** 1(1)
abstrakt [Drosophila melanogaster]

---

6. gi|195449894 **Mass:** 21978 **Score:** 57 **Matches:** 1(1) **Sequences:** 1(1) **emPAI:** 0.15
GK22765 [Drosophila willistoni]

Query	Observed	Mr(expt)	Mr(calc)	Delta	Miss	Score	Expect	Rank	Unique	Peptide
9376	475.9925	949.9705	950.4127	-0.4422	0	57	0.0025	1	U	M.TTANACDQK.G

---

7. gi|24651407 **Mass:** 63075 **Score:** 53 **Matches:** 1(1) **Sequences:** 1(1) **emPAI:** 0.05
prolyl-4-hydroxylase-alpha EFB [Drosophila melanogaster]

Query	Observed	Mr(expt)	Mr(calc)	Delta	Miss	Score	Expect	Rank	Unique	Peptide
10856	545.6321	1089.2496	1089.5567	-0.3071	0	53	0.012	1	U	R.AFEGLNLGNR.I

Proteins matching the same set of peptides:
gi|125772807 **Mass:** 62641 **Score:** 53 **Matches:** 1(1) **Sequences:** 1(1)
GA15946 [Drosophila pseudoobscura pseudoobscura]
gi|194765194 **Mass:** 62567 **Score:** 53 **Matches:** 1(1) **Sequences:** 1(1)
GF22904 [Drosophila ananassae]
gi|194905436 **Mass:** 62737 **Score:** 53 **Matches:** 1(1) **Sequences:** 1(1)
GG11753 [Drosophila erecta]
gi|195110919 **Mass:** 55326 **Score:** 53 **Matches:** 1(1) **Sequences:** 1(1)
GI24860 [Drosophila mojavensis]
gi|195159323 **Mass:** 55783 **Score:** 53 **Matches:** 1(1) **Sequences:** 1(1)
GL13463 [Drosophila persimilis]
gi|195391754 **Mass:** 55603 **Score:** 53 **Matches:** 1(1) **Sequences:** 1(1)
GJ24502 [Drosophila virilis]
gi|195452726 **Mass:** 62836 **Score:** 53 **Matches:** 1(1) **Sequences:** 1(1)
GK14136 [Drosophila willistoni]
gi|195505190 **Mass:** 55776 **Score:** 53 **Matches:** 1(1) **Sequences:** 1(1)
GE10881 [Drosophila yakuba]
gi|195575089 **Mass:** 62991 **Score:** 53 **Matches:** 1(1) **Sequences:** 1(1)
GD21521 [Drosophila simulans]

8. gi|195341536 **Mass:** 63009 **Score:** 53 **Matches:** 1(1) **Sequences:** 1(1) **emPAI:** 0.05
GM12882 [Drosophila sechellia]

Query	Observed	Mr(expt)	Mr(calc)	Delta	Miss	Score	Expect	Rank	Unique	Peptide
10856	545.6321	1089.2496	1089.5567	-0.3071	0	53	0.012	1	U	R.AFEGINLGNR.I

9. gi|24644396 **Mass:** 39679 **Score:** 48 **Matches:** 1(1) **Sequences:** 1(1) **emPAI:** 0.08
CG2082, isoform C [Drosophila melanogaster]

Query	Observed	Mr(expt)	Mr(calc)	Delta	Miss	Score	Expect	Rank	Unique	Peptide
12547	**604.5787**	**1810.7142**	**1810.8558**	**-0.1416**	**1**	**48**	**0.036**	**1**	**U**	**R.GRAFSTASSGSLEGANGSR.R**

Proteins matching the same set of peptides:

gi|24644398 **Mass:** 39652 **Score:** 48 **Matches:** 1(1) **Sequences:** 1(1)
CG2082, isoform A [Drosophila melanogaster]

gi|62484286 **Mass:** 40038 **Score:** 48 **Matches:** 1(1) **Sequences:** 1(1)
CG2082, isoform B [Drosophila melanogaster]

gi|194745478 **Mass:** 40170 **Score:** 48 **Matches:** 1(1) **Sequences:** 1(1)
GF18647 [Drosophila ananassae]

gi|194898877 **Mass:** 39987 **Score:** 48 **Matches:** 1(1) **Sequences:** 1(1)
GG10854 [Drosophila erecta]

gi|195343799 **Mass:** 41940 **Score:** 48 **Matches:** 1(1) **Sequences:** 1(1)
GM10591 [Drosophila sechellia]

gi|262331602 **Mass:** 19636 **Score:** 48 **Matches:** 1(1) **Sequences:** 1(1)
AT13994p [Drosophila melanogaster]

gi|281360117 **Mass:** 40012 **Score:** 48 **Matches:** 1(1) **Sequences:** 1(1)
CG2082, isoform G [Drosophila melanogaster]

gi|281360119 **Mass:** 39979 **Score:** 48 **Matches:** 1(1) **Sequences:** 1(1)
CG2082, isoform H [Drosophila melanogaster]

gi|327180770 **Mass:** 41129 **Score:** 48 **Matches:** 1(1) **Sequences:** 1(1)
RH63159p [Drosophila melanogaster]

gi|383292515 **Mass:** 36950 **Score:** 48 **Matches:** 1(1) **Sequences:** 1(1)
CG2082, isoform O [Drosophila melanogaster]

10. gi|157891 **Score:** 48 **Matches:** 1(1) **Sequences:** 1(1) **emPAI:** 0.01
myosin heavy chain [Drosophila melanogaster]

Query	Observed	Mr(expt)	Mr(calc)	Delta	Miss	Score	Expect	Rank	Unique	Peptide
9376	475.9925	949.9705	950.4127	-0.4422	0	48	0.019	2	U	K.MQGETNQK.T

Proteins matching the same set of peptides:

gi|157892 **Score:** 48 **Matches:** 1(1) **Sequences:** 1(1)
gi|2546936 **Score:** 48 **Matches:** 1(1) **Sequences:** 1(1)
gi|2546937 **Score:** 48 **Matches:** 1(1) **Sequences:** 1(1)
gi|2546938 **Score:** 48 **Matches:** 1(1) **Sequences:** 1(1)
gi|2546939 **Score:** 48 **Matches:** 1(1) **Sequences:** 1(1)
gi|24584692 **Score:** 48 **Matches:** 1(1) **Sequences:** 1(1)
gi|24584694 **Score:** 48 **Matches:** 1(1) **Sequences:** 1(1)
gi|24584696 **Score:** 48 **Matches:** 1(1) **Sequences:** 1(1)
gi|24584700 **Score:** 48 **Matches:** 1(1) **Sequences:** 1(1)
gi|24584702 **Score:** 48 **Matches:** 1(1) **Sequences:** 1(1)
gi|24584704 **Score:** 48 **Matches:** 1(1) **Sequences:** 1(1)
gi|24584706 **Score:** 48 **Matches:** 1(1) **Sequences:** 1(1)
gi|24584710 **Score:** 48 **Matches:** 1(1) **Sequences:** 1(1)
gi|24584712 **Score:** 48 **Matches:** 1(1) **Sequences:** 1(1)
gi|24584714 **Score:** 48 **Matches:** 1(1) **Sequences:** 1(1)
gi|24584716 **Score:** 48 **Matches:** 1(1) **Sequences:** 1(1)
gi|28574239 **Score:** 48 **Matches:** 1(1) **Sequences:** 1(1)
gi|110825729 **Score:** 48 **Matches:** 1(1) **Sequences:** 1(1)
gi|194884434 **Score:** 48 **Matches:** 1(1) **Sequences:** 1(1)
gi|195344578 **Score:** 48 **Matches:** 1(1) **Sequences:** 1(1)
gi|195436762 **Score:** 48 **Matches:** 1(1) **Sequences:** 1(1)
gi|195483992 **Score:** 48 **Matches:** 1(1) **Sequences:** 1(1)
gi|198474044 **Score:** 48 **Matches:** 1(1) **Sequences:** 1(1)
gi|219990777 **Score:** 48 **Matches:** 1(1) **Sequences:** 1(1)
gi|281365095 **Score:** 48 **Matches:** 1(1) **Sequences:** 1(1)
gi|281365099 **Score:** 48 **Matches:** 1(1) **Sequences:** 1(1)
gi|383291520 **Score:** 48 **Matches:** 1(1) **Sequences:** 1(1)
gi|383291521 **Score:** 48 **Matches:** 1(1) **Sequences:** 1(1)
gi|383291522 **Score:** 48 **Matches:** 1(1) **Sequences:** 1(1)
gi|383291523 **Score:** 48 **Matches:** 1(1) **Sequences:** 1(1)
gi|383291524 **Score:** 48 **Matches:** 1(1) **Sequences:** 1(1)

---

11. gi|195161163 **Mass:** 58891 **Score:** 47 **Matches:** 1(1) **Sequences:** 1(1) **emPAI:** 0.06
GL25329 [Drosophila persimilis]

Query	Observed	Mr(expt)	Mr(calc)	Delta	Miss	Score	Expect	Rank	Unique	Peptide
12491	**601.5865**	**1201.1585**	**1200.5232**	**0.6353**	**0**	**47**	**0.05**	**1**	**U**	**R.QDNGGGGGVGGGGGR.F**

---

12. gi|194864176 **Score:** 46 **Matches:** 1(1) **Sequences:** 1(1) **emPAI:** 0.02
GG23181 [Drosophila erecta]

Query	Observed	Mr(expt)	Mr(calc)	Delta	Miss	Score	Expect	Rank	Unique	Peptide
12466	600.5885	1199.1624	1198.6670	0.4955	0	46	0.058	2	U	R.ETNNVIAIGLR.D

13. gi|194769542 **Mass:** 194372 **Score:** 46 **Matches:** 1(1) **Sequences:** 1(1) **emPAI:** 0.02
GF19055 [Drosophila ananassae]

Query	Observed	Mr(expt)	Mr(calc)	Delta	Miss	Score	Expect	Rank	Unique	Peptide
9300	**472.3273**	**942.6400**	**942.5498**	**0.0902**	**0**	**46**	**0.046**	**1**	**U**	**K.VIEISVQR.G**

Proteins matching the same set of peptides:

gi|194892726 **Mass:** 191336 **Score:** 46 **Matches:** 1(1) **Sequences:** 1(1)
GG19193 [Drosophila erecta]

gi|195132717 **Mass:** 70104 **Score:** 46 **Matches:** 1(1) **Sequences:** 1(1)
GI21732 [Drosophila mojavensis]

gi|195392652 **Mass:** 186045 **Score:** 46 **Matches:** 1(1) **Sequences:** 1(1)
GJ19114 [Drosophila virilis]

gi|195432565 **Mass:** 215970 **Score:** 46 **Matches:** 1(1) **Sequences:** 1(1)
GK19785 [Drosophila willistoni]

gi|195481506 **Mass:** 198954 **Score:** 46 **Matches:** 1(1) **Sequences:** 1(1)
GE17755 [Drosophila yakuba]

gi|198469723 **Mass:** 196607 **Score:** 46 **Matches:** 1(1) **Sequences:** 1(1)
GA23340 [Drosophila pseudoobscura pseudoobscura]

14. gi|194766886 **Score:** 45 **Matches:** 1(1) **Sequences:** 1(1) **emPAI:** 0.01
GF22554 [Drosophila ananassae]

Query	Observed	Mr(expt)	Mr(calc)	Delta	Miss	Score	Expect	Rank	Unique	Peptide
9376	475.9925	949.9705	950.4742	-0.5037	1	45	0.034	3	U	K.MKSAELEK.E

Mascot: http://www.matrixscience.com/

Mascot Search Results

D17

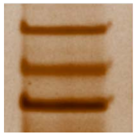

B

```
User                   :
Email                  :
Search title           :
MS data file           : ZIEN-29Nov2017_flyB.mgf
Database               : NCBInr 20120419 (17893860 sequences; 6141683785 residues)
Taxonomy               : Drosophila (fruit flies) (222527 sequences)
Timestamp              : 30 Nov 2017 at 18:51:10 GMT
Enzyme                 : Trypsin
Variable modifications : Carbamidomethyl (C),Oxidation (M)
Mass values            : Monoisotopic
Protein Mass           : Unrestricted
Peptide Mass Tolerance : ± 1.25 Da
Fragment Mass Tolerance: ± 1.001 Da
Max Missed Cleavages   : 1
Instrument type        : Default
Number of queries      : 15798
Protein hits           : gi|17647799  protein disulfide isomerase, isoform A [Drosophila melanogaster]
                         gi|24664683  CG16979 [Drosophila melanogaster]
                         gi|195451942 GK13309 [Drosophila willistoni]
                         gi|24644396  CG2082, isoform C [Drosophila melanogaster]
                         gi|198476294 GA25398 [Drosophila pseudoobscura pseudoobscura]
                         gi|24640588  CG1440, isoform A [Drosophila melanogaster]
                         gi|24651407  prolyl-4-hydroxylase-alpha EFB [Drosophila melanogaster]
                         gi|195161605 GL26392 [Drosophila persimilis]
                         gi|195450712 GK13603 [Drosophila willistoni]
```

Select Summary Report

Format As | Select Summary (protein hits) ▼ | Help

Significance threshold p< 0.05 | Max. number of hits AUTO

Standard scoring ○ MudPIT scoring ◉ | Ions score or expect cut-off 44 | Show sub-sets 0

Show pop-ups ◉ Suppress pop-ups ○ | Require bold red ☐

Re-Search | ◉ All queries ○ Unassigned ○ Below homology threshold ○ Below identity threshold

1. gi|17647799 **Mass:** 55746 **Score:** 120 **Matches:** 3(3) **Sequences:** 3(3) **emPAI:** 0.19
protein disulfide isomerase, isoform A [Drosophila melanogaster]

Query	Observed	Mr(expt)	Mr(calc)	Delta	Miss	Score	Expect	Rank	Unique	Peptide

12211	608.3029	1214.5912	1214.6659	-0.0747	0	55	0.0097	1	U	R.QAADIIAWVTK.K
13260	669.5514	1337.0882	1336.6180	0.4702	0	86	2.5e-006	1	U	K.MDSTANELESIK.I
15162	754.9669	1507.9192	1506.7202	1.1990	0	62	0.00091	1	U	K.SVFEGELNEENLK.K

Proteins matching the same set of peptides:

gi|195327719 **Mass:** 55772 **Score:** 120 **Matches:** 3(3) **Sequences:** 3(3)
GM24501 [Drosophila sechellia]

---

2. gi|24664683 **Mass:** 68207 **Score:** 66 **Matches:** 1(1) **Sequences:** 1(1) **emPAI:** 0.05
CG16979 [Drosophila melanogaster]

Query	Observed	Mr(expt)	Mr(calc)	Delta	Miss	Score	Expect	Rank	Unique	Peptide
15088	751.1436	1500.2727	1500.7937	-0.5210	0	66	0.00048	1	U	R.VADGNLVFNVPQTK.I

Proteins matching the same set of peptides:

gi|194872892 **Mass:** 68503 **Score:** 66 **Matches:** 1(1) **Sequences:** 1(1)
GG15911 [Drosophila erecta]

gi|195327809 **Mass:** 68273 **Score:** 66 **Matches:** 1(1) **Sequences:** 1(1)
GM25541 [Drosophila sechellia]

gi|195477958 **Mass:** 68443 **Score:** 66 **Matches:** 1(1) **Sequences:** 1(1)
GE23132 [Drosophila yakuba]

gi|195590489 **Mass:** 68190 **Score:** 66 **Matches:** 1(1) **Sequences:** 1(1)
GD14556 [Drosophila simulans]

---

3. gi|195451942 **Mass:** 253843 **Score:** 53 **Matches:** 1(1) **Sequences:** 1(1) **emPAI:** 0.01
GK13309 [Drosophila willistoni]

Query	Observed	Mr(expt)	Mr(calc)	Delta	Miss	Score	Expect	Rank	Unique	Peptide
15598	801.8900	1601.7655	1600.7917	0.9737	0	53	0.012	1	U	R.NGGLGAAGIVASNSGSNR.V

---

4. gi|24644396 **Mass:** 39679 **Score:** 52 **Matches:** 1(1) **Sequences:** 1(1) **emPAI:** 0.08
CG2082, isoform C [Drosophila melanogaster]

Query	Observed	Mr(expt)	Mr(calc)	Delta	Miss	Score	Expect	Rank	Unique	Peptide
12135	604.6827	1811.0264	1810.8558	0.1706	1	52	0.016	1	U	R.GRAFSTASSGSLEGANGSR.R

Proteins matching the same set of peptides:

gi|24644398 **Mass:** 39652 **Score:** 52 **Matches:** 1(1) **Sequences:** 1(1)
CG2082, isoform A [Drosophila melanogaster]

gi|62484286 **Mass:** 40038 **Score:** 52 **Matches:** 1(1) **Sequences:** 1(1)

CG2082, isoform B [Drosophila melanogaster]

gi|194745478 **Mass:** 40170 **Score:** 52 **Matches:** 1(1) **Sequences:** 1(1)
GF18647 [Drosophila ananassae]

gi|194898877 **Mass:** 39987 **Score:** 52 **Matches:** 1(1) **Sequences:** 1(1)
GG10854 [Drosophila erecta]

gi|195343799 **Mass:** 41940 **Score:** 52 **Matches:** 1(1) **Sequences:** 1(1)
GM10591 [Drosophila sechellia]

gi|262331602 **Mass:** 19636 **Score:** 52 **Matches:** 1(1) **Sequences:** 1(1)
AT13994p [Drosophila melanogaster]

gi|281360117 **Mass:** 40012 **Score:** 52 **Matches:** 1(1) **Sequences:** 1(1)
CG2082, isoform G [Drosophila melanogaster]

gi|281360119 **Mass:** 39979 **Score:** 52 **Matches:** 1(1) **Sequences:** 1(1)
CG2082, isoform H [Drosophila melanogaster]

gi|327180770 **Mass:** 41129 **Score:** 52 **Matches:** 1(1) **Sequences:** 1(1)
RH63159p [Drosophila melanogaster]

gi|383292515 **Mass:** 36950 **Score:** 52 **Matches:** 1(1) **Sequences:** 1(1)
CG2082, isoform O [Drosophila melanogaster]

---

5. gi|198476294 **Mass:** 41611 **Score:** 49 **Matches:** 1(1) **Sequences:** 1(1) **emPAI:** 0.08
GA25398 [Drosophila pseudoobscura pseudoobscura]

Query	Observed	Mr(expt)	Mr(calc)	Delta	Miss	Score	Expect	Rank	Unique	Peptide
14689	729.6755	1457.3365	1456.7885	0.5480	1	49	0.025	1	U	R.ETQIKNNIEQIK.D

---

6. gi|24640588 **Mass:** 55121 **Score:** 49 **Matches:** 1(1) **Sequences:** 1(1) **emPAI:** 0.06
CG1440, isoform A [Drosophila melanogaster]

Query	Observed	Mr(expt)	Mr(calc)	Delta	Miss	Score	Expect	Rank	Unique	Peptide
15015	746.5173	2236.5302	2236.8925	-0.3624	0	49	0.026	1	U	M.SDNNSGSGGGGSGGGGAGNNNSANSGK.N

---

7. gi|24651407 **Mass:** 63075 **Score:** 47 **Matches:** 2(2) **Sequences:** 2(2) **emPAI:** 0.11
prolyl-4-hydroxylase-alpha EFB [Drosophila melanogaster]

Query	Observed	Mr(expt)	Mr(calc)	Delta	Miss	Score	Expect	Rank	Unique	Peptide
10488	541.1962	1080.3779	1079.4077	0.9702	0	45	0.045	1	U	K.GDDGTDEMPK.S
15695	844.5203	1687.0260	1686.7308	0.2952	0	47	0.045	1	U	R.RPCDLEEDHGEFAI.-

Proteins matching the same set of peptides:

gi|195505190 **Mass:** 55776 **Score:** 47 **Matches:** 2(2) **Sequences:** 2(2)
GE10881 [Drosophila yakuba]

---

8. gi|195161605 **Mass:** 46663 **Score:** 47 **Matches:** 1(1) **Sequences:** 1(1) **emPAI:** 0.07
GL26392 [Drosophila persimilis]

Query	Observed	Mr(expt)	Mr(calc)	Delta	Miss	Score	Expect	Rank	Unique	Peptide
14284	712.2616	1422.5086	1422.7428	-0.2342	1	47	0.042	1	U	K.LIESMEKFLEGK.L

Proteins matching the same set of peptides:
gi|198472755 **Mass:** 46598 **Score:** 47 **Matches:** 1(1) **Sequences:** 1(1)
GA14329 [Drosophila pseudoobscura pseudoobscura]

---

9. gi|195450712 **Mass:** 342814 **Score:** 45 **Matches:** 1(0) **Sequences:** 1(0) **emPAI:** 0.01
GK13603 [Drosophila willistoni]

Query	Observed	Mr(expt)	Mr(calc)	Delta	Miss	Score	Expect	Rank	Unique	Peptide
15099	751.6011	1501.1876	1501.7922	-0.6046	1	45	0.057	1	U	K.SCELLDIQQRIK.S

Mascot: http://www.matrixscience.com/

Mascot Search Results

D17

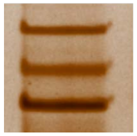

C

```
User                    :
Email                   :
Search title            :
MS data file            : ZIEN-29Nov2017_flyC.mgf
Database                : NCBInr 20120419 (17893860 sequences; 6141683785 residues)
Taxonomy                : Drosophila (fruit flies) (222527 sequences)
Timestamp               : 30 Nov 2017 at 19:12:36 GMT
Enzyme                  : Trypsin
Variable modifications  : Carbamidomethyl (C),Oxidation (M)
Mass values             : Monoisotopic
Protein Mass            : Unrestricted
Peptide Mass Tolerance  : ± 1.25 Da
Fragment Mass Tolerance: ± 1.001 Da
Max Missed Cleavages    : 1
Instrument type         : Default
Number of queries       : 16295
Protein hits            : gi|195590397 GD12572 [Drosophila simulans]
                          gi|17981717  catalase [Drosophila melanogaster]
                          gi|195022939 GH14374 [Drosophila grimshawi]
                          gi|8444      unnamed protein product [Drosophila melanogaster]
                          gi|287945    ATP synthase beta subunit [Drosophila melanogaster]
                          gi|19921848  CG8258 [Drosophila melanogaster]
                          gi|24655737  beta-Tubulin at 56D, isoform B [Drosophila melanogaster]
                          gi|24651407  prolyl-4-hydroxylase-alpha EFB [Drosophila melanogaster]
                          gi|195385695 GJ11603 [Drosophila virilis]
                          gi|195438453 GK24169 [Drosophila willistoni]
                          gi|3108349   pyruvate kinase [Drosophila melanogaster]
                          gi|195434210 GK19056 [Drosophila willistoni]
                          gi|21356731  CG5112 [Drosophila melanogaster]
                          gi|195395688 GJ10965 [Drosophila virilis]
                          gi|195434441 GK14789 [Drosophila willistoni]
                          gi|194763631 GF20995 [Drosophila ananassae]
                          gi|194901836 GG18651 [Drosophila erecta]
                          gi|24648478  KaiRIA [Drosophila melanogaster]
                          gi|195023900 GH20904 [Drosophila grimshawi]
                          gi|195488195 GE14061 [Drosophila yakuba]
                          gi|195126431 GI13073 [Drosophila mojavensis]
                          gi|16554779  no on or off transient A [Drosophila virilis]
```

Select Summary Report

Format As | Select Summary (protein hits) ▼ | Help

Significance threshold p< 0.05 Max. number of hits AUTO

Standard scoring ○ MudPIT scoring ◉ Ions score or expect cut-off 44 Show sub-sets 0

Show pop-ups ◉ Suppress pop-ups ○ Require bold red ☐

Re-Search ◉ All queries ○ Unassigned ○ Below homology threshold ○ Below identity threshold

1. gi|195590397 **Mass:** 55745 **Score:** 238 **Matches:** 12(12) **Sequences:** 8(8) **emPAI:** 0.58
GD12572 [Drosophila simulans]

Query	Observed	Mr(expt)	Mr(calc)	Delta	Miss	Score	Expect	Rank	Unique	Peptide
12656	608.6249	1215.2352	1214.6659	0.5693	0	58	0.0033	1	U	R.QAADIIAWVTK.K 12647
13773	669.4131	1336.8116	1336.6180	0.1936	0	68	0.00042	1	U	K.MDSTANELESIK.I
14382	694.6771	1387.3397	1387.7347	-0.3950	0	56	0.0062	1	U	K.QLAPIYDQLAEK.Y 14377
14981	719.0861	1436.1577	1435.7195	0.4382	0	46	0.046	1	U	K.DLTSVADAEQFLK.D
15648	754.4683	1506.9221	1506.7930	0.1292	0	57	0.0027	1	U	K.VLVSSNFESVALDK.S
15743	760.4873	1518.9600	1518.7930	0.1671	0	54	0.0087	1	U	K.VEEGVLVATVDNFK.Q 15736 15740
16281	1115.7028	2229.3910	2229.1430	0.2481	0	72	8.8e-005	1	U	K.FAQVQSLPLIVDFNHESASK.I
16021	787.0250	2358.0532	2357.2379	0.8153	1	58	0.0036	1	U	K.KFAQVQSLPLIVDFNHESASK.I

Proteins matching the same set of peptides:

gi|17647799 **Mass:** 55746 **Score:** 238 **Matches:** 12(12) **Sequences:** 8(8)
protein disulfide isomerase, isoform A [Drosophila melanogaster]

gi|194751557 **Mass:** 55553 **Score:** 238 **Matches:** 12(12) **Sequences:** 8(8)
GF10739 [Drosophila ananassae]

gi|194871212 **Mass:** 55744 **Score:** 238 **Matches:** 12(12) **Sequences:** 8(8)
GG13681 [Drosophila erecta]

gi|195327719 **Mass:** 55772 **Score:** 238 **Matches:** 12(12) **Sequences:** 8(8)
GM24501 [Drosophila sechellia]

---

2. gi|17981717 **Mass:** 57113 **Score:** 88 **Matches:** 3(3) **Sequences:** 3(3) **emPAI:** 0.18
catalase [Drosophila melanogaster]

Query	Observed	Mr(expt)	Mr(calc)	Delta	Miss	Score	Expect	Rank	Unique	Peptide
12231	589.4701	1176.9256	1176.6060	0.3196	0	46	0.048	1	U	R.MLTEELNLAK.S
12891	619.3951	1236.7757	1236.5986	0.1771	0	45	0.051	1	U	R.DAASNQLIDYK.N
15036	721.9436	1441.8726	1440.6481	1.2245	0	83	6.4e-006	1		R.FSTVGGESGSADTAR.D

Proteins matching the same set of peptides:

gi|194871234 **Mass:** 57183 **Score:** 88 **Matches:** 3(3) **Sequences:** 3(3)

GG13679 [Drosophila erecta]

gi|195352234 **Mass:** 57134 **Score:** 88 **Matches:** 3(3) **Sequences:** 3(3)
Cat [Drosophila sechellia]

gi|195494497 **Mass:** 57064 **Score:** 88 **Matches:** 3(3) **Sequences:** 3(3)
GE19975 [Drosophila yakuba]

gi|195591356 **Mass:** 57134 **Score:** 88 **Matches:** 3(3) **Sequences:** 3(3)
catalase [Drosophila simulans]

---

3. gi|195022939 **Mass:** 57059 **Score:** 86 **Matches:** 2(2) **Sequences:** 2(2) **emPAI:** 0.12
GH14374 [Drosophila grimshawi]

Query	Observed	Mr(expt)	Mr(calc)	Delta	Miss	Score	Expect	Rank	Unique	Peptide
12891	619.3951	1236.7757	1236.5986	0.1771	0	45	0.051	1	U	R.DAASNQLLDYK.N
15036	721.9436	1441.8726	1440.6481	1.2245	0	83	6.4e-006	1		R.FSTVGGESGSADTAR.D

Proteins matching the same set of peptides:

gi|195378516 **Mass:** 57117 **Score:** 86 **Matches:** 2(2) **Sequences:** 2(2)
GJ11574 [Drosophila virilis]

---

4. gi|8444 **Mass:** 62291 **Score:** 84 **Matches:** 2(2) **Sequences:** 2(2) **emPAI:** 0.11
unnamed protein product [Drosophila melanogaster]

Query	Observed	Mr(expt)	Mr(calc)	Delta	Miss	Score	Expect	Rank	Unique	Peptide
12806	**615.2691**	**1228.5236**	**1228.6775**	**-0.1539**	**0**	**64**	**0.0013**	**1**	**U**	**K.SNILVATDVAAR.G**
13278	**640.6392**	**1279.2638**	**1278.7296**	**0.5342**	**0**	**62**	**0.0014**	**1**	**U**	**R.GDGPIALVLAPTR.E**

Proteins matching the same set of peptides:

gi|24644479 **Mass:** 78500 **Score:** 84 **Matches:** 2(2) **Sequences:** 2(2)
Rm62, isoform A [Drosophila melanogaster]

gi|24644481 **Mass:** 62436 **Score:** 84 **Matches:** 2(2) **Sequences:** 2(2)
Rm62, isoform D [Drosophila melanogaster]

gi|24644483 **Mass:** 62791 **Score:** 84 **Matches:** 2(2) **Sequences:** 2(2)
Rm62, isoform E [Drosophila melanogaster]

gi|24644485 **Mass:** 62829 **Score:** 84 **Matches:** 2(2) **Sequences:** 2(2)
Rm62, isoform C [Drosophila melanogaster]

gi|194745414 **Mass:** 77004 **Score:** 84 **Matches:** 2(2) **Sequences:** 2(2)
GF18634 [Drosophila ananassae]

gi|194898941 **Mass:** 78362 **Score:** 84 **Matches:** 2(2) **Sequences:** 2(2)
GG10666 [Drosophila erecta]

gi|195144578 **Mass:** 71625 **Score:** 84 **Matches:** 2(2) **Sequences:** 2(2)
GL23489 [Drosophila persimilis]

gi|195343855 **Mass:** 78805 **Score:** 84 **Matches:** 2(2) **Sequences:** 2(2)
GM10578 [Drosophila sechellia]
gi|195453112 **Mass:** 81548 **Score:** 84 **Matches:** 2(2) **Sequences:** 2(2)
GK14214 [Drosophila willistoni]
gi|195502160 **Mass:** 78418 **Score:** 84 **Matches:** 2(2) **Sequences:** 2(2)
GE24123 [Drosophila yakuba]
gi|198452778 **Mass:** 79875 **Score:** 84 **Matches:** 2(2) **Sequences:** 2(2)
GA10214 [Drosophila pseudoobscura pseudoobscura]
gi|383292518 **Mass:** 50067 **Score:** 84 **Matches:** 2(2) **Sequences:** 2(2)
Rm62, isoform J [Drosophila melanogaster]
gi|383292519 **Mass:** 53163 **Score:** 84 **Matches:** 2(2) **Sequences:** 2(2)
Rm62, isoform K [Drosophila melanogaster]
gi|383292520 **Mass:** 51999 **Score:** 84 **Matches:** 2(2) **Sequences:** 2(2)
Rm62, isoform L [Drosophila melanogaster]
gi|383292521 **Mass:** 52514 **Score:** 84 **Matches:** 2(2) **Sequences:** 2(2)
Rm62, isoform M [Drosophila melanogaster]
gi|383873392 **Mass:** 33568 **Score:** 84 **Matches:** 2(2) **Sequences:** 2(2)
MIP33508p1 [Drosophila melanogaster]
gi|195109284 **Mass:** 79484 **Score:** 84 **Matches:** 2(2) **Sequences:** 2(2)
GI23184 [Drosophila mojavensis]

---

5. gi|287945 **Mass:** 53487 **Score:** 63 **Matches:** 2(2) **Sequences:** 2(2) **emPAI:** 0.13
ATP synthase beta subunit [Drosophila melanogaster]

Query	Observed	Mr(expt)	Mr(calc)	Delta	Miss	Score	Expect	Rank	Unique	Peptide
10435	**517.1084**	**1032.2022**	**1032.5604**	**-0.3582**	**0**	**46**	**0.035**	**1**	**U**	**K.VLDTGYPIR.I**
14074	**684.3671**	**1366.7196**	**1366.7456**	**-0.0260**	**0**	**61**	**0.0019**	**1**	**U**	**R.IINVIGEPIDER.G**

Proteins matching the same set of peptides:
gi|24638766 **Mass:** 54074 **Score:** 63 **Matches:** 2(2) **Sequences:** 2(2)
ATP synthase-beta, isoform A [Drosophila melanogaster]
gi|38048209 **Mass:** 21234 **Score:** 63 **Matches:** 2(2) **Sequences:** 2(2)
similar to Drosophila melanogaster ATPsyn-beta, partial [Drosophila yakuba]
gi|38048553 **Mass:** 21774 **Score:** 63 **Matches:** 2(2) **Sequences:** 2(2)
similar to Drosophila melanogaster ATPsyn-beta, partial [Drosophila yakuba]
gi|194770714 **Mass:** 54254 **Score:** 63 **Matches:** 2(2) **Sequences:** 2(2)
GF21882 [Drosophila ananassae]
gi|195064082 **Mass:** 54196 **Score:** 63 **Matches:** 2(2) **Sequences:** 2(2)
GH23965 [Drosophila grimshawi]

gi|195134016 **Mass:** 54192 **Score:** 63 **Matches:** 2(2) **Sequences:** 2(2)
GI14045 [Drosophila mojavensis]
gi|195355680 **Mass:** 41109 **Score:** 63 **Matches:** 2(2) **Sequences:** 2(2)
GM13022 [Drosophila sechellia]
gi|195450700 **Mass:** 54274 **Score:** 63 **Matches:** 2(2) **Sequences:** 2(2)
GK13685 [Drosophila willistoni]
gi|195469417 **Mass:** 54104 **Score:** 63 **Matches:** 2(2) **Sequences:** 2(2)
ATPsyn-beta [Drosophila yakuba]
gi|195564346 **Mass:** 47247 **Score:** 63 **Matches:** 2(2) **Sequences:** 2(2)
ATPsyn-beta [Drosophila simulans]

---

6. gi|19921848 **Mass:** 59396 **Score:** 62 **Matches:** 2(2) **Sequences:** 2(2) **emPAI:** 0.11
CG8258 [Drosophila melanogaster]

Query	Observed	Mr(expt)	Mr(calc)	Delta	Miss	Score	Expect	Rank	Unique	Peptide
11419	560.9957	1119.9768	1119.5560	0.4208	0	46	0.056	1	U	R.ALDDAINNFK.C
15559	749.0649	1496.1153	1495.7406	0.3747	0	57	0.0039	1	U	R.LGITTAEISDGYEK.A

---

7. gi|24655737 **Mass:** 50115 **Score:** 62 **Matches:** 2(2) **Sequences:** 2(2) **emPAI:** 0.14
beta-Tubulin at 56D, isoform B [Drosophila melanogaster]

Query	Observed	Mr(expt)	Mr(calc)	Delta	Miss	Score	Expect	Rank	Unique	Peptide
13754	668.3984	1334.7823	1334.6904	0.0919	0	53	0.012	1	U	R.IMNTYSVVPSPK.V
16093	801.2383	1600.4620	1600.8131	-0.3511	0	50	0.017	1	U	R.AVLVDLEPGTMDSVR.S

Proteins matching the same set of peptides:
gi|24655741 **Mass:** 51264 **Score:** 62 **Matches:** 2(2) **Sequences:** 2(2)
beta-Tubulin at 56D, isoform A [Drosophila melanogaster]
gi|188529361 **Mass:** 20477 **Score:** 62 **Matches:** 2(2) **Sequences:** 2(2)
beta-tubulin [Drosophila silvestris]
gi|194881355 **Mass:** 53339 **Score:** 62 **Matches:** 2(2) **Sequences:** 2(2)
GG20906 [Drosophila erecta]
gi|306922435 **Mass:** 51322 **Score:** 62 **Matches:** 2(2) **Sequences:** 2(2)
GH12877p [Drosophila melanogaster]

---

8. gi|24651407 **Mass:** 63075 **Score:** 60 **Matches:** 1(1) **Sequences:** 1(1) **emPAI:** 0.05
prolyl-4-hydroxylase-alpha EFB [Drosophila melanogaster]

Query	Observed	Mr(expt)	Mr(calc)	Delta	Miss	Score	Expect	Rank	Unique	Peptide
16080	799.1600	1596.3055	1595.7791	0.5264	0	60	0.0017	1	U	R.LQDTYQLDTSSVAR.G

Proteins matching the same set of peptides:

gi|125772807 **Mass:** 62641 **Score:** 60 **Matches:** 1(1) **Sequences:** 1(1)
GA15946 [Drosophila pseudoobscura pseudoobscura]

gi|194765194 **Mass:** 62567 **Score:** 60 **Matches:** 1(1) **Sequences:** 1(1)
GF22904 [Drosophila ananassae]

gi|194905436 **Mass:** 62737 **Score:** 60 **Matches:** 1(1) **Sequences:** 1(1)
GG11753 [Drosophila erecta]

gi|195055779 **Mass:** 55833 **Score:** 60 **Matches:** 1(1) **Sequences:** 1(1)
GH14110 [Drosophila grimshawi]

gi|195159323 **Mass:** 55783 **Score:** 60 **Matches:** 1(1) **Sequences:** 1(1)
GL13463 [Drosophila persimilis]

gi|195341536 **Mass:** 63009 **Score:** 60 **Matches:** 1(1) **Sequences:** 1(1)
GM12882 [Drosophila sechellia]

gi|195391754 **Mass:** 55603 **Score:** 60 **Matches:** 1(1) **Sequences:** 1(1)
GJ24502 [Drosophila virilis]

gi|195452726 **Mass:** 62836 **Score:** 60 **Matches:** 1(1) **Sequences:** 1(1)
GK14136 [Drosophila willistoni]

gi|195505190 **Mass:** 55776 **Score:** 60 **Matches:** 1(1) **Sequences:** 1(1)
GE10881 [Drosophila yakuba]

gi|195575089 **Mass:** 62991 **Score:** 60 **Matches:** 1(1) **Sequences:** 1(1)
GD21521 [Drosophila simulans]

---

9. gi|195385695 **Mass:** 47177 **Score:** 60 **Matches:** 2(2) **Sequences:** 1(1) **emPAI:** 0.07
GJ11603 [Drosophila virilis]

Query	Observed	Mr(expt)	Mr(calc)	Delta	Miss	Score	Expect	Rank	Unique	Peptide
13981	680.0380	1358.0614	1358.7228	-0.6614	1	54	0.0044	1	U	-.MSKQLADLQPTK.F 13982

---

10. gi|195438453 **Mass:** 56787 **Score:** 52 **Matches:** 1(1) **Sequences:** 1(1) **emPAI:** 0.06
GK24169 [Drosophila willistoni]

Query	Observed	Mr(expt)	Mr(calc)	Delta	Miss	Score	Expect	Rank	Unique	Peptide
12945	622.3690	1242.7234	1241.6364	1.0870	0	52	0.018	1	U	K.NQDVAAVQEIR.E

Proteins matching the same set of peptides:

gi|17137450 **Mass:** 55970 **Score:** 52 **Matches:** 1(1) **Sequences:** 1(1)
regulatory particle non-ATPase 3 [Drosophila melanogaster]

gi|19527921 **Mass:** 55992 **Score:** 52 **Matches:** 1(1) **Sequences:** 1(1)
AT15146p [Drosophila melanogaster]

gi|125986955 **Mass:** 56336 **Score:** 52 **Matches:** 1(1) **Sequences:** 1(1)
GA10344 [Drosophila pseudoobscura pseudoobscura]

gi|170280395 **Mass:** 26416 **Score:** 52 **Matches:** 1(1) **Sequences:** 1(1)
diphenol oxidase A2, partial [Drosophila erecta]
gi|170280397 **Mass:** 26437 **Score:** 52 **Matches:** 1(1) **Sequences:** 1(1)
diphenol oxidase A2, partial [Drosophila mauritiana]
gi|170280399 **Mass:** 26398 **Score:** 52 **Matches:** 1(1) **Sequences:** 1(1)
diphenol oxidase A2, partial [Drosophila melanogaster]
gi|170280401 **Mass:** 26430 **Score:** 52 **Matches:** 1(1) **Sequences:** 1(1)
diphenol oxidase A2, partial [Drosophila santomea]
gi|170280403 **Mass:** 26509 **Score:** 52 **Matches:** 1(1) **Sequences:** 1(1)
diphenol oxidase A2, partial [Drosophila sechellia]
gi|170280405 **Mass:** 26414 **Score:** 52 **Matches:** 1(1) **Sequences:** 1(1)
diphenol oxidase A2, partial [Drosophila simulans]
gi|170280407 **Mass:** 26458 **Score:** 52 **Matches:** 1(1) **Sequences:** 1(1)
diphenol oxidase A2, partial [Drosophila teissieri]
gi|194879789 **Mass:** 56011 **Score:** 52 **Matches:** 1(1) **Sequences:** 1(1)
GG21155 [Drosophila erecta]
gi|195156337 **Mass:** 56318 **Score:** 52 **Matches:** 1(1) **Sequences:** 1(1)
GL26159 [Drosophila persimilis]
gi|195345003 **Mass:** 56051 **Score:** 52 **Matches:** 1(1) **Sequences:** 1(1)
GM17319 [Drosophila sechellia]
gi|195484349 **Mass:** 56025 **Score:** 52 **Matches:** 1(1) **Sequences:** 1(1)
GE13227 [Drosophila yakuba]
gi|195580065 **Mass:** 56028 **Score:** 52 **Matches:** 1(1) **Sequences:** 1(1)
GD24178 [Drosophila simulans]

---

11. gi|3108349 **Mass:** 57437 **Score:** 50 **Matches:** 1(1) **Sequences:** 1(1) **emPAI:** 0.06
pyruvate kinase [Drosophila melanogaster]

Query	Observed	Mr(expt)	Mr(calc)	Delta	Miss	Score	Expect	Rank	Unique	Peptide
11674	**571.3002**	**1140.5859**	**1140.6026**	**-0.0167**	**0**	**50**	**0.031**	**1**	**U**	**R.GDLGIEIPAEK.V**

Proteins matching the same set of peptides:
gi|24648964 **Mass:** 55024 **Score:** 50 **Matches:** 1(1) **Sequences:** 1(1)
pyruvate kinase, isoform B [Drosophila melanogaster]
gi|27819773 **Mass:** 57438 **Score:** 50 **Matches:** 1(1) **Sequences:** 1(1)
RH07636p [Drosophila melanogaster]
gi|28571814 **Mass:** 57404 **Score:** 50 **Matches:** 1(1) **Sequences:** 1(1)
pyruvate kinase, isoform A [Drosophila melanogaster]
gi|38047755 **Mass:** 19755 **Score:** 50 **Matches:** 1(1) **Sequences:** 1(1)

similar to Drosophila melanogaster PyK, partial [Drosophila yakuba]

gi|194744590 **Mass:** 57203 **Score:** 50 **Matches:** 1(1) **Sequences:** 1(1)
GF18439 [Drosophila ananassae]

gi|194911138 **Mass:** 57502 **Score:** 50 **Matches:** 1(1) **Sequences:** 1(1)
GG11123 [Drosophila erecta]

gi|195053328 **Mass:** 57522 **Score:** 50 **Matches:** 1(1) **Sequences:** 1(1)
GH20329 [Drosophila grimshawi]

gi|195056081 **Mass:** 57549 **Score:** 50 **Matches:** 1(1) **Sequences:** 1(1)
GH13278 [Drosophila grimshawi]

gi|195112292 **Mass:** 57581 **Score:** 50 **Matches:** 1(1) **Sequences:** 1(1)
GI22374 [Drosophila mojavensis]

gi|195145138 **Mass:** 57003 **Score:** 50 **Matches:** 1(1) **Sequences:** 1(1)
GL24201 [Drosophila persimilis]

gi|195330979 **Mass:** 57418 **Score:** 50 **Matches:** 1(1) **Sequences:** 1(1)
GM26420 [Drosophila sechellia]

gi|195391442 **Mass:** 102583 **Score:** 50 **Matches:** 1(1) **Sequences:** 1(1)
GJ24408 [Drosophila virilis]

gi|195453308 **Mass:** 57517 **Score:** 50 **Matches:** 1(1) **Sequences:** 1(1)
GK14262 [Drosophila willistoni]

gi|195502545 **Mass:** 57418 **Score:** 50 **Matches:** 1(1) **Sequences:** 1(1)
PyK [Drosophila yakuba]

gi|198452351 **Mass:** 57033 **Score:** 50 **Matches:** 1(1) **Sequences:** 1(1)
GA26534 [Drosophila pseudoobscura pseudoobscura]

---

12. gi|195434210 **Mass:** 21374 **Score:** 49 **Matches:** 1(1) **Sequences:** 1(1) **emPAI:** 0.16
GK19056 [Drosophila willistoni]

Query	Observed	Mr(expt)	Mr(calc)	Delta	Miss	Score	Expect	Rank	Unique	Peptide
12267	590.4731	1178.9317	1178.5866	0.3451	0	49	0.013	1	U	K.YQLIQMNNR.S

---

13. gi|21356731 **Mass:** 58448 **Score:** 49 **Matches:** 1(1) **Sequences:** 1(1) **emPAI:** 0.06
CG5112 [Drosophila melanogaster]

Query	Observed	Mr(expt)	Mr(calc)	Delta	Miss	Score	Expect	Rank	Unique	Peptide
11365	558.2457	1114.4769	1113.6142	0.8627	0	49	0.022	1	U	K.SAVELAQQIR.E

Proteins matching the same set of peptides:

gi|125776626 **Mass:** 58393 **Score:** 49 **Matches:** 1(1) **Sequences:** 1(1)
GA18668 [Drosophila pseudoobscura pseudoobscura]

gi|194908530 **Mass:** 58327 **Score:** 49 **Matches:** 1(1) **Sequences:** 1(1)

GG11409 [Drosophila erecta]
gi|195152431 **Mass:** 58361 **Score:** 49 **Matches:** 1(1) **Sequences:** 1(1)
GL22143 [Drosophila persimilis]
gi|195349457 **Mass:** 58431 **Score:** 49 **Matches:** 1(1) **Sequences:** 1(1)
GM10248 [Drosophila sechellia]
gi|195453896 **Mass:** 58368 **Score:** 49 **Matches:** 1(1) **Sequences:** 1(1)
GK12847 [Drosophila willistoni]
gi|195504320 **Mass:** 58393 **Score:** 49 **Matches:** 1(1) **Sequences:** 1(1)
GE23605 [Drosophila yakuba]
gi|195573909 **Mass:** 58441 **Score:** 49 **Matches:** 1(1) **Sequences:** 1(1)
GD21221 [Drosophila simulans]

---

14. gi|195395688 **Mass:** 58173 **Score:** 49 **Matches:** 1(1) **Sequences:** 1(1) **emPAI:** 0.06
GJ10965 [Drosophila virilis]

Query	Observed	Mr(expt)	Mr(calc)	Delta	Miss	Score	Expect	Rank	Unique	Peptide
11365	558.2457	1114.4769	1113.6506	0.8263	1	49	0.022	1	U	K.SAVELAKQIR.E

---

15. gi|195434441 **Mass:** 51967 **Score:** 48 **Matches:** 1(1) **Sequences:** 1(1) **emPAI:** 0.06
GK14789 [Drosophila willistoni]

Query	Observed	Mr(expt)	Mr(calc)	Delta	Miss	Score	Expect	Rank	Unique	Peptide
14049	**683.3903**	**1364.7660**	**1364.6295**	**0.1364**	**0**	**48**	**0.041**	**1**	**U**	**K.MDSHFNAFQLR.N**

---

16. gi|194763631 **Score:** 48 **Matches:** 1(1) **Sequences:** 1(1) **emPAI:** 0.03
GF20995 [Drosophila ananassae]

Query	Observed	Mr(expt)	Mr(calc)	Delta	Miss	Score	Expect	Rank	Unique	Peptide
13278	640.6392	1279.2638	1278.7296	0.5342	0	48	0.036	2	U	R.GEGPVALVLAPTR.E

Proteins matching the same set of peptides:
gi|195438679 **Score:** 48 **Matches:** 1(1) **Sequences:** 1(1)

---

17. gi|194901836 **Mass:** 5994 **Score:** 47 **Matches:** 1(1) **Sequences:** 1(1) **emPAI:** 0.60
GG18651 [Drosophila erecta]

Query	Observed	Mr(expt)	Mr(calc)	Delta	Miss	Score	Expect	Rank	Unique	Peptide
13236	**638.0350**	**1274.0554**	**1273.5760**	**0.4794**	**1**	**47**	**0.046**	**1**	**U**	**K.KGGGGGGGGGGGGSGNK.N**

---

18. gi|24648478 **Mass:** 101593 **Score:** 46 **Matches:** 1(1) **Sequences:** 1(1) **emPAI:** 0.03
KaiRIA [Drosophila melanogaster]

Query	Observed	Mr(expt)	Mr(calc)	Delta	Miss	Score	Expect	Rank	Unique	Peptide
13749	668.1045	1334.1944	1334.6790	-0.4845	1	46	0.043	1	U	R.SRNSSQSIESLK.T

Proteins matching the same set of peptides:

gi|61675663 **Mass:** 101564 **Score:** 46 **Matches:** 1(1) **Sequences:** 1(1)
RE24732p [Drosophila melanogaster]

gi|194899712 **Mass:** 101589 **Score:** 46 **Matches:** 1(1) **Sequences:** 1(1)
GG15269 [Drosophila erecta]

gi|195353927 **Mass:** 86610 **Score:** 46 **Matches:** 1(1) **Sequences:** 1(1)
GM23173 [Drosophila sechellia]

gi|195498265 **Mass:** 101563 **Score:** 46 **Matches:** 1(1) **Sequences:** 1(1)
GE25051 [Drosophila yakuba]

gi|195569399 **Mass:** 101626 **Score:** 46 **Matches:** 1(1) **Sequences:** 1(1)
GD20046 [Drosophila simulans]

---

19. gi|195023900 **Mass:** 190030 **Score:** 46 **Matches:** 1(1) **Sequences:** 1(1) **emPAI:** 0.02
GH20904 [Drosophila grimshawi]

Query	Observed	Mr(expt)	Mr(calc)	Delta	Miss	Score	Expect	Rank	Unique	Peptide
12330	593.1379	1184.2612	1184.6302	-0.3690	1	46	0.029	1	U	K.VFHLGGGGDAKK.K

---

20. gi|195488195 **Mass:** 66917 **Score:** 46 **Matches:** 1(1) **Sequences:** 1(1) **emPAI:** 0.05
GE14061 [Drosophila yakuba]

Query	Observed	Mr(expt)	Mr(calc)	Delta	Miss	Score	Expect	Rank	Unique	Peptide
15913	775.3869	1548.7593	1549.7554	-0.9961	1	46	0.039	1	U	R.MEPMKDLMAVLEK.L

---

21. gi|195126431 **Mass:** 66814 **Score:** 46 **Matches:** 1(1) **Sequences:** 1(1) **emPAI:** 0.05
GI13073 [Drosophila mojavensis]

Query	Observed	Mr(expt)	Mr(calc)	Delta	Miss	Score	Expect	Rank	Unique	Peptide
10435	517.1084	1032.2022	1032.5604	-0.3582	0	46	0.035	1	U	K.VIDTGYPIR.V

Proteins matching the same set of peptides:

gi|195442758 **Mass:** 74458 **Score:** 46 **Matches:** 1(1) **Sequences:** 1(1)
GK24170 [Drosophila willistoni]

---

22. gi|16554779 **Mass:** 15812 **Score:** 44 **Matches:** 1(0) **Sequences:** 1(0) **emPAI:** 0.22
no on or off transient A [Drosophila virilis]

Query	Observed	Mr(expt)	Mr(calc)	Delta	Miss	Score	Expect	Rank	Unique	Peptide

15089	723.9612	2168.8617	2168.9154	-0.0537	1	44	0.086	1	U	R.ARGGGGGGGGGGGGGGGGGGGGGGGGGGGGGGGR.D

Mascot: http://www.matrixscience.com/
