# Supplementary material for: Evidence for the major role of PH4αEFB in the prolyl 4-hydroxylation of Drosophila collagen IV
Source: Matrix Biol. Author manuscript; Available in PMC 2025 Dec 28. (PMC12744885; doi:10.1016/j.matbio.2025.09.002)
Supplement: Fig S1 [file NIHMS2129658-supplement-Fig_S1.pdf]

Fat body

- muscle cell
- adult fat body
- adult neuron
- adult oenocyte
- epithelial cell
- female reproductive system
- hemocyte
- hemocyte(*)
- ovary cell
- unannotated

Ovary

- central main body follicle cell ca. St. 6-8
- choriogenic main body follicle cell and corpus luteum
- choriogenic main body follicle cell St. 12
- choriogenic main body follicle cell St. 14
- dorsal appendage forming follicle cell
- main body follicle cell ca. until St. 5
- oviduct
- posterior terminal follicle cell ca. St. 5-8
- stretch follicle cell
- unannotated

Whole body

- adult fat body
- adult glial cell
- adult hindgut
- adult oenocyte
- adult peripheral nervous system
- adult reticular neuropil associated glial cell
- adult tracheocyte
- adult ventral nervous system
- artefact
- cell body glial cell
- enteroendocrine cell
- eo support cell
- epithelial cell
- escort cell
- female reproductive system
- follicle cell
- follicle cell St. 9+
- germline cell
- gustatory receptor neuron
- hemocyte
- indirect flight muscle
- leg muscle motor neuron
- leg taste bristle chemosensory neuron
- male accessory gland
- muscle cell
- perineurial glial sheath
- scolopidial neuron
- subperineurial glial cell
- unannotated

Fig. S1. Full legend for the symbols in Fig. 3B.
